# Identification of genetic variants that impact gene co-expression relationships using large-scale single-cell data

**DOI:** 10.1186/s13059-023-02897-x

**Published:** 2023-04-18

**Authors:** Shuang Li, Katharina T. Schmid, Dylan H. de Vries, Maryna Korshevniuk, Corinna Losert, Roy Oelen, Irene V. van Blokland, BIOS Consortium, sc-eQTLgen Consortium, Hilde E. Groot, Morris A. Swertz, Pim van der Harst, Harm-Jan Westra, Monique G.P. van der Wijst, Matthias Heinig, Lude Franke

**Affiliations:** 1 Genetics Department, University Medical Center Groningen, Groningen, the Netherlands; 2 Genomics Coordination Center, University of Groningen, University Medical Center Groningen, Groningen, the Netherlands; 3 Wellcome Sanger Institute, Wellcome Genome Campus, Hinxton, UK; 4 Institute of Computational Biology, Helmholtz Center Munich, Munich, Germany; 5 Department of Computer Science, School of Computation, Information and Technology, Technical University Munich, Munich, Germany; 6 Department of Cardiology, University of Groningen, University Medical Center Groningen, Groningen, the Netherlands; 7 Department of Cardiology, University Medical Center Utrecht, Utrecht, the Netherlands; 8 Munich Heart Alliance, DZHK (German Center for Cardiovascular Research), Munich, Germany

**Keywords:** Co-expression QTLs, scRNA-seq, eQTL

## Abstract

**Background:**

Expression quantitative trait loci (eQTL) studies show how genetic variants affect downstream gene expression. Single-cell data allows reconstruction of personalized co-expression networks and therefore the identification of SNPs altering co-expression patterns (co-expression QTLs, co-eQTLs) and the affected upstream regulatory processes using a limited number of individuals.

**Results:**

We conduct a co-eQTL meta-analysis across four scRNA-seq peripheral blood mononuclear cell datasets using a novel filtering strategy followed by a permutation-based multiple testing approach. Before the analysis, we evaluate the co-expression patterns required for co-eQTL identification using different external resources. We identify a robust set of cell-type-specific co-eQTLs for 72 independent SNPs affecting 946 gene pairs. These co-eQTLs are replicated in a large bulk cohort and provide novel insights into how disease-associated variants alter regulatory networks. One co-eQTL SNP, rs1131017, that is associated with several autoimmune diseases, affects the co-expression of RPS26 with other ribosomal genes. Interestingly, specifically in T cells, the SNP additionally affects co-expression of RPS26 and a group of genes associated with T cell activation and autoimmune disease. Among these genes, we identify enrichment for targets of five T-cell-activation-related transcription factors whose binding sites harbor rs1131017. This reveals a previously overlooked process and pinpoints potential regulators that could explain the association of rs1131017 with autoimmune diseases.

**Conclusion:**

Our co-eQTL results highlight the importance of studying context-specific gene regulation to understand the biological implications of genetic variation. With the expected growth of sc-eQTL datasets, our strategy and technical guidelines will facilitate future co-eQTL identification, further elucidating unknown disease mechanisms.

**Supplementary Information:**

The online version contains supplementary material available at 10.1186/s13059-023-02897-x.

## Background

In recent years, genome-wide association studies (GWAS) have revealed a large number of associations between genetic variation and disease [1]. Many of these variants also change downstream gene expression, as identified using expression quantitative trait locus (eQTL) analysis [2]. However, even with many such connections now identified, the upstream biological processes that regulate these eQTLs often remain hidden. Such knowledge is important for better understanding the underlying processes that lead to specific disease, which would aid in drug development [3].

One way to study the biological processes in which eQTL genes are involved is to construct gene co-expression networks. In these networks, genes (nodes) involved in shared biological processes are expected to be connected through co-expression (edges) [4]. Traditionally, these networks have been reconstructed with bulk RNA sequencing (RNA-seq) data, using a variety of computational tools [5–7]. However, whether certain biological processes are active can depend on various factors, such as cell type, environmental exposures, and even single-nucleotide polymorphisms (SNPs) [2, 8, 9]. With single-cell technologies, many of these highly specific contexts can now be captured at high resolution. Single-cell RNA-seq (scRNA-seq) not only allows for cell-type-specific analyses, it does so without the technical biases introduced by the cell sorting required to perform similar analyses with bulk RNA-seq.

In addition to capturing the cell-type-specific contexts, scRNA-seq can also be used to construct personalized co-expression networks using the repeated measurements (i.e., multiple single-cell gene expression profiles) for each individual. This enables quantification of the covariance between genes, and thus their co-expression strengths, within an individual [10]. These personalized co-expression networks can then be used to study the effects of genetic variation on network properties. Some of these network changes can be linked to individual SNP genotypes, called co-expression QTLs (co-eQTLs).

While we have previously shown that co-eQTLs can be both cell-type-specific and stimulation-specific, several challenges to systematic identification remain [10, 11]. Firstly, it is unclear how to best construct gene regulatory networks (GRNs) with scRNA-seq data. Co-expression patterns identified from bulk RNA-seq data have been shown to be informative for physical and functional gene–gene interactions [5–11], but it is unclear whether the co-expression patterns identified with scRNA-seq data also reflect gene–gene functional interactions given technical challenges of scRNA-seq data such as sparseness and low signal-to-noise ratios [12, 13]. These issues are caused by a combination of low mRNA counts in cells, imperfect capture efficiencies, and the inherent stochasticity of mRNA expression [14]. Many methods have been proposed to account for this issue. A recent benchmark paper suggested “rho proportionality” [15] as an association measure because of its consistent performance [16]. Also complementary strategies could be beneficial, such as combining association measures with *MetaCell*, a recently proposed method that groups homogeneous cells to reduce sparsity, but to our knowledge it has not yet been evaluated in benchmark studies [17]. Moreover, a recent benchmark paper concluded that different GRN construction methods show moderate performance that is often dataset-specific [18], indicating that many challenges remain in GRN reconstruction. Therefore, validation of the robustness and functional relevance of the network is warranted.

Secondly, there is no consensus method for co-eQTL mapping and personalized GRN construction. In bulk data with only one measurement per individual, it is not possible to identify co-eQTLs directly. To carry out a similar type of analysis in bulk data, we previously used a linear regression model with an interaction term to identify interaction QTLs in bulk data from whole blood [8]. This approach can reveal co-eQTLs using the expression levels of individual genes as interaction terms. However, as bulk data nearly always comprises a mixture of cell types, it is not straightforward to unequivocally conclude that eQTLs showing an interaction effect reflect co-eQTLs (genetic variants that affect the co-expression between pairs of genes) rather than a change in cell-type composition. A further compounding problem is that very large numbers of samples are required to identify co-eQTLs, and effects that manifest in specific (rare) cell types can easily be missed because they are masked by more common cell types. In theory, single-cell data allows direct estimation of cell-type-specific and individual-specific co-expression strength and should reduce the sample size requirement compared to bulk datasets. However, in practical terms, there are currently no datasets large enough to provide the statistical power to do genome-wide co-eQTL mapping, as this involves a large multiple testing burden due to billions of tests for every SNP and every possible gene pair combination. As such, there is a clear need for a robust co-eQTL strategy that can overcome the severe multiple testing issues and deal with the aforementioned issues with regard to the construction of reliable personalized co-expression networks.

In this work, we studied the genetic regulation of gene co-expression by conducting the largest-to-date co-eQTL meta-analysis in 173 peripheral blood mononuclear cell (PBMC) scRNA-seq samples. Previous studies [10, 11] focused on a small set of SNP–gene–gene triplets and specific cell types, limiting the number of identified co-eQTL. Compared to previous studies, we have increased the sample size, improved the co-eQTL mapping strategy, and have applied comprehensive interpretation strategies. This enabled a larger search space of SNP–gene–gene triplets, consequently highlighting the cell-type specificity of gene–gene regulation underlying GWAS signals. To make this possible, we first benchmarked various GRN construction methods and compared the obtained co-expression patterns in our scRNA-seq data to two bulk RNA-seq datasets and a CRISPR-coupled scRNA-seq screen knockout dataset [19]. We then studied the effects of cell type and inter-individual differences in gene co-expression networks by reconstructing personalized and cell-type-specific networks. We subsequently developed a robust co-eQTL mapping strategy with a novel filtering approach and a customized permutation-based multiple testing procedure to deal with the correlation structure in the co-expression networks. With the improved strategy, we could perform a co-eQTL meta-analysis using data from three different scRNA-seq studies. We provided a comprehensive analysis of the different factors that affect the quality and quantity of co-eQTLs, including the number of cells, gene expression levels and filtering strategy. We then studied which biological processes and genes are regulated by the identified co-eQTLs by performing different enrichment analyses and exploring common biological functions, transcription factor (TF) binding, and disease associations to pinpoint potential direct regulators of the co-eQTL genes. In sum, our results suggest that the combination of a robust method and a large sample size is crucial for identification of genetic variants that affect co-expression networks.

## Results

### Overview of the study

**Fig. 1:**
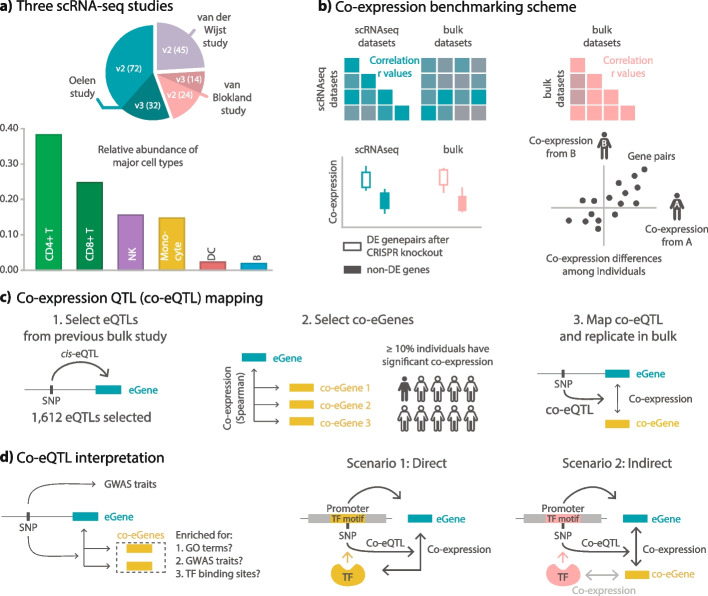
Study overview.** a** Overview of the three PBMC scRNA-seq studies used in our study. The studies, the version of the used chemistry for data generation (version 2 and 3, referred to as v2 and v3, respectively), number of individuals involved (indicated as the number in the parenthesis), and relative composition of the major blood cell types used in this study. **b** Co-expression benchmarking scheme. We first benchmarked co-expression patterns among the three scRNA-seq studies and compared them to co-expression patterns in different bulk datasets. As an additional external validation, we benchmarked both the scRNA-seq and bulk co-expression patterns with a CRISPR knockout dataset. After benchmarking, we evaluated differences in co-expression patterns among cell types and among individuals within a cell type. **c** Co-expression QTL (co-eQTL) mapping. Building on the benchmarked co-expression pattern, we developed a novel strategy to identify co-eQTLs (genetic variants changing co-expression). Part of the strategy is a strict filtering of tested SNP–eGene–co-eGene triplets, where the SNP is required to be an eQTL for one of the genes and the genes show significant correlation in at least a certain number of individuals. **d** Co-expression QTL (co-eQTL) insights. We first check if the SNP we tested has been identified in previous GWAS studies, or it is in high LD with GWAS variants. Then we check the group of co-eGenes on what pathways and traits and TF binding sites they are enriched for. We hypothesize two possible scenarios of the underlying biological mechanism for the identified co-eQTLs. Scenario 1 portrays a direct regulatory association between the co-eGene and the eGene through the genetic variants that change the binding affinity. Scenario 2 portrays an indirect association between the co-eGene and the eGene. That is, the co-eGene is co-expressed with the TF that regulates the expression level of the eGene

To uncover the contexts and biological processes that affect gene expression regulation, this study took advantage of both the resolution of single-cell data and the directionality captured by co-eQTLs. First, we constructed cell-type-specific co-expression networks using five scRNA-seq PBMC datasets from three recently generated PBMC scRNA-seq studies [10, 11, 20] totaling 187 individuals and approximately one million cells. Two of the studies contained data that was in part generated using different versions of 10 × chemistry (version 2 or version 3, the donors were measured with either version 2 or version 3 chemistry). To avoid batch effects due to these technical differences, we split both these studies into two datasets, depending on the chemistry, leading to five datasets in total: (1) two datasets from the Oelen study [11] that collected unstimulated PBMCs from 104 healthy individuals from the Northern Netherlands, a dataset measured using version 2 chemistry (Oelen v2 dataset) and one measured using version 3 chemistry (Oelen v3 dataset), (2) a dataset from the van der Wijst study [11] that collected unstimulated PBMCs from 45 healthy individuals from the Northern Netherlands measured using version 2 chemistry (van der Wijst dataset), and (3) two datasets from the van Blokland study [20] that collected unstimulated PBMCs from 38 individuals 6–8 weeks after having a heart attack, one dataset measured using version 2 chemistry (van Blokland v2 dataset) and one measured using version 3 chemistry (van Blokland v3 dataset) (Fig. 1a). Details about these datasets are described in Additional file 1: Table S1.

We focused on the six major cell types (B cells, CD4 + T cells, CD8 + T cells, dendritic cells (DCs), monocytes and natural killer (NK) cells), of which CD4 + T cells, CD8 + T cells and monocytes were the most frequent cell types (Additional file 2: Fig. S1). We compared commonly used measures of correlation and those previously reported to be particularly suitable for capturing co-expression in scRNA-seq data, including rho proportionality [15], the Spearman correlation, and GRNBoost2 [21], and tested complementary strategies such as MetaCell [17]. We validated that the co-expression patterns from our single-cell dataset are enriched for actual gene regulatory relationships by benchmarking the concordance of the co-expression patterns across the three single-cell studies [2, 11, 20] and three cell-type-specific or whole-blood bulk RNA-seq datasets [2, 22, 23] (Fig. 1b). Furthermore, we validated identified connections with a CRISPR dataset [19].

Next, we evaluated the concordance of the co-expression networks between the major blood cell types and between different individuals within each cell type (Fig. 1b). For the comparison of co-expression networks among cell types, we merged data from all individuals before calculating co-expression. For the comparison between individuals, we calculated co-expression per individual. To identify the genetic contribution to such common and cell-type-specific effects, we performed a constrained co-eQTL meta-analysis. For this, we first removed the van Blokland v3 dataset from our analyses as the small number of donors (*n* = 14) was too limited to provide added value for this purpose. After this, we filtered SNPs that exhibit an eQTL effect (with the corresponding gene referred to as an eGene below) and tested all genes with sufficient co-expression strength with the eGene (called co-eGenes below) among different individuals (Fig. 1c).

For the co-eQTL interpretation, we considered different scenarios that can lead to detection of a co-eQTL. One is that the genetic variant changes the binding affinity of a TF and thus the regulation of its target gene, which would cause a co-eQTL between the variant, the TF, and the target gene (Fig. 1c, Scenario 1). However, a co-eQTL will also occur indirectly for all genes in strong correlation with this TF (Fig. 1c, Scenario 2): a gene correlated with the TF will also be correlated with the eGene and this correlation strength will also depend on the genotype because of the indirect association via the TF. To distinguish both scenarios, we used additional annotations and enrichment analyses to identify the potential direct regulators. Other scenarios include genetic variants that change the structure of the TF and thereby its binding affinity and genetic variants that affect sub-cell-type composition and thus the correlation pattern of sub-cell-type-specific genes.

We then replicated the identified co-eQTLs in a large bulk study (BIOS Consortium), explored technical factors influencing the identification of co-eQTLs (sample size, number of cells, different filtering approaches) and biologically interpreted several examples of co-eQTLs (Fig. 1d).

### Correlation validation

Co-expression correlations can be assessed using various dependency measures. A recent benchmark study [16] reported that the proportionality measure from the propr package [15] outperforms several other methods in the identification of functional, coherent biological clusters. We observed high correlations between the rho proportionality and Spearman correlations (*r* = 0.68) for genes expressed in > 5% of the cells (Additional file 2: Fig. S2a), but for genes expressed in fewer cells, rho proportionality gave arbitrarily high values while the Spearman correlation remains near zero (Additional file 2: Fig. S2b). The reason for the stark differences for very lowly expressed genes is probably that rho proportionality changes zero values to the next lowest value of the gene pair, which may result in false positive associations (i.e., very high rho values) for lowly expressed gene pairs. Another drawback of rho proportionality is the high computational demand [24], which makes it challenging to evaluate all gene pairs. As the differences between the Spearman correlation and rho proportionality are very small for highly expressed genes and the Spearman correlation calculation is far more efficient and handles zero values better, we chose to use the Spearman correlation over rho proportionality.

We also tested other approaches, including GRNBoost2 [21], grouping cells into *MetaCells* [17] before calculation of the Spearman correlation, and testing pseudotime ordering [25] and RNA velocity [26], but these did not yield more reliable results than the Spearman correlation (Additional file 2: Fig. S3,4,5; Additional file 3). We therefore selected the Spearman correlation to measure the co-expression patterns in scRNA-seq data for its robustness and simple interpretability. However, although we determined that the Spearman correlation was optimal for the single-cell PBMC datasets that we studied, we cannot exclude that the other methods might be optimal for other single-cell datasets.

We then evaluated whether the co-expression patterns obtained from scRNA-seq data are robust and reproducible across different single-cell datasets and whether they reflect functional interactions among genes. Benchmarking the co-expression patterns obtained from scRNA-seq data is difficult because, to our knowledge, there is no clear gold standard dataset of known functional gene–gene interactions for different cell types. As an alternative approach to assess the reliability of the identified co-expression relationships, we compared to what extent we could replicate the co-expression patterns found in one dataset in another dataset.

**Fig. 2:**
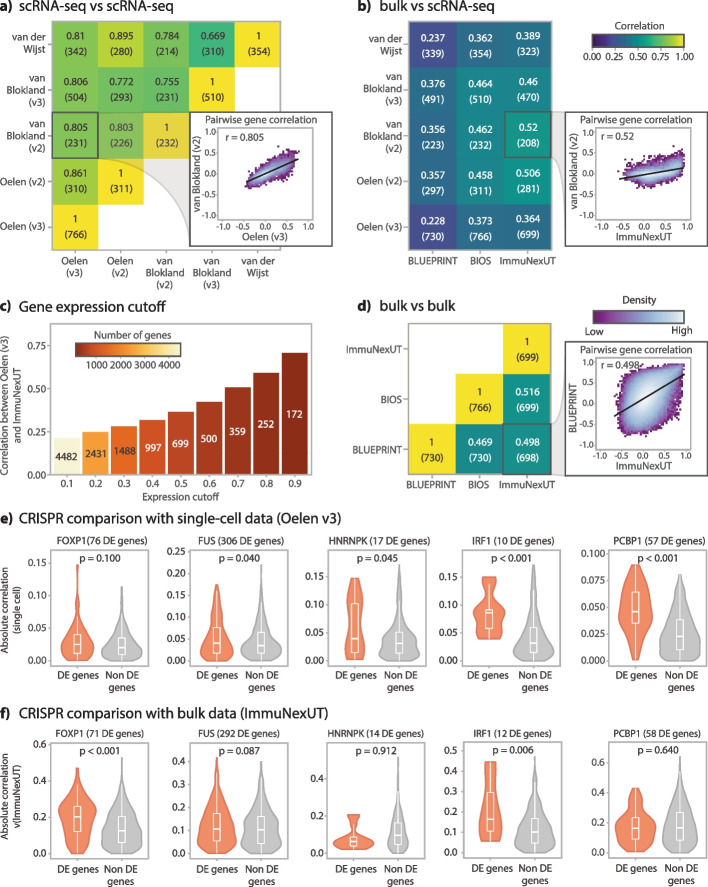
Evaluation of correlation metrics.** a** Comparison of the co-expression profiles among the different single-cell datasets in this study. The Spearman correlation of the Oelen v2 and v3 datasets, the van Blokland v2 and v3 datasets and the van der Wijst dataset were compared with each other, always taking the CD4 + T cells and genes expressed in at least 50% of the cells in the corresponding datasets. The number of genes tested is shown in parentheses below the exact Spearman correlation value.** b** Comparison of the co-expression profiles between the single-cell datasets and with the bulk RNA-seq datasets from BLUEPRINT, ImmuNexUT (both measuring FACS-sorted naive CD4 + T cells), and BIOS (whole blood). Again, we only assessed genes expressed in at least 50% of the cells for the single-cell dataset (number of tested genes shown in parentheses below the Spearman correlation value).** c** Relationship between the co-expression similarity between the ImmuNexUT naive CD4 + T cells and Oelen v3 dataset CD4 + T cells and increasing gene expression cutoffs (the ratio of cells with non-zero expression for a given gene). The number of genes tested is indicated by color scale and the numbers in the bar plot.** d** Comparison of the co-expression profiles between the bulk RNA-seq datasets, taking the same gene subset as in **a** and **b**. The number of tested genes is shown in parentheses below the exact Spearman correlation value.** e** Enrichment of correlated genes in scRNA-seq (Oelen v3 dataset) among associated genes identified by CRISPR knockout. For the enrichment, genes differentially expressed after knockout of FOXP1, FUS, HNRNPK, IRF1, and PCBP1 were identified and tested for enrichment. *P*-values in the plot show the significance level of the Wilcoxon rank-sum test.** f** Enrichment of correlated genes in bulk RNA-seq (ImmuNexUT) among associated genes identified by CRISPR knockout. See **e** and “Methods” for further details

We first compared the cell-type-specific co-expression patterns among the five scRNA-seq datasets in our study [10, 11, 20]. For this, we inferred the co-expression strength using the Spearman correlation for each gene pair in each dataset and cell type, where gene pairs were only considered when both genes were expressed in at least 50% of the cells. We summarized the concordance between datasets by calculating the Pearson correlation on the gene pair correlation values. Overall, there was high concordance across all cell types (median *r* = 0.80 across all cell types). CD4 + T cells, the most abundant cell type in our dataset, had a high correlation across the different 10X chemistries and datasets, with values ranging from 0.67 to 0.86 and a median of 0.81 (Fig. 2a). For CD8 + T cells and NK cells, we observed a comparably high correlation (CD8 + T cells median *r* = 0.86, NK cells median *r* = 0.80), while the correlation was slightly lower for the other cell types (monocytes median *r* = 0.69, B cells median *r* = 0.70, DCs median *r* = 0.71) (Additional file 2: Fig. S6). The number of genes expressed in 50% of the cells varied between dataset and chemistry, so it was not always possible to test the same set of genes. In general, this filtering strategy is quite stringent, yielding a limited number of tested genes (at most 766 genes for the Oelen v3 dataset in CD4 + T cells, Fig. 2a). This ensured a high-quality gene set remained for testing despite the sparseness of the complete single-cell datasets. A detailed evaluation of the expression cutoff follows in the next sections.

Next, we compared the co-expression patterns from the single-cell datasets to three different bulk datasets from BLUEPRINT [22], ImmuNexUT [23], and the BIOS Consortium [2]. The BLUEPRINT dataset contains fluorescence-activated cell sorting (FACS)-sorted expression data from naive CD4 + T cells and classical monocytes for up to 197 individuals. The ImmuNexUT study collected gene expression data from 337 patients for 28 FACS-sorted immune cell subsets. The BIOS dataset contains whole-blood expression data from 3198 individuals. Notably, the co-expression correlation between the single-cell and bulk-based datasets (Fig. 2b) was much lower than those between the single-cell datasets themselves (Fig. 2a).

Comparing our single-cell data with ImmuNexUT, the only dataset with cell-type-specific expression for all evaluated cell types, CD8 + T cells showed the highest correlation (median *r* = 0.570) and monocytes (median *r* = 0.395) and DCs (median *r* = 0.259) showed the lowest correlations (Fig. 2b, Additional file 2: Fig. S7). The correlations from BLUEPRINT were slightly lower but in the same range (CD4 + T cells median *r* = 0.356, monocytes median *r* = 0.339) (Fig. 2b, Additional file 2: Fig. S7). Finally, we observed that the whole-blood bulk data from the BIOS dataset correlated reasonably with the different single-cell cell types (median *r* between 0.265 and 0.458 across cell types; Fig. 2b, Additional file 2: Fig. S7).

We studied this seemingly low correlation between bulk and single-cell data and identified multiple factors that play a role. One is the sparseness of the single-cell data, which could introduce noise and therefore lead to less stable co-expression values. To test this, we correlated the co-expression from the Oelen v3 dataset with that from ImmuNexUT using varying expression cutoffs based on the number of cells expressing a gene (Fig. 2c). Indeed, the sparseness of the single-cell data affects the correlation. We observed increased concordance with increasing gene expression levels: the correlation increased from *r* = 0.21 for an expression cutoff of 10% to *r* = 0.71 at a cutoff of 90%. However, the number of genes that can be tested dropped from 4482 at an expression cutoff of 10% to 172 at a cutoff of 90%. The same trends were observed when comparing the Oelen v3 dataset with the BLUEPRINT dataset for different cutoffs (Additional file 2: Fig. S8). For this reason, we chose a cutoff of 50% as a trade-off between both extremes in our benchmarking study (Fig. 2a,b,e,f).

Other aspects that may affect correlations between genes are the difference in resolution and potential biases introduced by acquiring cell-type-specific data, such as the gene expression changes induced by FACS and the technical complications of deconvoluting cell types. Furthermore, the validity of bulk-based correlations is affected by the possibility of Simpson’s paradox [27] occurring. Simpson’s paradox describes the incorrect introduction or removal of correlations by averaging expression levels. This can potentially occur in bulk datasets, whereas single-cell data can accurately identify the co-expression value since we can calculate co-expression values per cell type and per individual (Additional file 2: Fig. S9a). To estimate the effects of this phenomenon, we recalculated co-expression from the single-cell data using the so-called pseudo-bulk approach, where we average the counts over all cells per individual and calculated the correlation across the individual-level counts. We compared this to the single-cell co-expression values, calculated across cells, and observed several examples of highly expressed genes in which Simpson’s paradox occurs (Additional file 2: Fig. S9b, c). However, taking the average gene expression over many cells also results in more robust expression estimates, which can generate less noisy co-expression estimates, especially for lowly expressed genes. For this reason, we cannot differentiate for all genes which co-expression differences between single-cell and bulk are caused by Simpson’s paradox and which are caused by noisy single-cell data.

To contextualize the correlation values between single-cell and bulk data, we also compared the bulk datasets with each other and assessed whether bulk datasets actually capture gene co-expression consistently. Surprisingly, the co-expression correlation similarity between bulk datasets was quite low (*r* between 0.47 and 0.52 for CD4 + T cells and between 0.35 and 0.42 for monocytes) (Fig. 2d, Additional file 2: Fig. S10). Given that these correlations are expected to be an upper bound when comparing bulk datasets with single-cell datasets, our observed correlations in those comparisons are very reasonable.

Given the imperfect correlation between the different bulk datasets, we used gene expression data from CRISPR-knockouts as an additional evaluation criterion. For this purpose, we benchmarked the co-expression patterns from our single-cell datasets against a CRISPR knockout scRNA-seq dataset in CD4 + T cells [19]. While a unique single-guide RNA barcode reveals which gene was targeted in which cell, some cells may escape from successful CRISPR perturbation. To account for this, we used Mixscape to assign a perturbation status to each cell [28]. For each knockout, we then determined other genes that were differentially expressed (DE) in successfully perturbed cells compared to wild-type cells. We then selected genes for which perturbation resulted in at least 10 DE genes and compared the correlation of these DE genes with non-DE genes using the Wilcoxon rank-sum test (see “Methods”). For four out of five gene knockouts, we observed significantly higher correlation of the knockout gene with the DE genes than with non-DE genes (*p* < 0.05) in the single-cell dataset (Fig. 2e). In contrast, the bulk naive CD4 + T cell data from ImmunNexUT showed a weaker connection between correlation and DE genes, with only two out of five knockout genes having significantly higher correlation with the DE genes (*p* < 0.05) (Fig. 2f).

As another line of evidence, we tested whether pairs of genes known to interact on the protein level showed higher co-expression correlation compared to other pairs of genes. Here we found that gene pairs with protein interactions listed in the STRING database [29] had a higher co-expression correlation than gene pairs not in STRING, both when using the single-cell dataset and the bulk dataset (for both Wilcoxon rank-sum test, *p* < 0.05, Additional file 2: Fig. S11).

Overall, we have shown that single-cell data can identify true gene co-expression relationships as supported by the high replicability of the scRNA-seq-derived co-expression patterns among different datasets and which are also supported by functional interactions among genes identified by CRISPR perturbations and the STRING database.

### Cell type and donor differences in co-expression pattern

**Fig. 3:**
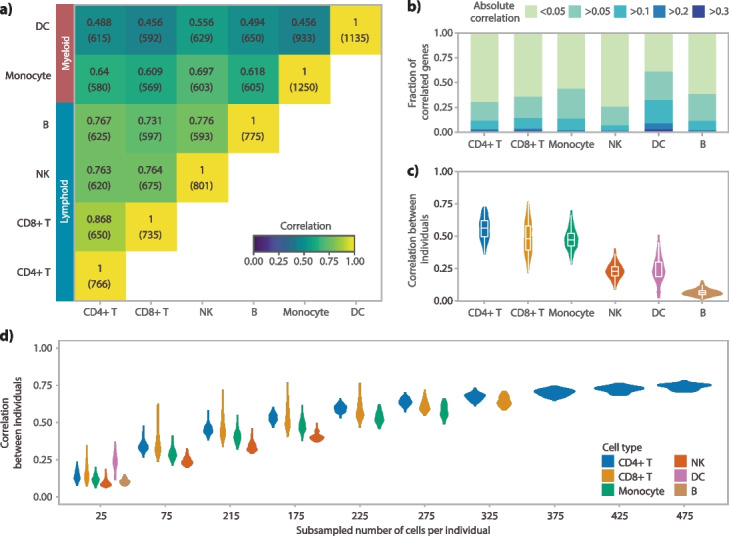
Comparison of correlation across cell types and donors. Each analysis was performed in the Oelen v3 dataset for all genes expressed in at least 50% of the cells of the respective cell type.** a** Comparing co-expression patterns across cell types within the Oelen v3 dataset for genes expressed in 50% of the cells for both cell types in each pair-wise comparison. The number of tested genes is shown in parenthesis below the Spearman correlation value. **b** Correlation distribution within each cell type. **c** Correlation between different individuals within each cell type showing the distribution of all pair-wise comparisons between individuals. **d** Relationship between the number of cells per individual and cell type and correlation between individuals separately for each cell type. In each subsampling step, we assessed all individuals who have at least this number of cells and subsampled to exactly this number (this leads to removal of some individuals for higher number of cells and thus, a direct comparison with the correlation values in **c** is not possible)

Next, we examined cell-type-specific and individualized co-expression patterns. As expected, lymphoid cell types (B, T and NK cells, *r* > 0.73) were more alike with each other but they are less alike with myeloid cell types (monocytes and DCs, *r* > 0.45) (Fig. 3a, Additional file 2: Fig. S12a). However, myeloid cell types were not as alike to each other as lymphoid cell types. This is possibly due to the fact that DCs are one of the least abundant cell types (Additional file 2: Fig. S1), which would have resulted in less accurate co-expression estimations. Overall, the correlation between different cell types within one scRNA-seq dataset (for Oelen v3 dataset median *r* = 0.64, Fig. 3a) was generally lower than the correlation between different scRNA-seq datasets when studying a single cell type (median *r* = 0.80 across all cell types, Fig. 2a, Additional file 2: Fig. S6). These differences highlight cell-type-specific differences in the correlation pattern, further confirming the biological aspects captured by scRNA-seq co-expression values. We also explored the distribution of co-expression among cell types (Fig. 3b, Additional file 2: Fig. S12b). Typically, the correlations between gene pairs were rather low, with only a small proportion of gene pairs (median 12.4%) showing correlations above 0.1. However, we did observe cell-type-specific differences, with DCs possessing a higher proportion of co-expressed gene pairs compared to the other cell types (32.3% of gene pairs with *r* > 0.1).

In addition to detecting cell-type-specific associations, scRNA-seq enables direct calculation of co-expression correlations per individual as it provides many observations (cells) per donor. When we calculated the correlation separately for each donor and cell type, we observed overall strong correspondence of co-expression networks between different donors for the more frequent cell types (CD4 + T cells median *r* = 0.56, CD8 + T cells median *r* = 0.48, monocytes median *r* = 0.47) (Fig. 3c, Additional file 2: Fig. S12c). As a result of noisier estimates, the correlation between individuals was drastically lower for the less frequent cell types (DCs median *r* = 0.24, B cells median *r* = 0.06). Moreover, these correlations were much smaller than comparing one cell type across entire datasets (i.e., including all individuals at once), which showed correlations of at least 0.81 for CD4 + T cells, 0.64 for CD8 + T cells, 0.49 for monocytes, 0.66 for NK cells, 0.62 for B cells, and 0.38 for DCs (Fig. 2a). This decline is potentially caused by the number of cells used to calculate the correlation, which is drastically lower when comparing donors within one dataset. The number of cells could also explain the differences between the cell types. To test this, we subsampled the number of cells for each cell type and indeed observed that the correlation increased when the number of cells increased (Fig. 3d). Apart from the number of cells, we also observed potential cell-type differences. The similarities between individuals were significantly smaller in NK cells compared to monocytes and T cells, when the same number of cells was used (Fig. 3d). We also confirmed these observations in another scRNA-seq dataset (Additional file 2: Fig. S12d).

We further explored the relationship between the number of cells per individual and the correlation between individuals by fitting a logarithmic curve for the four most frequent cell types: CD4 + T cells, CD8 + T cells, monocytes, and NK cells (Additional file 2: Fig. S13). Each of the observed trends could be fit well with the logarithmic curve (adjusted *R*^2^ values between 0.86 and 0.98). We then extrapolated the trend to 1000 cells, showing that a correlation > 0.80 would be expected for T cells and monocytes with this number of cells and a correlation of 0.65 for NK cells (Additional file 2: Fig. S13). We acknowledge, however, that the exact upper bound for the correlation between donors cannot be estimated accurately with our current dataset. For example, the correlation close to 100% for CD4 + T cells and 1500 cells is likely too high considering that donor-specific differences such as genetics and environment will remain independent of the number of cells. Nevertheless, our fits highlight the value of having measurements from many cells for accurate correlation estimates as well as cell-type-specific differences in the correlation pattern.

During this comparison, we observed a few gene pairs that showed a high variance in correlation across donors within one cell type (median fraction of gene pairs with correlation *Z*-score variance > 2 across cell types: 4.9% for Oelen v2 dataset and 3.3% for Oelen v3 dataset, Additional file 2: Fig. S14). This high variance could, in theory, be caused by different sources, e.g., technical factors or environmental influences, but could also reflect genetic differences between individuals. Since we observed low co-expression variance between different individuals for the same cell type and similar numbers of cells (Fig. 3d), we concluded that these differences are not likely to originate from technical factors, and thus we next looked into genetic variation as one of the other potential major influences.

### Establishing a method to identify co-expression QTLs

To assess how strongly genetic variation influences the correlation between pairs of genes, we performed a co-eQTL analysis. In contrast to classical eQTL analysis, co-eQTL analysis not only reveals the downstream target gene whose expression is affected by a genetic variant, it can also help identify the upstream regulatory factors that affect these eQTLs, as discussed in the overview.

Compared to an eQTL analysis, a full co-eQTL analysis with all SNP–gene pair combinations would massively increase the multiple testing burden. Previously, we showed the necessity of filtering the SNP–gene pair combinations to reduce the multiple testing burden associated with a genome-wide co-eQTL analysis on all possible triplets while not missing true co-eQTLs [11]. For example, in our current study, testing all pairs of genes expressed in monocytes would lead to 1.96 × 10^8^ tests when considering only one SNP per pair and to a very limited power to detect small effect sizes (power of 1.4% to detect a significant effect for a phenotype (here the co-expression relationship) with a heritability of 10% that is explained by a single locus, Additional file 2: Fig. S15).

In this study, we aimed to define a generally applicable co-eQTL mapping strategy that yields a large number of highly confident co-eQTLs, which, compared to our previous studies, represents the following: (1) a rigorous assessment and comparison of different analysis strategies, (2) an updated gene and gene pair filtering strategy, (3) an improved computational pipeline for better handling the missing values, and (4) a customized stricter multiple testing strategy. For the updated filtering strategy, we first decided to focus on *cis*-eQTL SNPs and genes because we expect a SNP influencing the co-expression of two genes to also influence the expression of one of the genes directly (a strategy we applied successfully before in [10, 11]). To identify these *cis-*eQTLs, we first performed a *cis*-eQTL meta-analysis across four of the five scRNA-seq datasets. We excluded the van Blokland v3 dataset from this eQTL analysis and all subsequent analyses because the small sample size (*N* = 14) provided very few variants above the minor allele frequency (MAF) cutoff (> 10%), which made it unsuitable for this meta-analysis. To reduce the multiple testing burden and maximize the number of *cis*-eQTLs detected given the relatively low number of individuals (*N* = 173) used for the eQTL mapping, we confined ourselves to 16,987 lead *cis*-eQTLs previously identified in a large (*N* = 31,684) bulk blood eQTL study [2]. Depending on cell type, we identified between 904 (for CD4 + T cells) and 58 (for B cells) eQTLs (FDR < 0.05; Additional file 1: Table S2, Additional file 4: Table S3).

As filtering for the eQTL effects still resulted in a large number of tests (e.g., for CD4 + T cells, n = 12,137,281, Additional file 1: Table S4) and consequently a large multiple testing burden, we imposed additional filtering on the co-eGenes to study. Here, we used a filtering strategy based on the co-expression significance, selecting co-eGenes for which we observed a significant (nominal *p* ≤ 0.05) correlation with the eGene in at least 10% of the individuals (“Methods”). We assumed this captures genuine co-expression effects that are present in at least one of the genotype groups (i.e., homozygous reference/heterozygous/homozygous alternative allele). Note that the filtering strategy we used here is less stringent than the cutoff used in the co-expression benchmarking analyses (Figs. 2 and 3; “Methods”). This is because the two analyses have very different goals, while the benchmarking was more technical in nature, we aimed to uncover new biology in the co-eQTL analyses. Thus we used a less stringent selection in the co-eQTL analysis to ensure that we did not miss out on detecting relevant biological processes underlying gene regulation.

An additional challenge is the large number of missing co-expression values for gene pairs within individuals. This is introduced by the sparsity of the scRNA-seq data: correlation is missing when the expression of one gene is zero in all cells of an individual. We argue that these missing co-expression values may not reflect true null correlations between gene pairs because zero values in single-cell data can also be caused by lowly expressed genes not being quantified accurately. As we observed that replacing missing values with 0 can lead to spurious co-eQTL results (Additional file 2: Fig. S16), we remove the missing correlations and do not impute the missing correlation to 0 when mapping co-eQTLs.

Finally, we applied a customized permutation strategy for each gene pair. Since common upstream regulators might lead to co-expression of many co-eGenes, we expect correlated test statistics among the family of tests carried out for each SNP–eGene pair. Therefore, we applied a customized permutation and multiple testing correction strategy per SNP–eGene pair based on FastQTL [30, 31]. We used 100 permutations to adjust nominal *p*-values and determined significance using Benjamini–Hochberg correction over all SNP–eGene pairs. We considered those with FDR ≤ 0.05 as significant (see “Methods” for details).

### Meta-analysis identified 948 co-eQTLs

With our co-eQTL mapping strategy, we conducted a meta-analysis with four of the five single-cell datasets (Oelen v2 and v3, van Blokland v2 and the van der Wijst dataset). This identified cell-type-specific co-eQTLs for 72 independent SNPs, affecting 946 unique gene pairs in total (Table 1, Additional file 1: Table S5, Additional file 5: Table S6). We identified the maximum number of 500 co-eQTLs in CD4 + T cells, comprising 30 SNPs, 500 gene pairs, and 420 unique genes. We identified the minimum number of 35 co-eQTLs in B cells, comprising 1 SNP, 35 gene pairs, and 36 unique genes.

**Table 1:** Summary statistics of the identified co-eQTLs

Cell type	# co-eQTLs	# unique co-eQTL SNPs	# unique co-eQTL gene pairs	# unique co-eQTL genes	# tests
CD4 + T	500	30	500	420	179,841
CD8 + T	420	22	420	322	73,017
Monocyte	281	24	280	235	304,707
DC	58	9	58	62	41,655
NK	123	10	123	121	25,998
B	35	1	35	36	2,936

**Fig. 4:**
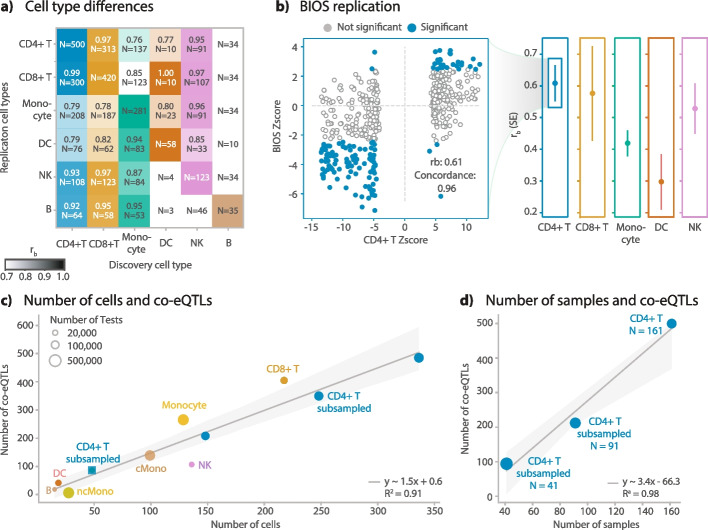
General characteristics of identified co-eQTLs.** a** Replication of discovered co-eQTLs across the major cell types. Correlation of the effect sizes in replications among different cell types, measured by *r*_*b*_ value. Text inside each block indicates the *r*_*b*_ value, and number of replicated co-eQTLs. Color intensity indicates *r*_*b*_ value. For certain cell-type combinations, the number of co-eQTLs were too few to reliably estimate *r*_*b*_ values. For those cell types, only the number of co-eQTLs is shown. **b** Replication in BIOS dataset for different cell types, indicated by the *r*_*b*_ values. Scatter plot shows the detailed *Z*-score comparison between the co-eQTL meta-analysis and the *Z*-score from the BIOS replication for CD4 + T cells. **c** Number of significant co-eQTLs for varying cell numbers. Dot color indicates the cell type, as indicated in the text next to each dot. “cMono” means classical monocytes. “ncMono” means non-classical monocytes. “CD4 + T Subsampled cells” means that this analysis was done for CD4 + T cells, but for every individual we randomly downsampled cells to the desired cell number as indicated in the *x*-axis. **d** Number of significant co-eQTLs for varying sample numbers. “CD4 + T Subsampled Individuals” indicates that this analysis was done for CD4 + T cells, but we randomly subsampled for the individuals

We first examined the cell-type specificity of these co-eQTLs. This analysis is limited by the fact that, due to our filtering strategy, we used a different set of cell-type-specific eQTLs and tested a different set of co-eGenes. Consequently, this resulted in very different sets of tested triplets for biologically different cell types, which could explain the small overlap of significant co-eQTLs between cell types (Additional file 2: Fig. S17, 18a; Additional file 5: Table S6). Therefore, to give a complete picture of the cell-type specificity of co-eQTLs, we replicated co-eQTLs from each cell type in all other cell types and quantified this with two different measures: (1) the ratio of co-eQTLs that could be tested in the replication cell type (Additional file 2: Fig. S18a) and (2) the *r*_*b*_ concordance measure [32], which reflects the correlation of the effect sizes for the co-eQTLs that were tested in the replication cell type (Fig. 4a, Additional file 2: Fig. S18b, details in “Methods”). Consistent with the co-eQTL overlap results, the ratio of tested co-eQTLs are generally small, with the median value being 37% (Additional file 2: Fig. S18a). However, for the SNP–eGene–co-eGene triplets that were tested in the replication cell type, their effect sizes and directions were generally highly concordant, with a median *r*_*b*_ value of 0.85 (Fig. 4a, Additional file 2: Fig. S18b, Additional file 1: Table S7). The highest *r*_*b*_ were observed between CD4 + T cells and CD8 + T cells (0.97 for co-eQTLs identified in CD4 + T cells replicated in CD8 + T cells, 0.99 for co-eQTLs identified in CD8 + T cells replicated in CD4 + T cells).

To validate our co-eQTL results, we first examined the effect sizes and directions among the datasets used in the meta-analysis and observed high correlations (Additional file 2: Fig. S19). Next, we replicated them in the BIOS bulk whole-blood dataset (*N* = 2491 excluding common individuals, see “Methods”) (2), using the ratio of tested co-eQTLs and *r*_*b*_ value (see “Methods”). For this replication, we used a linear regression model with an interaction term to model the associations between the expression level of eGenes and the product of genotype and the expression level of co-eGenes (see “Methods” for detailed explanation), as we have done before [8]. We tested all identified co-eQTLs in the BIOS data and their effect sizes and directions showed *r*_*b*_ values between 0.30 to 0.61 (Fig. 4b, Additional file 1: Table S8, Additional file 6: Table S9), with the highest concordance achieved for CD4 + T cells, with an *r*_*b*_ value of 0.61 (SE = 0.06). We only considered BIOS, rather than the BLUEPRINT and ImmuNexUT, as a replication dataset because BIOS has 2491 individuals while the other two only have a few hundred individuals.

After we established a baseline for the number of co-eQTLs identified and their replication rates, we used this to evaluate various technical factors such as the filtering strategy, sub-cell-type composition, sample size, and cell number. We first compared the analysis to a set of co-eQTLs identified when omitting the filtering step for significantly correlated gene pairs, which increased the number of tests (Additional file 1: Table S4). While this led to detection of an increased number of co-eQTLs for the more abundant cell types (CD4 + T, CD8 + T, monocytes, and NK cells) and a decreased number of co-eQTLs for less abundant cell types (B cells and DCs) (Additional file 1: Table S4, Additional file 7: Table S10), we also observed a general decrease in concordance among cell types compared to the co-eQTLs obtained with the filtering strategy (Additional file 2: Fig. S18, 20; Additional file 1: Table S11). We then repeated the BIOS replication procedures for co-eQTLs found without the filtering strategy and observed a decrease in effect concordance compared to the set of co-eQTLs identified with the filtering strategy (Additional file 2: Fig. 21–23; Additional file 1: Table S12, Additional file 8: Table S13), indicating that the filtering increases the robustness of the co-eQTLs.

We additionally explored the correlation mean and variance, as well as the non-zero ratio for co-eQTLs compared to non-significant triplets, in the scenarios with and without additional filtering (Additional file 2: Fig. S24). Here we observed that significant co-eQTLs show both a higher co-expression correlation mean and variance and a higher non-zero ratio for their expression (Additional file 2: Fig. S24) compared to non-significant triplets. This is to be expected as gene pairs with a high average co-expression correlation more likely reflect true biological associations and gene pairs with a high correlation variance likely reflect true co-expression network polymorphisms. This trend is also much clearer for the filtered set compared to the non-filtered set (Additional file 2: Fig. S24), suggesting that alternative preselection strategies could be envisioned that are based on specific expression values or co-expression correlation variance thresholds.

Sub-cell-type composition is a potential confounder that might introduce false positive co-eQTLs, similar to cell-type composition in bulk studies [33]. If a genetic variant is associated with sub-cell-type composition, co-eQTLs with sub-cell-type-specific genes might be identified even when there is no direct association between the SNP and the co-expression. To assess this, we analyzed co-eQTLs found among classical monocytes, non-classical monocytes, and the whole set of all monocytes. Here we found that co-eQTL effect sizes are highly concordant (*r*_*b*_ ≥ 0.9) (Additional file 2: Fig. S25) for co-eQTLs tested in one of the subtypes and in the major cell type (> 82% of co-eQTL identified in monocytes were tested in both classical monocytes and non-classical monocytes). This suggests that the co-eQTLs are not generally driven by sub-cell-type composition, although individual co-eQTLs could still be caused by sub-cell-type differences.

To highlight how future co-eQTL analyses can benefit from the expected expansion of population-based scRNA-seq datasets with available genotype data, we determined how the number of identified co-eQTLs is related to the number of individuals and cells per individual. To test the influence of the number of cells, we randomly subsampled the CD4 + T cells and monocytes per individual and repeated the co-eQTL mapping pipeline (Fig. 4c). For the influence of the number of individuals, we randomly subsampled the individuals for CD4 + T cells (Fig. 4d). We observed that the number of co-eQTLs is linearly and positively correlated with both the number of cells and the number of individuals, although the number of individuals had a stronger effect than the number of cells (Fig. 4c, d; Additional file 1: Table S5).

### Annotating identified co-expression QTLs

After we successfully validated the identified co-eQTLs by exploring different technical aspects and replicating them in the BIOS dataset [2], we examined to what extent the co-eQTLs could provide interesting biological insights into genetic regulation, which could be relevant for the interpretation of disease variants. As discussed in the overview, we hypothesize that among the co-eGenes identified for each SNP–eGene pair there are direct regulator genes or genes co-expressed with the direct regulators for the eGene. Even if the direct upstream regulatory factor was not evaluated in the co-eQTL analysis, due to the limited capturing efficiency of the single-cell data, the biological function of the co-eQTLs could still be inferred by the other co-eGenes in strong co-expression with the unknown upstream regulator as they presumably share the same biological function and potentially also a common role in disease. To assess these hypotheses, we combined different lines of evidence: functional enrichment based on gene ontology (GO) terms, enrichment of TF binding sites and enrichment of GWAS annotations.

Each enrichment analysis was run separately per cell type and for all co-eGenes associated with the same SNP–eGene pair (see “Methods” for details). To increase the power of enrichment analyses, we restricted ourselves to SNP–eGenes pairs with at least five co-eGenes, which covered 25% of SNP–eGenes pairs in at least one cell type (19 out of 76 unique SNP–eGene pairs). GO enrichment analysis revealed shared functional pathways for the co-eGenes. For 18 of the 19 SNP–eGene pairs, we found enrichment among the associated co-eGenes for at least one GO term (Additional file 9: Table S14). Moreover, we assessed potential common TFs regulating the shared function of these co-eGenes using ChIP-seq data processed by ReMap 2022 [34] and found enrichment of TF binding sites in the promoter regions of co-eGenes for 7 of the 19 SNP–eGene pairs (Additional file 10: Table S15). For four of the SNP–eGene pairs, the co-eQTL SNP itself or a SNP in high linkage disequilibrium (LD) (*R*^2^ ≥ 0.9) lay in the binding region of the enriched TFs (Additional file 10: Table S15), making these likely candidates for the direct regulator.

We also explored whether co-eQTLs and the respective sets of co-eGenes could enhance our understanding of disease-associated variants. For this, we annotated co-eQTL SNPs with GWAS loci, identifying approximately half the SNPs to be in high LD (*R*^2^ ≥ 0.8) with a GWAS locus (41 out of 72 SNPs, Additional file 11: Table S16). To assess if sets of co-eGenes for a specific SNP–eGene share a common role in disease, we explored if the co-eGenes show higher gene-level trait association for GWAS traits that are also associated with the respective co-eQTL SNP. We identified overlapping GWAS traits for two co-eQTL SNPs and their co-eGenes for at least one GWAS trait and cell type, with many of the traits covering blood cell counts and immune-mediated diseases (GWAS SNP *p*-value < 5 × 10^−8^, FDR < 0.05, Additional file 12: Table S17), further strengthening the biological connection of the co-eGenes with the eQTL.

Furthermore, we observed that the direction of effect of the co-eQTLs can be helpful in grouping genes sharing the same functions. For this, we compared the direction of effect of the co-eQTL with the direction of the associated eQTL, choosing the same reference allele in both cases. If the direction matched, we classified it as concordant. In these co-eQTLs, increasing expression of the eGene led to increasing co-expression. If the directions did not match, we said the direction of the co-eQTL is discordant. Between 37 and 97% of the co-eQTLs showed a concordant direction of effect across cell types (Additional file 2: Fig. S26), but the majority of co-eGenes were associated with rs1131017–*RPS26* and thus the observed distributions are probably not generalizable for future larger studies that identify more co-eQTL.

In the following section, we highlight some examples of how these co-eQTL can help to better understand the molecular functional consequences of genetic variants associated with disease. To gain additional support for the biological interpretation of these co-eQTLs, we performed a colocalization analysis overlaying the eQTL and co-eQTL signals with the GWAS signals of the most important traits from the enrichment analysis (Additional file 13: Table S18, Additional file 14: Table S19).

**Fig. 5:**
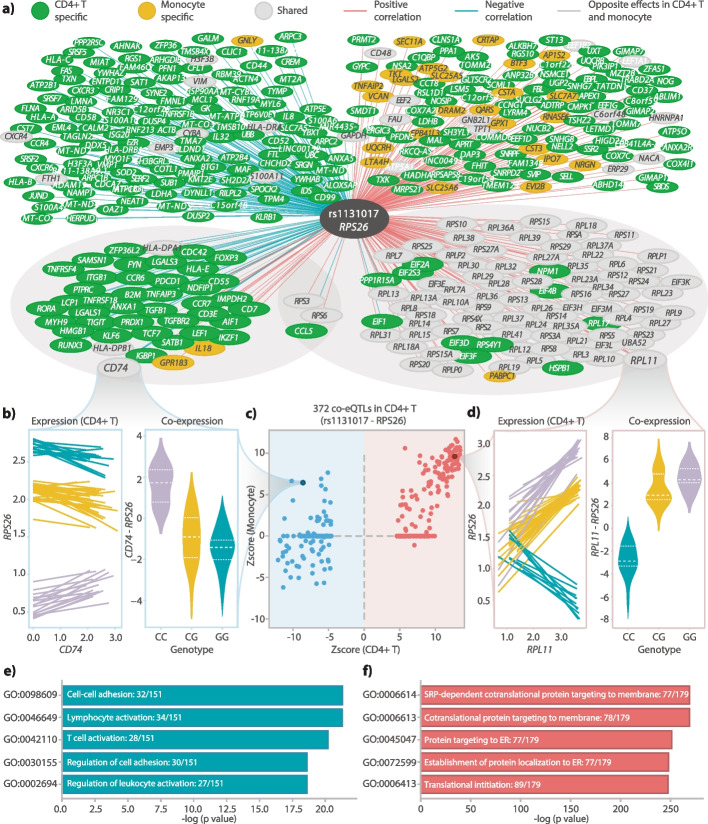
Annotation of co-eQTLs.** a** Union network constructed with co-eQTLs found in CD4 + T cells or monocytes that are associated with the SNP–eGene: rs1131017–RPS26. The two circled clusters contain co-eGenes that are in those corresponding GO terms. **b** Example of one co-eQTL: rs1131017–RPS26-CD74. Left plot indicates the co-expression patterns from all individuals in the Oelen v3 dataset. Each regression line was fitted using the normalized gene counts resulting from the “SCTransform” workflow [35], from one individual. Right plot indicates the co-expression values from the three genotype groups. **c** Comparison between *Z*-scores from monocytes and Z-scores from CD4 + T cells. Red dots indicate positive co-eQTLs from CD4 + T cells. Blue dots indicate negative co-eQTLs from CD4 + T cells. **d** Example of one co-eQTL: rs1131017–RPS26-RPL11 with the same layout as **b**. **e** GO term enrichment results for the co-eGenes in negative co-eQTLs from CD4 + T cells (top five GO terms for the Ontology Biological Process). **f** GO term enrichment results for the co-eGenes in positive co-eQTLs from CD4 + T cells (top five GO terms for the Ontology Biological Process)

When grouping co-eQTLs based on their associated eQTL, eQTL rs1131017–*RPS26* had the most significantly associated co-eGenes in all cell types except for DCs (between 372 co-eGenes for CD4 + T cells and 35 for B cells) (Fig. 5a–d). *RPS26*, encoding a ribosomal protein, showed strong correlation with other ribosomal proteins, and we had previously reported a few *RPS26* co-eQTLs in CD4 + T cells [10] and monocytes [11]. Our new methodology and the larger sample size in the current study allowed us to now compare the genes part of the rs1131017–*RPS26* co-eQTLs across cell types.

In monocytes, NK cells and B cells, nearly all the associated genes showed a positive direction of effect, concordant with the eQTL direction (95% of all co-eGenes for monocytes, 90% for NK cells and 97% for B cells), while in CD4 + T cells and CD8 + T cells, several genes showed a negative direction of effect, discordant with the eQTL direction (46% of all co-eGenes for CD4 + T cells and 43% for CD8 + T cells).

The positively associated genes replicated well across all cell types (Fig. 5c, Additional file 2: Fig. S27) and were enriched for functions associated with translation (Fig. 5f), which is consistent with the fact that many co-eGenes were ribosomal proteins from both the large and the small subunit (for CD4 + T cells: 46 of 47 tested RPL genes and all 31 tested RPS genes were associated). In contrast, the negatively associated genes only replicated well between CD4 + T cells and CD8 + T cells (Additional file 2: Fig. S27; Fig. 5c, d), despite the fact that these genes were sufficiently high expressed in the other cell types. This negatively associated set of genes showed enrichment in functions associated with immune response and T cell activation (Fig. 5e).

TF enrichment analysis identified six TFs—*RBM39, TCF7, LEF1, KLF6, CD74,* and *MAF*—whose binding sites were enriched in the promoter region of the rs1131017–*RPS26* co-eGenes, that had a binding site overlapping with rs1131017 and that were among the rs1131017–*RPS26* co-eGenes themselves (Additional file 10: Table S15). This led us to the assumption that one or more of these TFs represent the direct regulators of the eQTL, as described in the overview (Fig. 1c, Scenario 1). Five of the TFs (*TCF7, LEF1, KLF6, CD74,* and *MAF*) are also connected with lymphocyte activity (the first four based on GO annotations, *MAF* based on a recent study [36]), further strengthening the link with T cell activation. Of these, *MAF* and *CD74* were specifically enriched not only among all co-eGenes but additionally among co-eGenes with a negative effect direction (Additional file 10: Table S15).

GWAS enrichment analysis showed enrichment for several different blood cell counts in all cell types. However, in CD4 + T cells and CD8 + T cells, we additionally observed specific enrichment for the immune-mediated diseases (rheumatoid arthritis (RA), Crohn’s disease (CD), multiple sclerosis (MS) and hay fever). This shows the relevance of T cell-associated co-eGenes for these diseases (Additional file 12: Table S17). We confirmed with colocalization analyses that there is very likely a shared signal between the eQTL and co-eQTLs signals and the GWAS signals from RA and asthma (Additional file 13: Table S18, Additional file 14: Table S19). Among the co-Genes with the highest colocalization posterior probability (PP4 > 0.9), 6 out of 41 were again associated with lymphocyte activation. Interestingly, several studies have highlighted a connection of RPS26 with T cell activation and survival [37], and the associated co-eQTL SNP rs1131017 is associated with the enriched immune-mediated diseases (RA, CD, MS, hay fever) [38].

We examined whether the large number of co-eQTLs for rs1131017 were confounded by sub-cell types in CD4 + T cells. We cannot exclude the possibility that this variant showed this effect in CD4 + and CD8 + T cells by specifically affecting the amount of circulating CD4 + or CD8 + sub-cell types whose marker genes would subsequently show up as co-eQTLs in our analysis, where we have not distinguished between sub-cell types. To test whether this is a possibility, we associated SNP rs1131017 and the ratio between CD4 + /CD8 + TEM cells and CD4 + /CD8 + naïve T cells, but we did not see a significant correlation (Additional file 2: Fig. S28). Together, these results suggest that RPS26 plays a dual-function role, both in general transcription and specifically in lymphocytes in T cell activation. This points to a potential working mechanism in the role of rs1131017 in the manifestation of autoimmune diseases.

Another set of promising co-eQTLs are those associated with rs7806458–*TMEM176A* in monocytes (11 co-eGenes) and rs7806458–*TMEM176B* in monocytes (6 co-eGenes) and DCs (1 co-eGene) as they connect the co-eQTL SNP rs7806458 that has been associated with MS [39] with blood coagulation. This is interesting as this disease has previously been connected to disturbances in blood coagulation [40]. The relevance of the co-eGenes to MS is supported by two lines of evidence. Firstly, GO enrichment suggested that the six co-eGenes associated with rs7806458–*TMEM176B* in monocytes are enriched for complement component C3b binding (Additional file 9: Table S14), which is closely related to the blood coagulation system [41]. When looking closely at the exact gene functions, we found three genes (*ITGB1, FCN1,* and *CFP*) that contribute to the local production of complement [42]. Secondly, GWAS enrichment analysis showed MS enrichment for co-eGenes associated with rs7806458–*TMEM176A* in monocytes (Additional file 12: Table S17). Intriguingly, the eGene *TMEM176B* was previously found to be associated with the maturation of DCs [43], and it has been shown that white blood cells, including DCs, can act as a local source of certain complement proteins [44, 45]. Though we could not identify (in)direct regulator genes for these co-eQTL in our TF enrichment analysis with the ReMap database (version 2022) [34], we argue that these co-eGenes, supported by several lines of evidence, provide valuable mechanistic insights for the MS SNP rs7806458.

For several of the other co-eQTLs, we could not provide as strong and coherent evidence for the interpretation but nevertheless found promising connections to biological functions and disease that can be explored in further studies. One is the SNP–eGene pair rs9271520–*HLA-DQA2*. We found co-eQTL effects for it in CD4 + and CD8 + T cells, monocytes, and DCs, with the number of co-eGenes ranging from 7 to 17. Interestingly, rs9271520 is in LD with several immune disease SNPs where we also found enrichment for the co-eGenes in the same GWAS traits. The most significant (sorted by GWAS SNP *p*-values) enriched traits include rheumatoid arthritis, MS, and asthma (see Additional file 12: Table S17 for full GWAS enrichment results). The connection with these three diseases was also supported in our colocalization analysis, indicating colocalization in various cell types (29 colocalizing co-eGenes for asthma, 21 for RA, and 2 for MS; PP4 > 0.5, Additional file 13: Table S18, Additional file 14: Table S19). However, we found several other genes in the HLA region being co-eGenes associated with rs9271520–*HLA-DQA2*, and, when we removed those HLA genes, the GWAS enrichment signals disappeared. This indicated that the enriched signal could be due to the LD structure in the HLA region and a confident mapping of the causal regulatory connections is not possible with our dataset. Other interesting co-eQTL examples and their interpretations are discussed in Additional file 3.

In general, our study is still underpowered in finding a lot of associated co-eGenes (Fig. 4c, d, Additional file 2: Fig. S15). This limits the set of SNP–eGenes, for which we can perform a well powered enrichment analysis and so the biological interpretation of these co-eQTLs. One of the potentially interesting SNP–eGenes, with too few co-eGenes for the enrichment analysis, is rs393727—*RNASET2*, which is associated with four co-eGenes (*B2M, ITGB1, ALOX5AP, CRIP1*)*.* The SNP rs393727 is in very high LD with two previously described SNPs associated with Crohn’s disease (CD) and inflammatory bowel disease (IBD) (Additional file 11: Table S16). Additionally, the results of our colocalization analysis support a shared signal between the eQTL rs393727—RNASET2 in CD4 + T cells and GWAS signals for IBD (including Crohn’s disease and ulcerative colitis cases together) and more specifically only CD (Additional file 13: Table S18). For the co-eQTL colocalization, the posterior probabilities for a shared signal were lower (around 0.5), which could be caused by a lack of power in our current analysis (Additional file 14: Table S19). The eGene *RNASET2* has also been previously associated with IBD [46], and among the four co-eGenes, *ITGB1* was previously associated with CD [47] and *CRIP1* is associated with gut immunity [48], further supporting the link of these co-eQTL genes with IBD and CD.

Intriguingly, we found a number of overlapping co-eGenes associated with different SNP–eGene pairs, indicating potential common upstream regulatory pathways. For example, all the co-eGenes positively associated with rs4147638–*SMDT1* are also found to be positively associated with rs11311017–*RPS26*, while the four co-eGenes negatively associated with rs393727–*RNASET2* are also negatively associated with rs1131017–*RPS26* (Additional file 2: Fig. S29).

## Discussion

In this study, we validated the use of scRNA-seq data to identify cell-type-specific co-expression patterns and developed a novel approach to extend the discovery of co-eQTLs. Applying this to a large meta-analysis with 173 samples, we identified 72 independent SNPs leading to co-eQTLs for 946 unique gene pairs across different cell types. These co-eQTLs shed light on the biological processes upstream of individual *cis*-eQTLs, such as that seen for rs1131017, which affects *RPS26* expression levels and is associated to autoimmune diseases. We observed that this variant affects T cell activation genes, providing a potential explanation for the association of this variant to autoimmune diseases.

In this study, we used the Spearman correlation to quantify the co-expression patterns from scRNA-seq data because of its straightforward interpretability, scalability, robustness against outliers, and high reproducibility among different scRNA-seq and bulk RNA-seq datasets. However, we acknowledge that such correlations do not take into account the sparseness of scRNA-seq data, and it is difficult to infer direct regulator genes. This of course also depends on the quality of the single-cell data. Direct interactions can only be distinguished from indirect interactions when the direct upstream target was measured, which is currently not always the case. We tested other association methods [15, 21], including the proportionality measure and GRNBoost2, that were recognized as top-performing in independent benchmarking studies [16, 18]. However, they did not perform better in our validation. Additionally, a reliable temporal ordering of the cells [25, 26] was not possible in our dataset. We therefore applied the Spearman correlation as a solid basis for the co-eQTL analysis. However, we do acknowledge that the Spearman correlation may not be the ideal method to handle scRNA-seq data due to sparseness. Future work may find that other association measures are equally suitable or more suitable, and this may potentially depend on the specific single-cell dataset under investigation.

We also found that scRNA-seq and bulk RNA-seq data do not always correlate well for all gene pairs and explored different factors that could explain this. Part of the variable correlation could be explained by the sparsity of the single-cell data, as higher expressed gene pairs correlated better, but at least a few example cases showed the potential occurrence of Simpson’s paradox. With regard to cell-type composition, however, the FACS-sorted datasets did not correlate better with single-cell datasets than the whole-blood bulk dataset, which could either be caused by the smaller sample size of the single-cell data, technical changes introduced by FACS or specific differences in the (sub-)cell types, as we had naïve CD4 + T cells and classical monocytes (subsets of CD4 + T cells and monocytes, respectively) for BLUEPRINT and ImmuNexUT that we tested for the single-cell data. Another interpretation is that scRNA-seq and bulk RNA-seq data capture different functional gene clusters, as a previous study showed in tumor samples [49]. One possible explanation for this is that bulk and single-cell capture different sources of variability. Whereas single-cell data captures between-cell variability, bulk data captures between-person variability, which is affected by additional factors like genetics and environment. Therefore, a statistical framework combining both data types could be beneficial in the future.

Our study sheds light on several important considerations for future scRNA-seq study design regarding personalized network construction and co-eQTL mapping. Firstly, we showed that several factors, including cell number and gene selection, greatly influence the stability of co-expression patterns. We observed a clear trend indicating that a certain minimum number of cells from one individual is needed to achieve a stable co-expression pattern (Fig. 3d). Secondly, we also explored factors influencing the number and quality of co-eQTLs. We showed that the number of significantly detected co-eQTLs can be greatly increased by either increasing the number of individuals or by increasing the number of cells per individual (Fig. 4c, d). The limited power is also visible in the number of identified single cell eQTLs, which is well in line with other single-cell studies of similar sample size [11], but lower compared to large bulk eQTL studies [2]. We believe that future larger single-cell datasets such as two very recent studies [50, 51] and the sc-eQTLGen consortium [52] will improve statistical power to identify more robust eQTLs and co-eQTLs.

Furthermore, we showed that a sophisticated filtering strategy of tested SNP–gene–gene triplets is essential to maximize the number of reliable co-eQTLs. However, we also suggest that the filtering strategy should be designed for the specific goals of the respective analysis. In this study, we systematically searched for robust co-eQTLs and adapted our strategy to balance the trade-off between achieving a stable co-expression pattern and enlarging the search space. For this reason, we first selected SNP–gene pairs and then used co-expression strength as an additional criterion rather than the very stringent expression cutoff criterion we used in our benchmarking analysis. In contrast, in our previous study [11], we focused specifically on co-eQTLs among the eQTLs that changed after pathogen stimulation and performed a strict pre-filtering for a highly targeted analysis. In the current study, we were, in particular, able to replicate the most significant co-eQTLs from the targeted analysis (Additional file 2: Fig. S30). While the targeted analysis identified additional lower significance co-eQTLs that are below our much stricter multiple testing-corrected significance threshold, we were able to quantify the number of co-eQTLs more broadly for several additional SNPs and to include, for the first time, a comparison across cell types. In other cases, a selection of known TF–target pairs or pathway information could be desirable, e.g., for prioritizing TFs connected with diseases for experimental validation purposes.

Additionally, we investigated if there is an eQTL effect between SNP and co-eGene from SNP–eGene–co-eGene triplets. As a result, we found this to be the case for just two out of 946 distinct co-eQTL triplets, thus we may infer that generally the co-eQTL effect is not directly controlled by the cis-eQTL SNP. In these two specific situations, the co-eGene and eGene were located close to each other and their regulatory SNP.

We showed that the annotated co-eQTLs could identify potential direct regulators of the associated eQTLs as well as the affected biological processes, with several examples based on a combination of different enrichment analyses. We identified several TFs either directly as co-eGenes or via enriched binding sites among the co-eGenes of a SNP–eGene pair, providing potential regulatory mechanisms for explaining the co-eQTL. For the eQTL rs1131017–*RPS26*, six enriched TFs were themselves co-eGenes in CD4 + T cells, providing compelling evidence to support the hypotheses that direct regulators can be identified among co-eQTLs. Among these six TFs, five are associated with lymphocyte activation, further strengthening the connection of the eQTL with lymphocyte activation and through this to autoimmune diseases.

Another interesting aspect of the rs1131017–RPS26 example is that we revealed a potential mechanism for a previously described GWAS signal by showing cell-type-specific genetic regulation of a multi-functional gene. The SNP rs1131017 is in high LD with rs773125 (*R*^2^ = 0.879), which has previously been associated with rheumatoid arthritis in several large-scale GWAS studies [53, 54]. A recent TWAS study [55] utilized the GWAS summary statistics [53] and *RPS26* was identified as one of the significant genes in the locus. Additionally, the SNP rs1131017 was found as the leading SNP for a trans-eQTL locus specifically in T cells [56]. Inspired by these observations, a recent paper [37] sought to elucidate the role of RPS26 in T lymphocytes. They examined a T-cell-specific RPS26 knockout mouse model and reported that ablation of RPS26 in T cells impairs peripheral T cell homeostasis and leads to T cell developmental arrest in the thymus. Despite the great interest in this locus and the role of RPS26 in lymphocytes, the associated pathways and biological processes that underlie the rheumatoid arthritis GWAS signal are still largely unknown. By comparing T cells and monocytes, we identified that RPS26 may be involved in two distinct biological functions. Interestingly, these two distinct functional co-eQTL clusters are characterized by opposite effect directions. Moreover, while RPS26 showed enough variation to be picked up as an eQTL effect, it did not show high correlation with either gene cluster (Additional file 2: Fig. S31), which may be why understanding its role in multiple functions has been challenging up to now [37]. We envision that more multi-functional eGenes could operate in such a cell-type-specific manner, with variation in expression that could be explained as the downstream consequences of many other conserved or highly co-expressed gene clusters, and this understanding could assist in interpreting GWAS signals. We also observed that different eGenes could have shared upstream genes/pathways as we identified four common immune-related co-eGenes associated with rs393727–RNASET2 and rs1131017–RPS26, and both SNPs were in LD with immune diseases (T1D and CD), suggesting a shared upstream process for these two eQTL effects. By providing cell-type-specific gene regulation backgrounds through co-eQTLs, we expect more eQTLs and GWAS signals to be explained in the relevant cell type via future large-scale co-eQTL studies.

The choice of the background set for the enrichment analyses affects the interpretation. We decided to include all genes tested for co-eGenes in the respective cell type into the background gene set. We also explored the effect of a more specific background, which focused on the genes tested for the respective SNP–eGene in the cell type (Additional file 1: Table S20). The total number of enriched pairs was reduced slightly with the more specific background, but the enrichment results for rs1131017-RPS26 and our other examples in the results section stayed very similar and the changes did not affect our interpretation.

For other co-eQTL examples, no enriched TF was found in the co-eGene list, potentially because the TFs were not measured in the scRNA-seq datasets due to low expression. By also doing an enrichment analysis for TFs regulating the set of co-eGenes, we did identify a set of candidate TFs which are likely candidates for further investigation as potential regulators. A third group of co-eQTL examples were supported by GWAS or GO enrichment analysis but not TF enrichment analysis. Here, the co-eGenes revealed part of the disease-relevant network, but we could not pinpoint the direct regulatory TFs. One explanation for this may be that our study is still underpowered to discover co-eGenes, while the enrichment strategy works best when there are a substantial number of co-eGenes as for rs1131017–*RPS26*. Based on our evaluation, we estimated that future studies with larger sample size and more cells will identify many more co-eQTLs (Fig. 4c,d). This can help identify the direct regulators for some of our other examples, where the current enrichment analyses provided no clear interpretation, as well as co-eQTLs associated with other SNP–eGenes.

There are also several challenges to interpret the identified co-eQTLs. Firstly, as discussed earlier, it is difficult to determine the direct and indirect regulators that work through co-expression among correlated co-eGenes. This creates problems in using correlation-based metrics to quantify replication performance. For example, all the co-eQTLs we identified in B cells were associated with the rs1131017–RPS26 pair, making the correlation-based *r*_*b*_ measure invalid for this case. Also, to reduce the multiple testing burden, we only tested the top-SNP, a choice that could pose additional challenges for follow-up analysis such as colocalization to identify the causal SNP. Moreover, comparison of co-eQTLs between cell types remains challenging. We showed that the number of co-eQTLs is strongly driven by the number of cells (Fig. 4c), so that it is not meaningful to only compare the absolute number of co-eQTLs between cell types in the current study. Furthermore, the sparsity of the single-cell data lead to the removal of many lowly expressed genes which, combined with the strict filtering our analysis required, meant only a small number of genes were tested in all cell types.

Several confounding factors such as sequencing depth, number of cells and sub-cell-type composition could influence the results. In this study, we used SCT normalization for the scRNA-seq data to reduce the impact of sequencing depth on downstream analysis. We normalized the individualized co-expression values by the number of cells per individual and calculated *z*-statistics (see “Methods”) for co-eQTL mapping. In addition, fine grained compositional differences within a cell-type can introduce false positive co-eQTLs within a cell type if a genetic variant influences this composition and one of the tested genes shows differences in expression that also depend on this composition. However, in our evaluation of classical and non-classical monocytes, we observed no strong confounding of monocyte co-eQTLs by the subtypes of cells (Additional file 2: Fig. S24). Although we cannot fully rule out all other possible confounding factors that could potentially create false positive findings, we have shown that the identified co-eQTLs show highly consistent effect sizes and directions across all datasets used in our meta-analysis and also in the independent bulk replication cohort. This indicates that the majority of our reported co-eQTLs are not due to batch effects. However, we do encourage future studies to further examine potential confounding factors to further reduce the possibilities of false positives and false negatives.

Several of the limitations of our current analysis will be overcome by on-going technological developments. First of all, we expect that follow-up analyses with larger sample sizes and more cells per person will identify many additional co-eQTLs. This can be further enhanced by improvements in single-cell technologies that lead to better capture efficiency of expressed genes. CITE-seq [57] and similar technologies [58, 59] allow improved cell type and sub-cell-type classification that can show the effect of sub-cell-type differences more accurately. The combination of multiple-omics, such as scRNA-seq, scATAC-seq, and/or single-cell proteomics [60–62], will enable us to capture regulation happening outside the mRNA level and lead to improved association analysis of gene pairs above the standard Spearman correlation.

## Conclusion

Through our co-eQTL mapping strategy we identified a robust set of co-eQTLs that provides insight into cell-type-specific gene regulation and leads for future functional testing. Among these results, we uncovered a potential mechanism for a previously identified GWAS signal and a multi-functional gene. Our evaluation of different technical factors provides valuable suggestions for future experimental study design. We believe that more co-eQTLs will be uncovered by applying our general co-eQTL mapping pipeline to future large-scale scRNA-seq data. We envision that these co-eQTLs will in the future help to position eQTL and GWAS signals into cell-type-specific GRNs by annotating which regulatory edges are affected by which genetic variants. This knowledge is important for interpreting the effects of genetic variants in general, but also specifically for improve personalized medicine through better genetic risk prediction for diseases and personalized drug treatment based on genotype [52].

## Methods

### Single-cell datasets

Three different scRNA-seq datasets were included in this study, both for benchmarking the associations and for combined meta-analysis of co-expression QTLs. All five datasets from the three studies were generated from human PBMCs and are referred to by their first author: the Oelen dataset (*n* = 104 donors) [11], the van der Wijst dataset (*n* = 45 donors) [10], and the van Blokland dataset (*n* = 38 cardiac patients) [20]. Further specifications can be found in Additional file 1: Table S1a) and 1b), and the respective manuscripts. Briefly, the Oelen dataset and the van der Wijst dataset included general population participants with European ancestry background, whose ages range from 20 to 79. The van Blokland dataset included patients 6–8 weeks post ST-elevated myocardial infarction, whose ages range from 43 to 95, the majority of whom are of European ancestry background. In the van Blokland data, there are two individuals of Asian ancestry background. We examined how much the meta-analysis *Z*-scores of the identified eQTLs and co-eQTLs would be changed by excluding the two individuals, but did not observe a large discrepancy (Additional file 2: Fig. S32, 33).

For each study, blood samples were collected from individuals into EDTA-vacutainers (BD), after which PBMCs were isolated using Cell Preparation Tubes with sodium heparin (BD) and were cryopreserved in RPMI1640 containing 40% FCS and 10% DMSO until further use. For each individual, cryopreserved PBMCs were thawed and 6–8 donors were multiplexed in individual sample batches that were then loaded on a 10X Chromium controller. Subsequent libraries were then generated for each sample batch using 10 × Genomics single cell 3’ reagents (v2 and v3). Libraries were sequenced using 150PE sequencing on the Illumina NovaSeq 6000 at BGI (Hong Kong).

For dataset processing, we used CellRanger (v1.3 for van der Wijst and v3.0.2 for Oelen and van Blokland) for scRNA-seq alignment, Demuxlet for demultiplexing, and Seurat (4.1.0) for quality control (QC) and Azimuth v0.4.6 (with the default reference from [63]) for cell-type classification. Cells with a high percentage of mitochondrial genes (5% in the van der Wijst study, 8% and 15% in v2 and v3 chemistries respectively in both Oelen and van Blokland studies), number of genes expressed per cell (more than 3500 genes/cell in van der Wijst study and less than 200 genes/cell in Oelen and van Blokland studies), and doublets (Demuxlet for van der Wijst and SoupOrCell for Oelen and van Blokland) were excluded during these QC steps. The Oelen dataset also contains cells stimulated with different pathogens, but we only included the unstimulated cells in this analysis to improve comparison with the other datasets. For the van Blokland dataset, we included the data from the time point 6–8 weeks after the individual was admitted to the hospital for myocardial infarction, again to improve comparison across datasets. More details on dataset processing are provided in the original publications [10, 11, 20].

For cell-type classification, we took the annotation for the Oelen data from their original publication [11] and annotated the van Blokland and van der Wijst datasets using the Azimuth classification method [63]. For Azimuth classification, we used the following settings: (1) the FindTransferAnchors function to find anchors using the reference from publication [63], normalization method “SCT”, reference.reduction method “spca” and first 50 dimensions and (2) the MapQuery function to annotate cell types using the same reference and parameters such as reference.reduction = “spca” and reduction.model = “wnn.umap”. We then compared the annotation from the Oelen publication and the Azimuth classification and found high correspondence (Additional file 2: Fig. S34). For analyses using the sub-cell-type classification, we always refer to the Azimuth classification results.

### Single-cell co-expression

For co-expression quantification, we used SCTransform [35] from the Seurat package and used the log transformed counts as input. We then calculated the Spearman correlation of gene pairs in the three different single-cell studies (Oelen dataset [11], van Blokland dataset [20] and van der Wijst dataset [10]) and then compared between datasets and 10XGenomics chemistry. In the benchmarking section, correlation was calculated separately per cell type but together over all individuals and only for gene pairs for which both genes were expressed in at least 50% of the cells from the respective cell type. The correct gene counts resulting from the “SCTransform” [35] were used to calculate gene–gene correlations. For the comparison between two datasets, the gene pair-wise Spearman correlation values from each dataset were compared using the Pearson correlation.

### Rho calculation

Rho proportionality was calculated using the “propr” function in R, from the “propr” package, with the symmetrized value set to true. We used the v3 unstimulated monocytes to compare the rho proportionality values to the Spearman-rank correlations of the same data. We filtered out genes expressed in fewer than 5% of cells, leaving 8634 genes to be assessed. Concordance between the rho values and Spearman correlations was assessed with the Pearson correlation.

We also explored rho proportionality values for very lowly expressed genes because the log-normalization of the method potentially introduces false associations for these genes [15]. However, the computational demand to run the method was so high that we could not evaluate all expressed genes at once. Instead, we subsampled a set of 50 very lowly expressed genes (expressed in 0–5% of the cells) and 50 very highly expressed genes (expressed in at least 90% of the cells) and calculated the rho proportionality and Spearman correlation values for each combination of these 100 genes. We then compared gene pairs for which both genes were lowly expressed, pairs for which both genes were highly expressed, and mixed pairs, for which one gene was lowly and one highly expressed.

Alternative association metrics besides the rho proportionality and Spearman correlation are discussed in Additional file 3.

### Validation in bulk datasets

The Spearman correlations from single-cell data were compared to the Spearman correlations made with three different bulk datasets: the BLUEPRINT Epigenome consortium data [22], the ImmuNexUT dataset [23], and the BIOS dataset [2]. For BLUEPRINT, we further removed the first principal component from the monocyte dataset to remove any uncorrected covariates. For the ImmuNexUT dataset, preprocessing was performed as described in the publication: we filtered out genes with less than 10 counts in 90% of the samples, performed TMM normalization with edgeR and scaling to CPM, batch corrected with combat, and removed samples with a mean correlation coefficient smaller than 0.9. For the BIOS dataset, we corrected for 20 RNA Alignment metrics and then calculated the co-expression values using all individuals.

We then calculated the Pearson correlation across all gene pair-wise correlation values. As BLUEPRINT and ImmuNexUT are cell type-sorted datasets, we matched the cell types between bulk and single-cell data in these cases in the comparison. Again, we used only genes expressed in at least 50% of the cells from the cell type. This threshold was chosen after our initial evaluation of different thresholds from 10 to 90% in the comparison of BLUEPRINT and Oelen v3 dataset, with 50% chosen to balance the number of genes that can be used against the correlation strength between the datasets.

### Validation using CRISPR knockout data

To further validate the correlation values, we used CRISPR knockout data from [19]. Mixscape was used to identify perturbed vs unperturbed cells for each CRISPR perturbation [28]. We selected five knockout genes for which a sufficient number of successful CRISPR-perturbed cells were identified and that were expressed in our single-cell dataset (Oelen v3 dataset, CD4 + T cells) in > 50% of cells. The publication identified DE genes in wild-type vs perturbed cells and wild-type vs non-perturbed cells, as labeled by Mixscape. We selected a credible set of DE genes that were expressed in the single-cell dataset and significant in the wild-type vs perturbed cells but not in the wild-type vs non-perturbed cells. For this, we applied FDR-correction based on all genes expressed in the single-cell dataset. The correlation of these genes was compared to the correlation of non-DE genes, i.e., all other genes expressed in the single-cell dataset, using the Wilcoxon rank-sum test (one-sided test with “greater” in DE genes). The same test was done using the naive CD4 + T cells from the ImmuNexUT dataset.

### Validation using STRING annotations

Following the same approach used for the CRISPR knockout data, we explored if gene pairs whose proteins are interacting show higher correlation. We used the STRING database (version 11) [28], processed by the [18] benchmark study, to identify interacting gene pairs. We compared the correlation of gene pairs in STRING versus gene pairs not listed in STRING via Wilcoxon rank-sum test (one-sided test with “greater” for Gene pairs in STRING): once using the correlation estimates from the Oelen v3 dataset and once using the estimates from the ImmuNexUT dataset, both times for the CD4 + T cells and filtered for genes expressed in > 50% of single cells.

### Exploring Simpson’s paradox

To identify whether our strategy to identify single-cell co-expression is affected by Simpson’s paradox and whether bulk-based approaches would suffer from it, we studied the co-expression outcomes for two different strategies. In both strategies, we only included genes with non-zero expression in at least half of all monocytes in the Oelen v3 dataset. In the first strategy, we calculated the Spearman correlations for gene pairs per individual separately for each gene pair, based in the corrected gene counts resulting from the “SCTransform” workflow [35]. In the second strategy, we calculated the average expression of genes per individual, called pseudo-bulk approach and then calculated the Spearman correlation between genes. To identify potential Simpson’s paradox events, we looked into the gene pairs that had the largest deviation in co-expression estimate between the two strategies.

### Comparison between cell types

After successful validation of the Spearman correlation values, we compared differences between cell types within one dataset for the Oelen v2 and v3 dataset. Here we applied the same strategy as in the dataset comparison. We selected genes expressed in 50% of the cells from both cell types for each corresponding comparison, calculated the Spearman correlation per gene pair within each cell type, and followed up with the Pearson correlation to compare both cell types. We also explored the absolute distribution of correlation coefficients between the cell types.

### Comparison between individuals

Again, we applied the same strategy as for the cell type and dataset comparison. We calculated gene pair-wise Spearman correlation values for each cell type and donor separately, taking all genes expressed in 50% of cells from the cell type in general (not per donor). We then compared each donor with each other donor by calculating the Pearson correlation over the gene pair-wise correlation values to get a distribution of how well donors match per cell type.

To explore the effect of the number of cells per donor on this distribution, we subsampled each cell type to different numbers of cells (depending on the frequency of the cell type). For this, we take all individuals with at least this number of cells in this cell type and subsample the cell number to exactly this value for each individual. We stop subsampling at a threshold for the cell type when more than 75% of all measured individuals have fewer cells than the threshold. For the four most abundant cell types (CD4 + T cells, CD8 + T cells, monocytes and NK cells), we additionally fitted a logarithmic curve separately for each cell type to better quantify the connection:

$$\mathrm{correlation}\_\mathrm{individuals }\sim \mathrm{ log}(\mathrm{number}\_\mathrm{cells}) (\mathrm{with log being the natural logarithm})$$

We then used the fitted formulas to extrapolate up to 1500 cells for each cell type.

### Power calculation

For power calculation, we use an F-test, as implemented in [61], with a sample size of 173 (the total size of the combined cohorts), a heritability between 10 and 30% and a Bonferroni-corrected significance threshold of 0.05. The range for the heritability was chosen based on previously detected co-eQTLs [11]. The number of tests influences the Bonferroni-corrected thresholds and depends on the selected gene–gene–SNP triplets. Here we assumed only one SNP per gene pair and all genes are tested against each other. Then, we increased the non-zero ratio threshold for gene selection from 0 to 0.95 (monocytes, Oelen v3 dataset), got the number of tests, and calculated the power.

Testing multiple SNPs per pair would further increase the total number of tests and reduce the overall power.

### eQTL mapping

We performed a meta-analysis to identify significant eQTL in four out of the five single-cell datasets (Oelen v2 and v3 dataset [11], van Blokland v2 dataset [20] and van der Wijst dataset [10]) per each major cell type for expressed genes in the corresponding cell type. We excluded the van Blokland v3 dataset because the sample size was so small that few variants lay above the MAF threshold (see below). Due to the limited sample size, we chose to perform a constrained eQTL analysis rather than a genome-wide analysis. We have shown previously [11] that this confined eQTL mapping strategy can greatly reduce the multiple testing burden and identify additional significant eQTLs. In our eQTL meta-analysis, we have increased the number of significant eQTLs from 560 [11] to 615. To select the SNP–gene pair to test for eQTL mapping, we took the eQTL results from the largest eQTL meta-analysis study in whole blood [2] and selected the most significant SNP for each gene. This resulted in 16,987 SNP–gene pairs to test. For these selected SNP–gene pairs, we performed eQTL mapping using eQTLPipeline v1.4.9 [64] within a confinement of top-SNP-Gene from eQTLGen Consortium [2], using 1000 permutation rounds for determining FDR as described in [2] and a MAF of 0.1. The eQTLGen Consortium conducted the cis-eQTL analysis with 1 Mb distance. After filtering for the MAF cutoff in our datasets, 14,009 SNPs remained in total for testing, from initially 16,290 selected SNPs from the eQTLGen Consortium. For each eGene, we first calculated the average of the normalized gene expression level per individual for the eQTL analysis. For each dataset separately, we then calculated a Spearman correlation coefficient for each SNP–eGene. Finally, we performed a sample size weighted meta-analysis over all datasets to determine the meta-analysis *p*-value.

### Co-expression QTL (co-eQTL) mapping and the filtering strategy

First, we generated all possible combinations of the cell-type-specific eQTL findings (denoted as SNP–eGene) from the constrained eQTL mapping procedure in the respective cell type (as explained in the eQTL mapping method section above) and all other genes (denoted as co-eGene) that are expressed in the corresponding cell types. This resulted in the full list of SNP–eGene–co-eGene triplets for co-eQTL mapping analysis. We then calculated co-expression using the Spearman correlation for the unique eGene–co-eGene pairs for each individual using untreated cells of the six major cell types (CD4 + T and CD8 + T cells, monocytes, B cells, NK cells, and DCs) and the sub-cell types in monocytes (classical monocytes and non-classical monocytes). We additionally transformed the Spearman correlation into a *t*-statistics with the sample size being the number of cells in one individual, and subsequently normalized the *t*-statistics to a *z*-statistics that follows a normal distribution with mean of zero and variance of one. For each gene pair, we counted the ratio of individuals who exhibit a significant correlation (nominal *p*-value from the Spearman correlation < 0.05). If at least 10% of individuals showed a significant co-expression correlation for the specific eGene–co-eGene, we took this gene pair further into follow-up analysis. The total number of tests for each cell type can be found in Additional file 1: Table S4.

To assess the impact of cell numbers and sample numbers on the quality and quantity of co-eQTLs, we artificially created a few scenarios with fewer cells per individual and fewer individuals using a random subsampling strategy. To examine the impact of cell numbers, we randomly subsampled the CD4 + T cells per individual to three different levels [50, 150 and 250 cells]. In each level, we kept the individuals with fewer cells, randomly subsampled those with a cell number higher than the corresponding level and performed the co-eQTL analysis using the strategies mentioned. Similarly, to examine the impact of sample numbers, we randomly subsampled 50 and 100 individuals, and excluded nine individuals with fewer than 10 CD4 + T cells for both scenarios.

To map co-eQTLs, we calculated the Spearman correlation coefficients for each selected SNP–eGene–co-eGene triplet within each dataset separately. Similar to the eQTL analysis, we then used a sample size weighted *Z*-score method to perform meta-analysis across included datasets for each triplet, yielding the nominal *p*-value.

### Multiple testing correction strategy for co-eQTL

To account for the correlation structure between co-eQTL SNP–eGene–co-eGene triplets, we modified and applied the permutation-based multiple testing correction strategy from FastQTL [30], implementing the method as follows. For each triplet, we performed 100 permutations. Then, for each SNP–eGene pair, we determined the lowest non-permuted nominal *p*-value and the lowest permuted *p*-value per permutation over all the genes (co-eGene) tested for the SNP–eGene pair, resulting in a list of the 100 lowest permuted *p*-values per SNP–eGene pair. For each SNP–eGene pair, we then fitted a beta-distribution over these 100 permuted lowest *p*-values, which enabled us to subsequently establish the empirical *p*-value for the lowest non-permuted *p*-value. Through this procedure, we ensured that under the null test statistic each SNP–eGene pair has a uniform *p*-value distribution. Finally, over the empirical *p*-values for all SNP–eGene pairs, we calculated Benjamini–Hochberg FDR over the empirical *p*-values. Those SNP–eGene pairs with a FDR ≤ 0.05 were deemed significant.

For each SNP–eGene pair, we also derived a *p*-value cutoff that indicates which of the co-eGenes are significant for that SNP–eGene pair via the following steps. After determining the FDR for all SNP–eGene pairs, we determined the empirical *p*-values that are closest to FDR = 0.05. Using the beta distributions for each SNP–eGene pair, we then determined its nominal *p*-value threshold. All co-eGenes with a nominal *p*-value lower than the corresponding *p*-value threshold for that SNP–eGene pair were considered significant. The nominal *p*-value thresholds range from 1.75e − 41 to 1.34e − 4.

### Replication in BIOS dataset

We replicated the co-eQTL findings in bulk whole-blood RNA-seq data from the BIOS Consortium, using the same method described in a previous study [8]. Briefly, we implemented the following ordinary least squares model with the Python package statsmodels [65]: *eGene* ~ *SNP* + *co-eGene* + *SNP:co-eGene*. We then examined the effect sizes of the interaction term SNP:co-eGene and used Benjamini–Hochberg procedures for multiple testing correction.

### Calculation of rb values and allelic concordance

We used the same evaluation metrics to quantify the cell-type specificity and replication performance in the BIOS data set of the co-eQTLs. First, we used the *r*_*b*_ method with modification. We followed the same procedures as the original study [32] but chose a suggested alternate strategy to estimate errors across gene pairs between two tissues. Whereas the original paper used null SNPs per each eQTL for this purpose, we tested only the significant eQTL SNP for SNP–eGene–co-eGene triplets and therefore we did not have information for the null SNPs. Thus, we used the alternative approach indicated in the original paper with Eq. 1), where *r*_*e*_ is the estimation errors across gene pairs between two tissues, *r*_*p*_ is the correlation of co-expression levels between two cell types in the overlapping sample, *n*_*s*_ is the number of overlapping samples, and *n*_*i*_ and* n*_*j*_ are the number of samples in cell typed *i* and *j*, respectively. For the BIOS replication, we excluded overlapping individuals from the BIOS RNA-seq dataset for the replication analysis. Additionally, in cases where fewer than 10 co-eQTLs were tested in the replication analysis, for example, B cells, we could not get a robust estimation of the *r*_*b*_ value and hence represent them as missing in the “Results” section.

1 $${r}_{e} = {r}_{p}\times \frac{{n}_{s}}{\sqrt{{n}_{i}\times {n}_{j}}}$$

Due to our filtering strategy, we did not always test the same set of SNP–eGene–co-eGene triplets in all cell types. Therefore we also need to compare the tested ratio when quantifying the cell-type specificity. The tested ratio means the SNP–eGene–co-eGene triplets that were also tested in another cell type or the BIOS replication analysis.

A third evaluation metric that we used is allelic concordance between the discovered co-eQTLs and the results in the replication study. This is defined as the ratio of co-eQTLs with concordant effect direction by the number of significant co-eQTLs identified in the replication study.

### Biological interpretation based on enrichment of GO terms, TF binding sites, and GWAS variants

We explored the biological function of the co-eQTLs based on different enrichment analyses that all tested if co-eGenes associated with the same SNP–eGene pair in the same cell type show similar functional properties. For this, we selected all SNP–eGene pairs that had at least five significant co-eGenes in the same cell type.

First, we performed GO enrichment analysis separately for each co-eGene set, grouped by SNP–eGene and cell type, applying the R package clusterProfiler (ver 4.0.5) [66] and performing FDR multiple testing correction separately for each SNP–eGene pair across the different GO terms (defining enrichment below FDR < 0.05 as significant). As the background set for the enrichment, we used all genes tested in the co-eQTL analysis in the respective cell type.

Next, we explored if these co-eGene sets were enriched for certain TF binding sites. TF annotations were taken from ChIP-seq peaks processed in the ReMap 2022 database [34], which we filtered for cell lines associated with blood cell lines. This resulted in a final set of 511 blood-cell-related TFs that were tested. We tested the overlap of these TF peaks with the promoter regions of the co-eGenes tested, defining the promoter region as the region 2kB upstream and downstream of the first transcription start site of the gene. Enrichment was tested based on Fisher’s exact tests for each TF, using all genes tested in the co-eQTL analysis in the respective cell type as the background set. We performed FDR multiple testing correction separately for each SNP–eGene pair over all TFs (defining enrichment below FDR < 0.05 as significant). Furthermore, we explored if the enriched TF itself was a co-eGene associated with the respective SNP–eGene pair and if the co-eQTL SNP or a SNP in high LD (*R*^2^ ≥ 0.9) lies in a binding site of the enriched TF. The SNPs in high LD were obtained from SNiPA [67] using the variant set from the 1000 Genomes Project, Phase 3 v5, European population, Genome assembly GRCH37 and genome annotations from Ensembl 87.

For the GWAS annotations, we considered two different strategies. In the first approach, we annotated SNPs or SNPs in high LD (*R*^2^ ≥ 0.8) with GWAS loci from the GWAS Catalog [1], with the last updated timestamp being 3/1/2022, 07:13 AM (GMT + 0100). LD information for this was taken from LDtrait [68] with the following parameters: window size = 500 KB, reference population = 1000 Genomes CEU, GRCh37. In the second approach, we used the magma method [69] to assess enrichment of GWAS associations among co-eGenes. We obtained uniformly processed GWAS summary statistics for 114 traits that were used for the GWAS analysis of the GTEx consortium [38]. We then followed the strategy previously described by [67]. We defined gene sets for each co-eQTL SNP in each tissue as the set of significant co-eGenes associated with the SNP, as done for the GO and TF enrichment analysis. Protein names/gene symbols were converted to Entrez gene ids and mapped to the corresponding annotations on the human genome assembly 38. We performed individual magma analyses for each trait based on summary statistics and LD structure from the 1000 genomes European reference panel for all gene sets compared to the background set of genes tested for co-eQTL, always conditioning on default gene-level covariates (for example, gene length). Subsequently, we applied the Benjamini–Hochberg method and selected gene set–trait associations with FDR < 5%.

After we observed different distributions of co-eQTLs for rs11311017–*RPS26* with regard to the direction of effect in the different cell types, we repeated all enrichment analysis (GO, TF, and GWAS) separately for the positively associated co-eGenes and negatively associated co-eGenes in CD4 + T cells.

### Biological interpretation based on colocalization analysis

Based on the results of the different enrichment analyses, we selected five loci (RPS26, HLA.DQA, RNASET2, SMDT1, TMEM176A) and seven GWAS traits (T1D, rheumatoid arthritis, CD, MS, asthma, IBD, white blood cell count) for which we decided to further investigate the underlying mechanisms by applying colocalization analysis on the eQTL signal as well as the co-eQTL signal of each co-eGene pair. We tested SNPs surrounding the 1 MB window of the TSS of the eGene for both eQTL and co-eQTL analysis. Then we overlapped those SNPs with the SNPs given in the selected GWAS data of the trait. For all traits except T1D, we used the consolidated GWAS summary statistics of the GTEx consortium [38]. For T1D, we used the GWAS summary statistics reported by Chiou et al. [70]. Then we ran the colocalization analysis separately for each eGene and co-eGene pair in each cell type using the coloc.abf function of the R package Coloc (ver: 5.1.0.1) [71] (setting p12 = 1e − 6, default values for the other parameters).

### Direction of effect

We compared the direction of effect in eQTLs and co-eQTLs by comparing the direction of the *Z*-scores. After ensuring that the reference allele aligns in the eQTL and co-eQTL analysis, co-eQTLs for which the sign of the *Z*-score matches the sign of the eQTL *Z*-score are called concordant. If otherwise, they are called discordant.

## Supplementary Information

**Additional file 1:** **Supplementary Tables.** Collection of small supplementary tables (S1-2,4-5,7-8,11-12) plus all table captions; large supplementary tables are saved as separate csv files in the following.

**Additional file 2:** **Supplementary Figures.** Collection of all Fig. S1-34.

**Additional file 3:** **Supplementary Note.** More extensive description of meta-cell evaluation, alternative GRN construction methods and interpretation of additional co-eQTL examples.

**Additional file 4:** **Table S3.** eQTLs for major cell types.

**Additional file 5:** **Table S6.** The significant co-eQTLs for the major cell types with the filtering strategy

**Additional file 6:** **Table S9.** BIOS replication for filtered co-eQTLs for major cell types.

**Additional file 7:** **Table S10.** The significant co-eQTLs for the major cell types without the filtering strategy

**Additional file 8:** **Table S13.** BIOS replication for unfiltered co-eQTLs for major cell types

**Additional file 9:** **Table S14.** GO enrichment for co-eGenes associated with the same eQTL, for all eQTLs with at least 5 co-eGenes

**Additional file 10:** **Table S15.** TF enrichment of co-eGenes co-eGenes associated with the same eQTL, for all eQTLs with at least 5 co-eGenes, using the Remap 2022 ChIP-seq data

**Additional file 11:** **Table S16.** Annotated co-eQTL SNP with GWAS results

**Additional file 12:** **Table S17.** Enrichment of co-eGenes for GWAS results

**Additional file 13:** **Table S18.** Colocalization results for eQTL signal with GWAS signal

**Additional file 14:** **Table S19.** Colocalization results for co-eQTL signal with GWAS signal

**Additional file 15:** **Table S20.** GO enrichment for co-eGenes associated with the same eQTL, variation of Supplementary Table 14 with a stricter background

**Additional file 16.** Review history.

## Data Availability

All code is available on GitHub at https://github.com/sc-eQTLgen-consortium/li-2023 [72] under BSD-2-Clause license. It is also deposited in a Zenodo repository with https://doi.org/10.5281/zenodo.7623968 [73]. All used single-cell RNA-seq datasets are available online at the European Genome-Phenome Archive: the van der Wijst dataset under the accession number EGAS00001002560 [74], the Oelen dataset under the accession number EGAS00001005376 [75] and the van Blokland dataset under the accession number EGAD00001010064 [76]. Also the BIOS dataset, used for replicating the findings in bulk, is available at the European Genome-Phenome Archive with accession number EGAC00001000277 [77].

## References

[1] Buniello A, MacArthur JAL, Cerezo M, Harris LW, Hayhurst J, Malangone C (2019). The NHGRI-EBI GWAS Catalog of published genome-wide association studies, targeted arrays and summary statistics 2019. Nucleic Acids Res.

[2] Võsa U, Claringbould A, Westra HJ, Bonder MJ, Deelen P, Zeng B (2021). Large-scale cis- and trans-eQTL analyses identify thousands of genetic loci and polygenic scores that regulate blood gene expression. Nat Genet.

[3] van der Wijst MGP, de Vries DH, Brugge H, Westra HJ, Franke L (2018). An integrative approach for building personalized gene regulatory networks for precision medicine. Genome Med.

[4] van Dam S, Võsa U, van der Graaf A, Franke L, de Magalhães JP (2018). Gene co-expression analysis for functional classification and gene–disease predictions. Brief Bioinform.

[5] Langfelder P, Horvath S (2008). WGCNA: an R package for weighted correlation network analysis. BMC Bioinformatics.

[6] Deelen P, van Dam S, Herkert JC, Karjalainen JM, Brugge H, Abbott KM (2019). Improving the diagnostic yield of exome- sequencing by predicting gene–phenotype associations using large-scale gene expression analysis. Nat Commun.

[7] Mordelet F, Vert JP (2008). SIRENE: supervised inference of regulatory networks. Bioinformatics.

[8] Zhernakova DV, Deelen P, Vermaat M, van Iterson M, van Galen M, Arindrarto W (2017). Identification of context-dependent expression quantitative trait loci in whole blood. Nat Genet.

[9] Kim-Hellmuth S, Aguet F, Oliva M, Muñoz-Aguirre M, Kasela S, Wucher V (2020). Cell type–specific genetic regulation of gene expression across human tissues. Science..

[10] van der Wijst MGP, Brugge H, de Vries DH, Deelen P, Swertz MA, LifeLines Cohort Study (2018). Single-cell RNA sequencing identifies celltype-specific cis-eQTLs and co-expression QTLs. Nat Genet..

[11] Oelen R, de Vries DH, Brugge H, Gordon MG, Vochteloo M, single-cell eQTLGen consortium (2022). Single-cell RNA-sequencing of peripheral blood mononuclear cells reveals widespread, context-specific gene expression regulation upon pathogenic exposure. Nat Commun..

[12] Crow M, Paul A, Ballouz S, Huang ZJ, Gillis J (2016). Exploiting single-cell expression to characterize co-expression replicability. Genome Biol.

[13] Vallejos CA, Risso D, Scialdone A, Dudoit S, Marioni JC (2017). Normalizing single-cell RNA sequencing data: challenges and opportunities. Nat Methods.

[14] Kharchenko PV, Silberstein L, Scadden DT (2014). Bayesian approach to single-cell differential expression analysis. Nat Methods.

[15] Quinn TP, Richardson MF, Lovell D, Crowley TM (2017). propr: an R-package for identifying proportionally abundant features using compositional data analysis. Sci Rep.

[16] Skinnider MA, Squair JW, Foster LJ (2019). Evaluating measures of association for single-cell transcriptomics. Nat Methods.

[17] Baran Y, Bercovich A, Sebe-Pedros A, Lubling Y, Giladi A, Chomsky E (2019). MetaCell: analysis of single-cell RNA-seq data using K-nn graph partitions. Genome Biol.

[18] Pratapa A, Jalihal AP, Law JN, Bharadwaj A, Murali TM (2020). Benchmarking algorithms for gene regulatory network inference from single-cell transcriptomic data. Nat Methods.

[19] Gate RE, Kim MC, Lu A, Lee D, Shifrut E, Subramaniam M, et al. Mapping gene regulatory networks of primary CD4 ^+^ T cells using single-cell genomics and genome engineering. Genomics; 2019. [cited 2021 Dec 22]. Available from: http://biorxiv.org/lookup/doi/10.1101/678060.

[20] van Blokland I, Oelen R, de Groot H, van der Harst P, Franke L, van der Wijst MGP. Single-cell dissection of the immune response after a myocardial infarction. manuscript in preparation. 10.1161/CIRCGEN.123.004374 PMC11188632 38752343

[21] Moerman T, Aibar Santos S, Bravo González-Blas C, Simm J, Moreau Y, Aerts J (2019). GRNBoost2 and Arboreto: efficient and scalable inference of gene regulatory networks. Bioinformatics.

[22] Chen L, Ge B, Casale FP, Vasquez L, Kwan T, Garrido-Martín D (2016). Genetic drivers of epigenetic and transcriptional variation in human immune cells. Cell.

[23] Ota M, Nagafuchi Y, Hatano H, Ishigaki K, Terao C, Takeshima Y (2021). Dynamic landscape of immune cell-specific gene regulation in immune-mediated diseases. Cell.

[24] propr: vignettes/b_visualization.Rmd. [cited 2022 Apr 13]. Available from: https://rdrr.io/cran/propr/f/vignettes/b_visualization.Rmd

[25] Cannoodt R, Saelens W, Sichien D, Tavernier S, Janssens S, Guilliams M, et al. SCORPIUS improves trajectory inference and identifies novel modules in dendritic cell development. Bioinformatics; 2016. [cited 2021 Dec 16]. Available from: http://biorxiv.org/lookup/doi/10.1101/079509

[26] Bergen V, Lange M, Peidli S, Wolf FA, Theis FJ (2020). Generalizing RNA velocity to transient cell states through dynamical modeling. Nat Biotechnol.

[27] Simpson EH (1951). The interpretation of interaction in contingency tables. J R Stat Soc Ser B Methodol.

[28] Papalexi E, Mimitou EP, Butler AW, Foster S, Bracken B, Mauck WM (2021). Characterizing the molecular regulation of inhibitory immune checkpoints with multimodal single-cell screens. Nat Genet.

[29] Szklarczyk D, Gable AL, Lyon D, Junge A, Wyder S, Huerta-Cepas J (2019). STRING v11: protein-protein association networks with increased coverage, supporting functional discovery in genome-wide experimental datasets. Nucleic Acids Res.

[30] Ongen H, Buil A, Brown AA, Dermitzakis ET, Delaneau O (2016). Fast and efficient QTL mapper for thousands of molecular phenotypes. Bioinformatics.

[31] Klein N de, Tsai EA, Vochteloo M, Baird D, Huang Y, Chen CY, et al. Brain expression quantitative trait locus and network analysis reveals downstream effects and putative drivers for brain-related diseases. bioRxiv; 2021. p. 2021.03.01.433439. [cited 2022 Feb 25]. Available from: https://www.biorxiv.org/content/10.1101/2021.03.01.433439v2 10.1038/s41588-023-01300-6 PMC10011140 36823318

[32] Qi T, Wu Y, Zeng J, Zhang F, Xue A, Jiang L (2018). Identifying gene targets for brain-related traits using transcriptomic and methylomic data from blood. Nat Commun.

[33] Aguirre-Gamboa R, de Klein N, di Tommaso J, Claringbould A, van der Wijst MG, de Vries D (2020). Deconvolution of bulk blood eQTL effects into immune cell subpopulations. BMC Bioinformatics.

[34] Hammal F, de Langen P, Bergon A, Lopez F, Ballester B (2022). ReMap 2022: a database of Human, Mouse, Drosophila and Arabidopsis regulatory regions from an integrative analysis of DNA-binding sequencing experiments. Nucleic Acids Res.

[35] Hafemeister C, Satija R (2019). Normalization and variance stabilization of single-cell RNA-seq data using regularized negative binomial regression. Genome Biol.

[36] Imbratta C, Hussein H, Andris F, Verdeil G (2020). c-MAF, a Swiss Army Knife for Tolerance in Lymphocytes. Front Immunol.

[37] Chen C, Peng J, Ma S, Ding Y, Huang T, Zhao S (2021). Ribosomal protein S26 serves as a checkpoint of T-cell survival and homeostasis in a p53-dependent manner. Cell Mol Immunol.

[38] Barbeira AN, Bonazzola R, Gamazon ER, Liang Y, Park Y, Kim-Hellmuth S, et al. Exploiting the GTEx resources to decipher the mechanisms at GWAS loci. Genetics; 2019. [cited 2022 Apr 13]. Available from: http://biorxiv.org/lookup/doi/10.1101/814350 10.1186/s13059-020-02252-4 PMC7836161 33499903

[39] Nickles D, Chen HP, Li MM, Khankhanian P, Madireddy L, Caillier SJ (2013). Blood RNA profiling in a large cohort of multiple sclerosis patients and healthy controls. Hum Mol Genet.

[40] La Starza S, Ferraldeschi M, Buscarinu MC, Romano S, Fornasiero A, Mechelli R (2019). Genome-wide multiple sclerosis association data and coagulation. Front Neurol.

[41] Amara U, Rittirsch D, Flierl M, Bruckner U, Klos A, Gebhard F (2008). Interaction between the coagulation and complement system. Adv Exp Med Biol.

[42] Lubbers R, van Essen MF, van Kooten C, Trouw LA (2017). Production of complement components by cells of the immune system. Clin Exp Immunol.

[43] Condamine T, Le Texier L, Howie D, Lavault A, Hill M, Halary F (2010). Tmem176B and Tmem176A are associated with the immature state of dendritic cells. J Leukoc Biol.

[44] Li K, Sacks SH, Zhou W (2007). The relative importance of local and systemic complement production in ischaemia, transplantation and other pathologies. Mol Immunol.

[45] Dixon KO, O’Flynn J, Klar-Mohamad N, Daha MR, van Kooten C (2017). Properdin and factor H production by human dendritic cells modulates their T-cell stimulatory capacity and is regulated by IFN-γ. Eur J Immunol.

[46] Gonsky R, Fleshner P, Deem RL, Biener-Ramanujan E, Li D, Potdar AA (2017). Association of ribonuclease T2 gene polymorphisms with decreased expression and clinical characteristics of severity in Crohn’s disease. Gastroenterology.

[47] Dotan I, Allez M, Danese S, Keir M, Tole S, McBride J (2020). The role of integrins in the pathogenesis of inflammatory bowel disease: approved and investigational anti-integrin therapies. Med Res Rev.

[48] Cai H, Chen J, Liu J, Zeng M, Ming F, Lu Z (2017). CRIP1, a novel immune-related protein, activated by Enterococcus faecalis in porcine gastrointestinal epithelial cells. Gene.

[49] Wang J, Xia S, Arand B, Zhu H, Machiraju R, Huang K (2016). Single-cell co-expression analysis reveals distinct functional modules, co-regulation mechanisms and clinical outcomes. Zhou XJ, editor. PLOS Comput Biol..

[50] Perez RK, Gordon MG, Subramaniam M, Kim MC, Hartoularos GC, Targ S, et al. Single-cell RNA-seq reveals cell type–specific molecular and genetic associations to lupus. Science. 2022;376(6589):eabf1970. 10.1126/science.abf1970 PMC9297655 35389781

[51] Yazar S, Alquicira-Hernandez J, Wing K, Senabouth A, Gordon MG, Andersen S (2022). Single-cell eQTL mapping identifies cell type-specific genetic control of autoimmune disease. Science..

[52] van der Wijst M, de Vries D, Groot H, Trynka G, Hon C, Bonder M (2020). The single-cell eQTLGen consortium. eLife.

[53] Okada Y, Wu D, Trynka G, Raj T, Terao C, Ikari K (2014). Genetics of rheumatoid arthritis contributes to biology and drug discovery. Nature.

[54] Laufer VA, Tiwari HK, Reynolds RJ, Danila MI, Wang J, Edberg JC (2019). Genetic influences on susceptibility to rheumatoid arthritis in African-Americans. Hum Mol Genet.

[55] Ni J, Wang P, Yin KJ, Yang XK, Cen H, Sui C (2022). Novel insight into the aetiology of rheumatoid arthritis gained by a cross-tissue transcriptome-wide association study. RMD Open.

[56] Kasela S, Kisand K, Tserel L, Kaleviste E, Remm A, Fischer K (2017). Pathogenic implications for autoimmune mechanisms derived by comparative eQTL analysis of CD4+ versus CD8+ T cells. PLOS Genet.

[57] Stoeckius M, Hafemeister C, Stephenson W, Houck-Loomis B, Chattopadhyay PK, Swerdlow H (2017). Simultaneous epitope and transcriptome measurement in single cells. Nat Methods.

[58] Peterson VM, Zhang KX, Kumar N, Wong J, Li L, Wilson DC (2017). Multiplexed quantification of proteins and transcripts in single cells. Nat Biotechnol.

[59] Frei AP, Bava FA, Zunder ER, Hsieh EWY, Chen SY, Nolan GP (2016). Highly multiplexed simultaneous detection of RNAs and proteins in single cells. Nat Methods.

[60] Chen AF, Parks B, Kathiria AS, Ober-Reynolds B, Goronzy JJ, Greenleaf WJ (2022). NEAT-seq: simultaneous profiling of intra-nuclear proteins, chromatin accessibility and gene expression in single cells. Nat Methods.

[61] Chung H, Parkhurst CN, Magee EM, Phillips D, Habibi E, Chen F (2021). Joint single-cell measurements of nuclear proteins and RNA in vivo. Nat Methods.

[62] Ma A, McDermaid A, Xu J, Chang Y, Ma Q (2020). Integrative methods and practical challenges for single-cell multi-omics. Trends Biotechnol.

[63] Hao Y, Hao S, Andersen-Nissen E, Mauck WM, Zheng S, Butler A (2021). Integrated analysis of multimodal single-cell data. Cell.

[64] Westra HJ, Peters MJ, Esko T, Yaghootkar H, Schurmann C, Kettunen J (2013). Systematic identification of trans eQTLs as putative drivers of known disease associations. Nat Genet.

[65] Seabold S, Perktold J (2010). Statsmodels: econometric and statistical modeling with Python.

[66] Wu T, Hu E, Xu S, Chen M, Guo P, Dai Z, et al. clusterProfiler 4.0: a universal enrichment tool for interpreting omics data. The Innovation. 2021;2(3). [cited 2022 Mar 29]. Available from: https://www.cell.com/the-innovation/abstract/S2666-6758(21)00066-7 10.1016/j.xinn.2021.100141 PMC8454663 34557778

[67] Arnold M, Raffler J, Pfeufer A, Suhre K, Kastenmüller G (2015). SNiPA: an interactive, genetic variant-centered annotation browser. Bioinformatics.

[68] Lin SH, Brown DW, Machiela MJ (2020). LDtrait: an online tool for identifying published phenotype associations in linkage disequilibrium. Cancer Res.

[69] de Leeuw CA, Mooij JM, Heskes T, Posthuma D (2015). MAGMA: generalized gene-set analysis of GWAS data. Tang H, editor. PLOS Comput Biol..

[70] Chiou J, Geusz RJ, Okino ML, Han JY, Miller M, Melton R (2021). Interpreting type 1 diabetes risk with genetics and single-cell epigenomics. Nature.

[71] Wang G, Sarkar A, Carbonetto P, Stephens M (2020). A simple new approach to variable selection in regression, with application to genetic fine mapping. J R Stat Soc Ser B Stat Methodol.

[72] Li S, Schmid KT, de Vries DH, Korshevniuk M, Losert C, Oelen R, et al. Single cell co-expression QTL analysis. GitHub; 2023 [cited 2023 Feb 9]. Available from: https://github.com/sc-eQTLgen-consortium/li-2023

[73] Li S, Schmid KT, de Vries DH, Korshevniuk M, Losert C, Oelen R, et al. sc-eQTLgen-consortium/li-2023: First release. Zenodo; 2023 [cited 2023 Feb 9]. Available from: https://zenodo.org/record/7623968

[74] van der Wijst MGP, Brugge H, de Vries DH, Deelen P, Swertz MA, LifeLines Cohort Study, et al. Single-cell RNA sequencing reveals cell-type specific eQTLs in peripheral blood mononuclear cells - EGA European Genome-Phenome Archive. [cited 2023 Jan 28]. Available from: https://ega-archive.org/studies/EGAS00001002560

[75] Oelen R, de Vries DH, Brugge H, Gordon MG, Vochteloo M, single-cell eQTLGen consortium, et al. Single-cell RNA sequencing dissects gene-environment interactions on gene expression and regulation in immune cells. - EGA European Genome-Phenome Archive. [cited 2023 Jan 28]. Available from: https://ega-archive.org/studies/EGAS00001005376

[76] van Blokland I, Oelen R, de Groot H, van der Harst P, Franke L, van der Wijst MGP. 38 STEMI patients at hospital admission, 24 hours (acute phase) and 6-8 weeks (chronic phase) after STEMI - EGA European Genome-Phenome Archive. [cited 2023 Feb 25]. Available from: https://ega-archive.org/datasets/EGAD00001010064

[77] BIOS Consortium. The BIOS Consortium: Biobank-based Integrative Omics Studies - EGA European Genome-Phenome Archive. [cited 2023 Feb 27]. Available from: https://ega-archive.org/dacs/EGAC00001000277

