# Supplementary material for: Identification of genetic variants that impact gene co-expression relationships using large-scale single-cell data
Source: Genome Biol. 2023 Apr 18;24:80. doi: 10.1186/s13059-023-02897-x (PMC10111756; doi:10.1186/s13059-023-02897-x)
Supplement: Supplementary file 2 — Additional file 2: Supplementary Figures. Collection of all Fig. S1-34. [file 13059_2023_2897_MOESM2_ESM.pdf]

Supplementary Figures

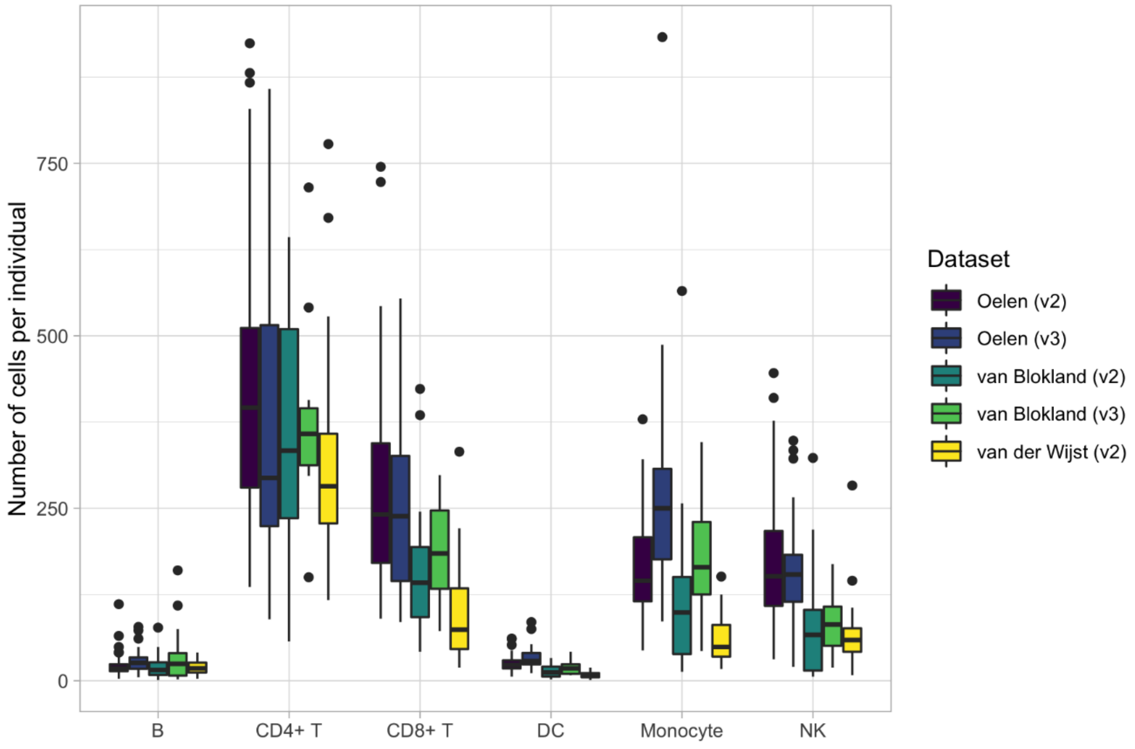

Fig. S1. *Number of cells per cell type and individual for each dataset*

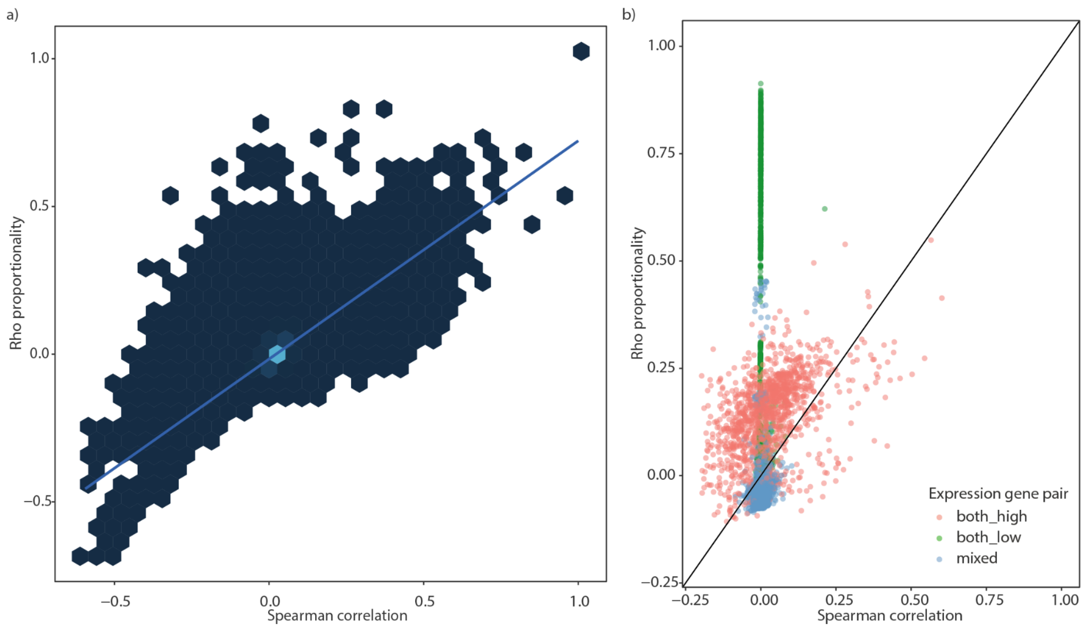

Fig. S2. *Comparison between proportionality (Rho) and Spearman correlation.* ***a)*** *Comparison for genes that are expressed in at least 5% of the monocytes in the Oelen v3 dataset. The color indicates the density (light color for higher density).* ***b)*** *Comparison for very highly expressed genes (expressed in at least 90% of the cells) and very lowly expressed genes (expressed in 0-5%), both times sampling 50 examples to increase visibility of the scatter plot. Colors represent type of gene pair (either both highly expressed, both lowly expressed or one highly and one lowly expressed).*

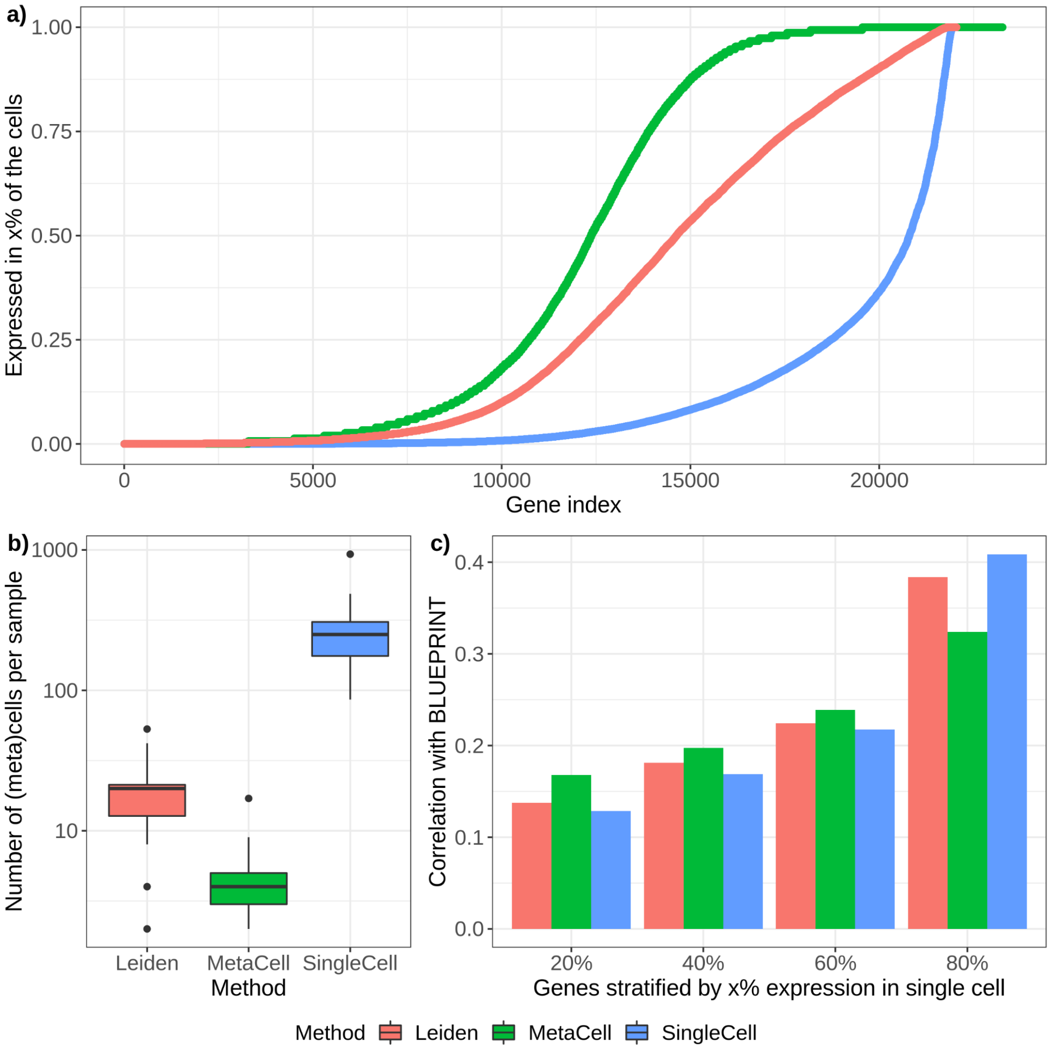

Fig. S3. *Grouping cells to meta-cells*

Similar cells were grouped to meta-cells, using either the original MetaCell algorithm (parameters shown in the plot: K=20, minCells=10) or our own implementation based on the Leiden algorithm (parameters shown in the plot: resolution=20) (see Methods for detail). All methods were applied to the Oelen v3 dataset, Monocytes. ***a)*** *Both meta-cells generated from Leiden clustering and from the MetaCell algorithm lead to more genes expressed in at least x% of the cells compared to the original single cell data (visualized here via a cumulative density function).* ***b)*** *In contrast, the number of (meta)cells per sample is reduced with both algorithms drastically, this way reducing the number of measurement points to infer the correlation per sample.* ***c)*** *To*

benchmark the performance, the correlation with the BLUEPRINT bulk dataset was calculated (compared with Main Fig. 2b). To evaluate how lower expressed genes are affected by the meta-cell grouping, the correlation is calculated separately for gene pairs were the non-zero expression level of both genes is between 20%-40%, 40%-60%, 60%-80% and 80%-100% of the cells (showing the first number on the x-axis).

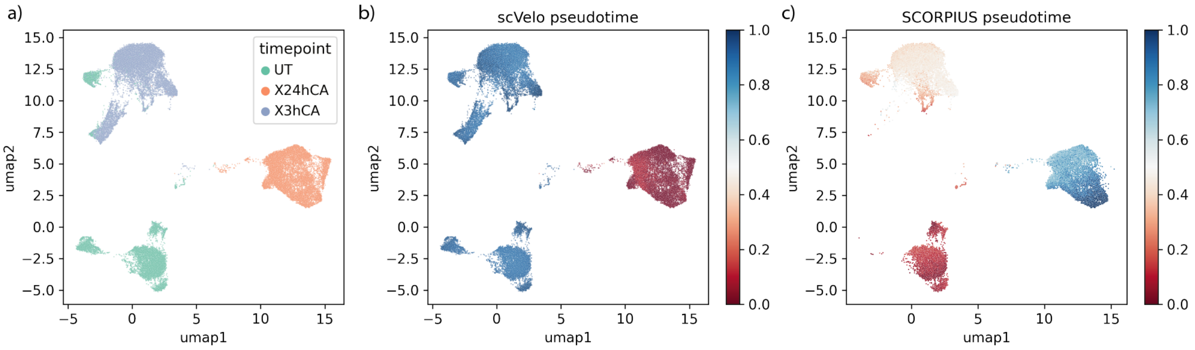

Fig.S4. *Comparison for the inferred time-stamp information from RNA velocity with scVelo and pseudo-time ordering algorithm SCORPIUS, and the sample time for the single cell data collection during stimulation experiments. Panel a) shows the experiment time points: UT stands for untreated status, X24hCA stands for treated with Candida for 24 hours, X3hCA stands for treated with Candida for 3 hours. Panel b) shows the pseudotime predicted by scVelo. Panel c) shows the pseudotime predicted by SCOPIUS.*

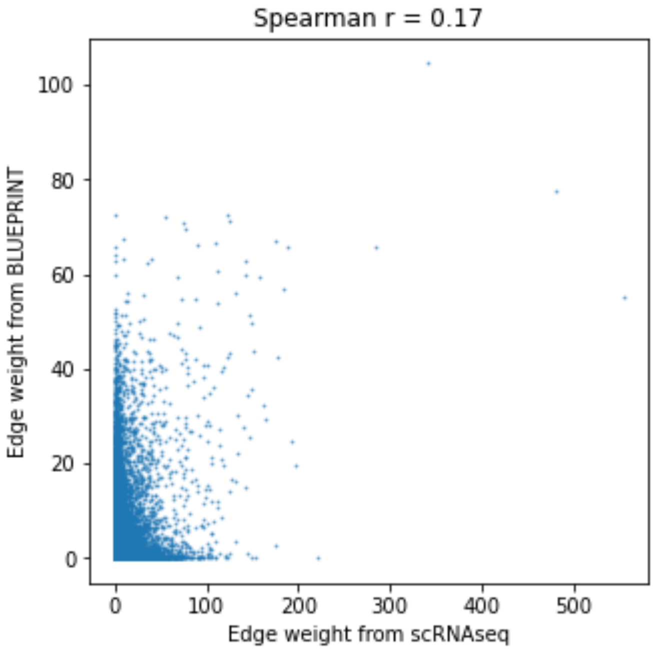

Fig. S5. *Prediction performance comparison for gene pairs (genes that expressed in more than 50% of the monocytes) between single cell monocytes and BLUEPRINT monocytes, for both data, the predicted gene pairs are from GRNBoost2 method*

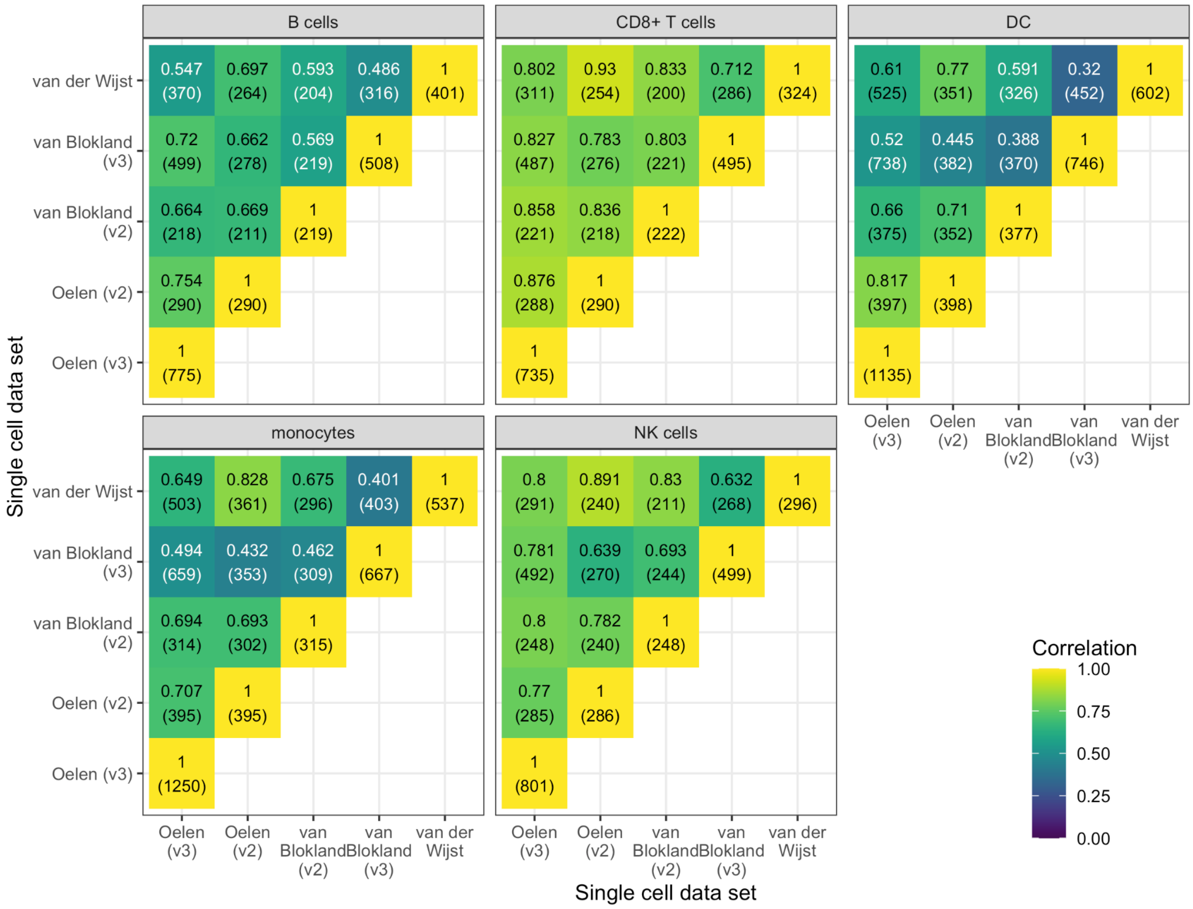

Fig. S6. *Extension of Main Fig. 2a for other cell types, comparing different single-cell datasets. Spearman correlation of the the Oelen v3 and v2 datasets, the van Blokland v2 and v3 datasets and the van der Wijst dataset were compared with each other, taking genes expressed in at least 50% of the cells in the corresponding datasets.*

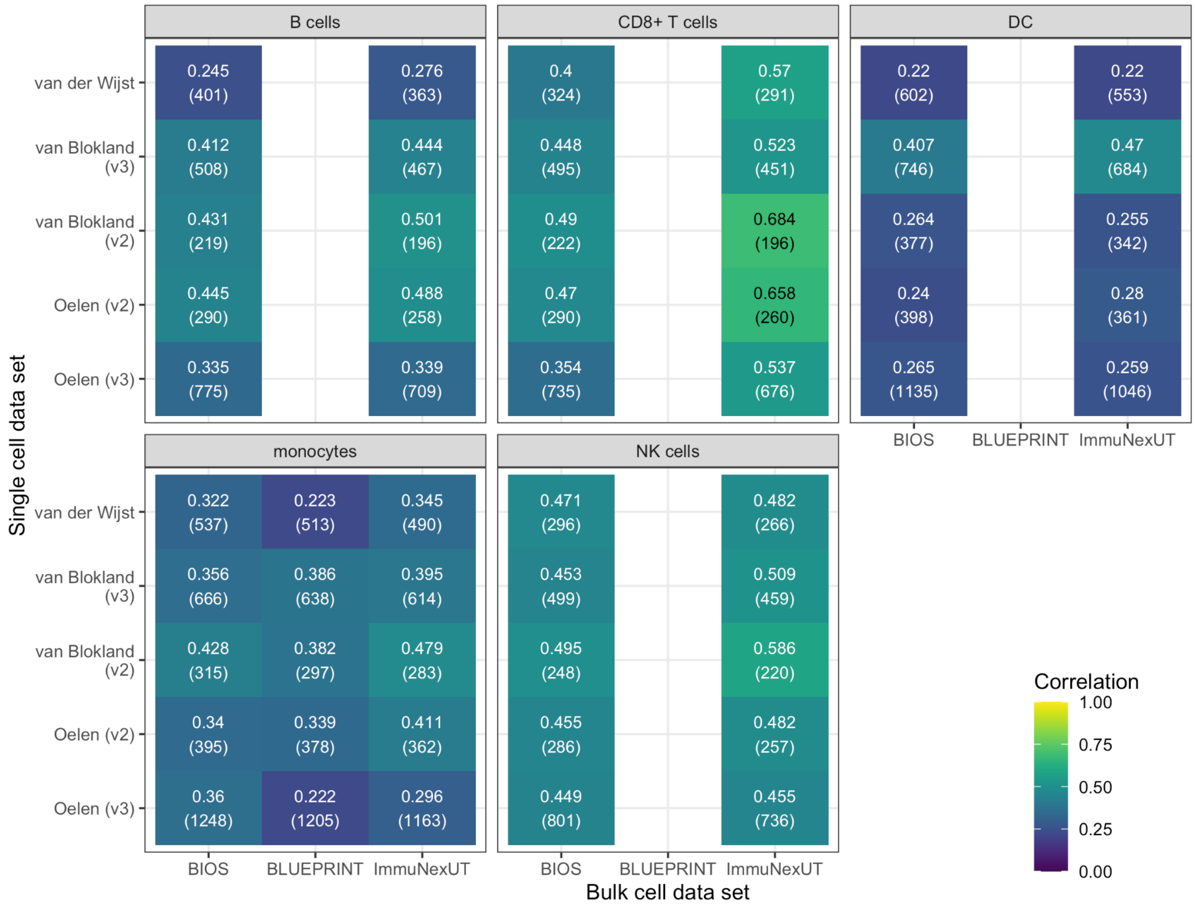

Fig. S7. *Extension of Main Fig. 2b for other cell types: Comparison of the co-expression profiles between the single-cell datasets with the bulk RNA-seq datasets from BLUEPRINT, ImmuNexUT (both measuring FACS sorted cell types) and BIOS (whole blood). For BLUEPRINT, classical monocytes were measured, for ImmuNexUT, the compared cell types were naive B cells, naive CD8+ T cells, myeloid DCs, classical monocytes and NK cells. Again only genes were taken that were expressed in at least 50% of the cells for the single-cell dataset. The number of tested genes is shown in brackets in each square below the exact Spearman correlation value.*

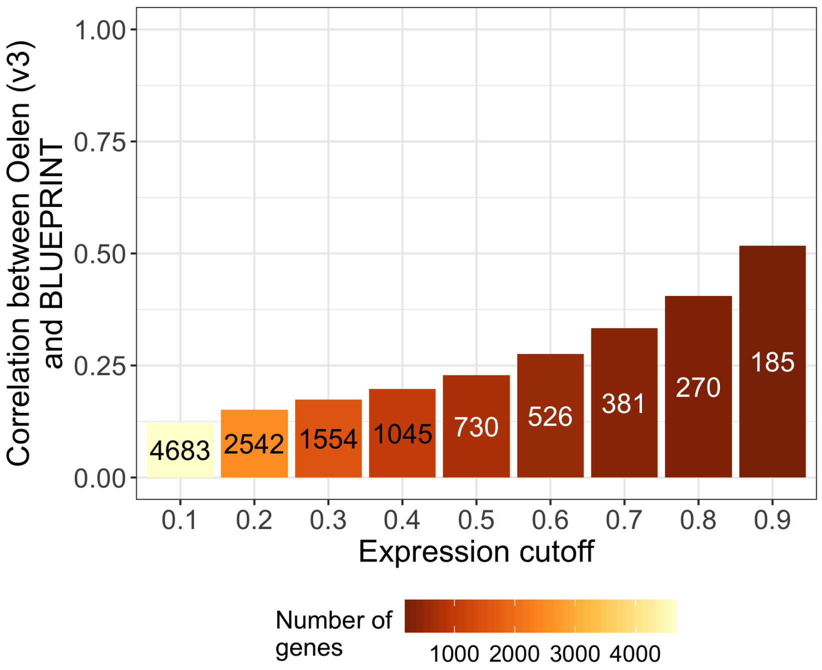

Fig. S8. *Analysis from Main Fig. 2c with the BLUEPRINT dataset instead of the ImmuNexUT dataset: Relationship between the co-expression similarity between the BLUEPRINT naive CD4+ T cells and Oelen v3 dataset CD4+ T cells and increasing gene expression cutoffs (the ratio of cells with non-zero expression for a given gene). Both the color scale and the numbers in the bar plot show the number of tested genes.*

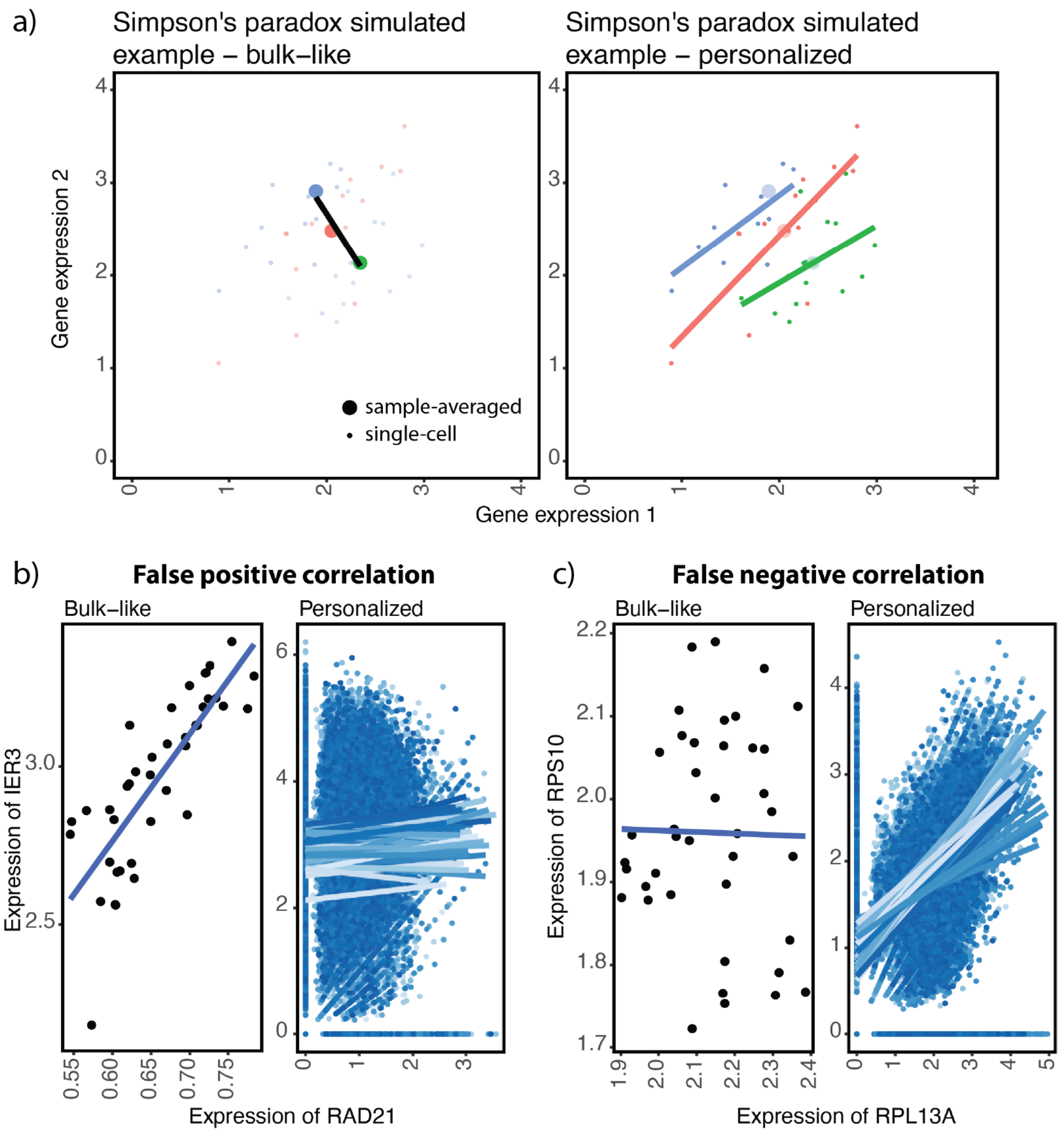

Fig. S9. Simpson's paradox in expression data a) *A simulated example showing how Simpson's paradox can appear in expression data. The colors of the dots represent the sample. Each small dot represents a cell and the large dots represent the average expression across cells for that individual. The line in the left figure is the regression line for the sample-averaged dots and the lines on the right are the regression lines for all cells for each of the three samples.* ***b)*** *An example showing a false positive correlation identified by the bulk-like expression data but not identified in the personalized manner* ***c)*** *An example showing a false negative correlation not*

identified if aggregating the scRNA-seq data with the bulk-like approach, but a true correlation identified by the personalized expression data.

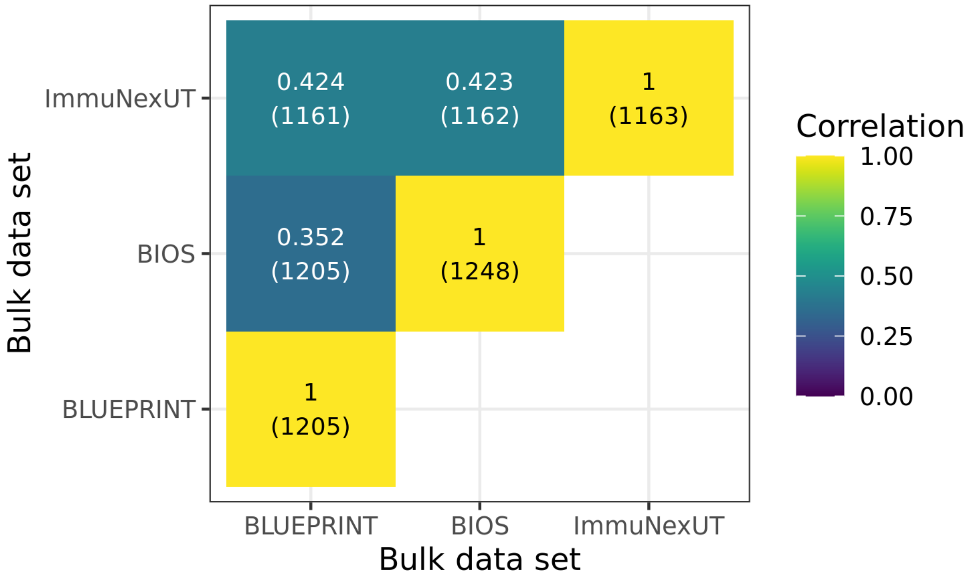

***Fig. S10.** Extension of Main Fig. 2d for monocytes: Comparison of the co-expression profiles between the bulk RNA-seq datasets from BLUEPRINT, ImmuNexUT (both measuring FACS sorted classical monocytes) and BIOS (whole blood). In all datasets, only genes expressed in 50% of the cells from the Oelen v3 dataset were selected, to make it comparable with Fig. S6 and S7. The number of tested genes is shown in brackets in each square below the exact Spearman correlation value.*

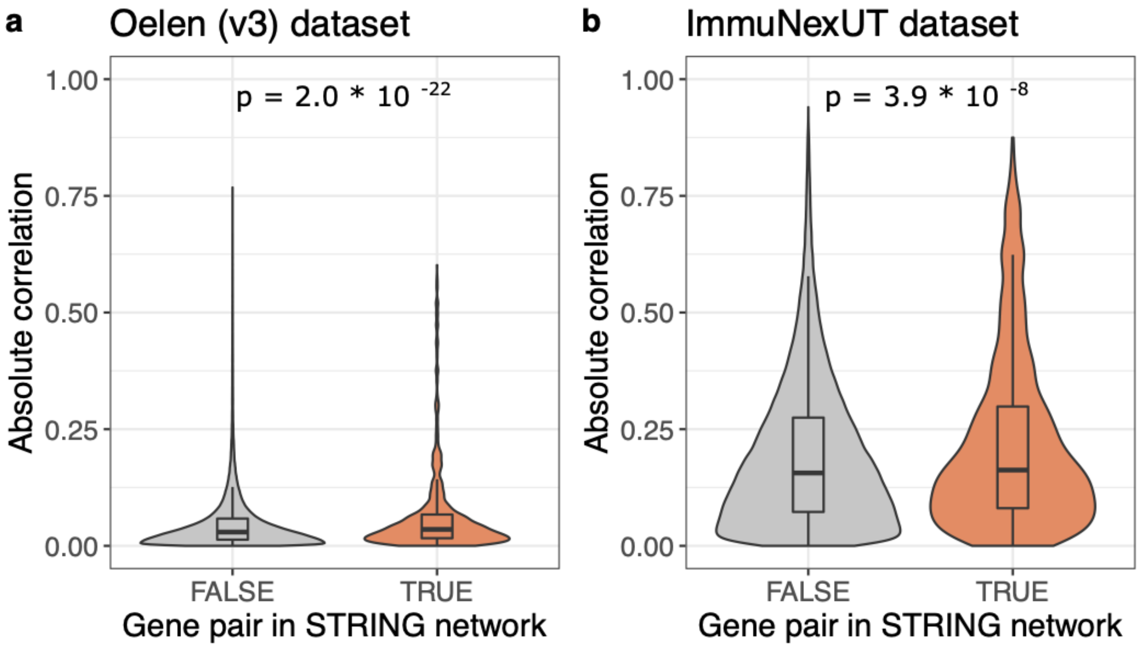

Fig. S11. *Enrichment of correlated genes among gene pairs whose proteins are interacting according to the STRING database, taking correlation values from Oelen v3 single-cell dataset in* ***a)*** *and ImmuNexUT bulk dataset in* ***b)****. P-values in the plot show the significance level of the Wilcoxon rank sum test.*

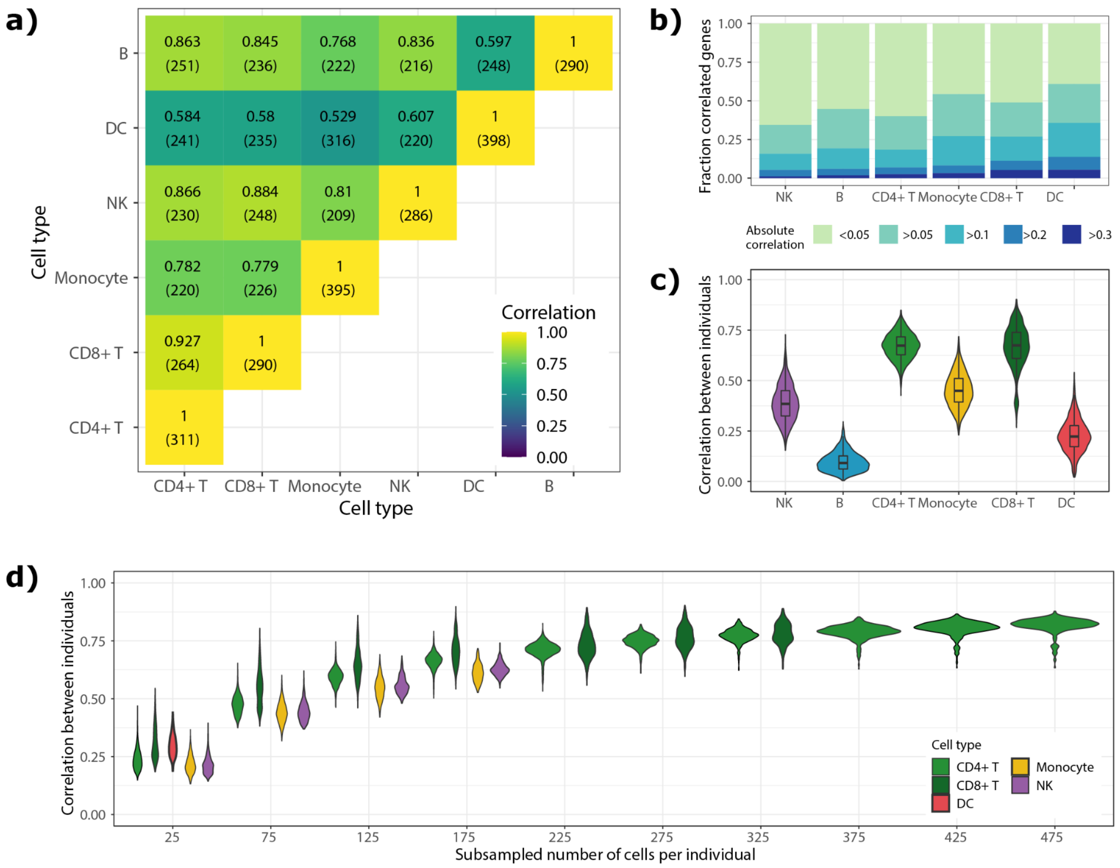

Fig. S12. *Adaptation of Main Fig. 3 for Oelen v2 dataset instead of Oelen v3 dataset, showing the same trends: Each analysis was done for all gene expressed in at least 50% of the cells for the respective cell type.* ***a)*** *Comparing co-expression patterns across cell types within the Oelen v2 dataset, for genes expressed in 50% of the cells for both cell types in each pairwise comparison (same approach as in Main Fig. 2a-c)* ***b)*** *Correlation distribution within each cell type* ***c)*** *Correlation between different individuals within each cell type, showing the distribution of all pairwise comparisons between individuals.* ***d)*** *Dependence of number of cells on the correlation between individuals, separately for each cell type. In each subsampling step, all individuals are taken that have at least this number of cells and subsampled to exactly the number (this leads to removal of some individuals for higher number of cells). B cells were not frequent enough to evaluate it in this dataset.*

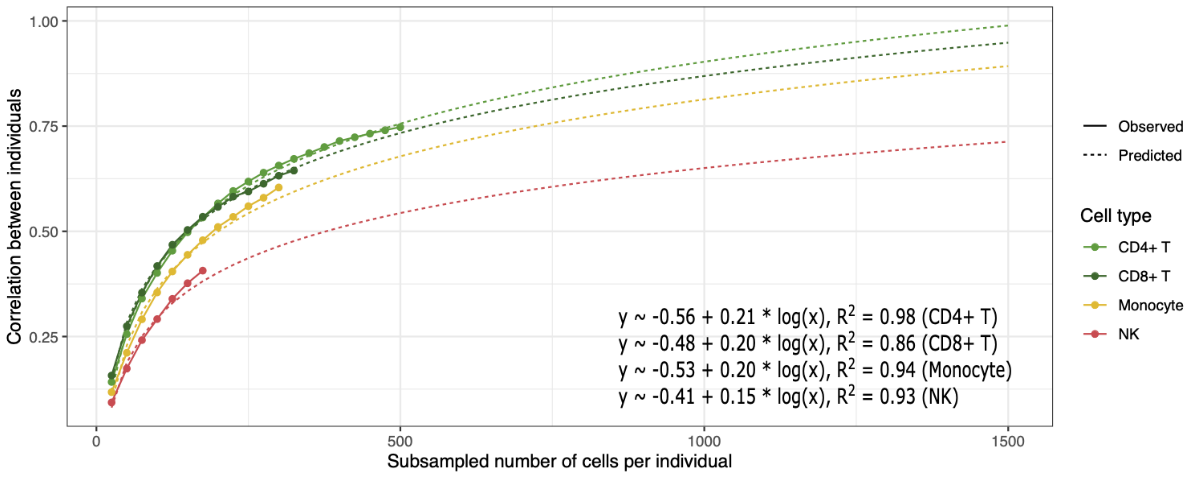

Fig. S13. *Fitting a logarithmic curve (based on the natural logarithm) for the four most frequent cell types (CD4+ T cells, CD8+ T cells, monocytes, NK cells) to explain the correlation value between individuals by the number of cells per individuals (estimated curves and adjusted R^2 values for each cell type in the text). Dotted line shows extrapolation of this fit to predict correlation when increasing the number of cells up to 1,500 cells per individual and cell type.*

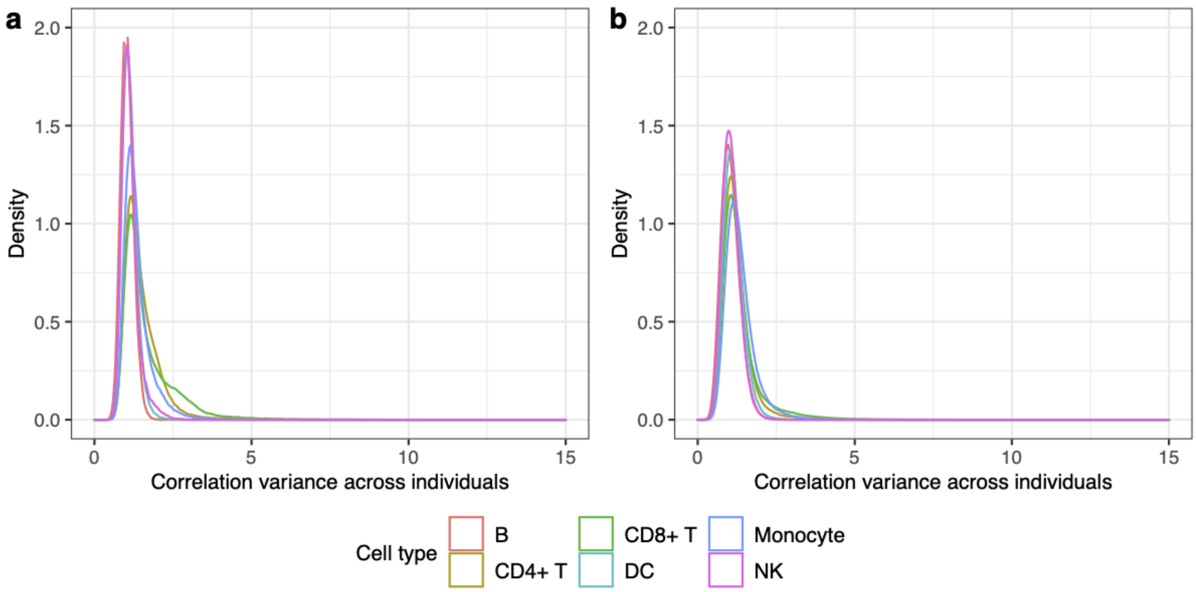

Fig. S14. *Variance of gene pairs (correlation z-scores) across individuals per cell type for Oelen v2 dataset in* ***a)*** *and Oelen v3 dataset in* ***b)****.*

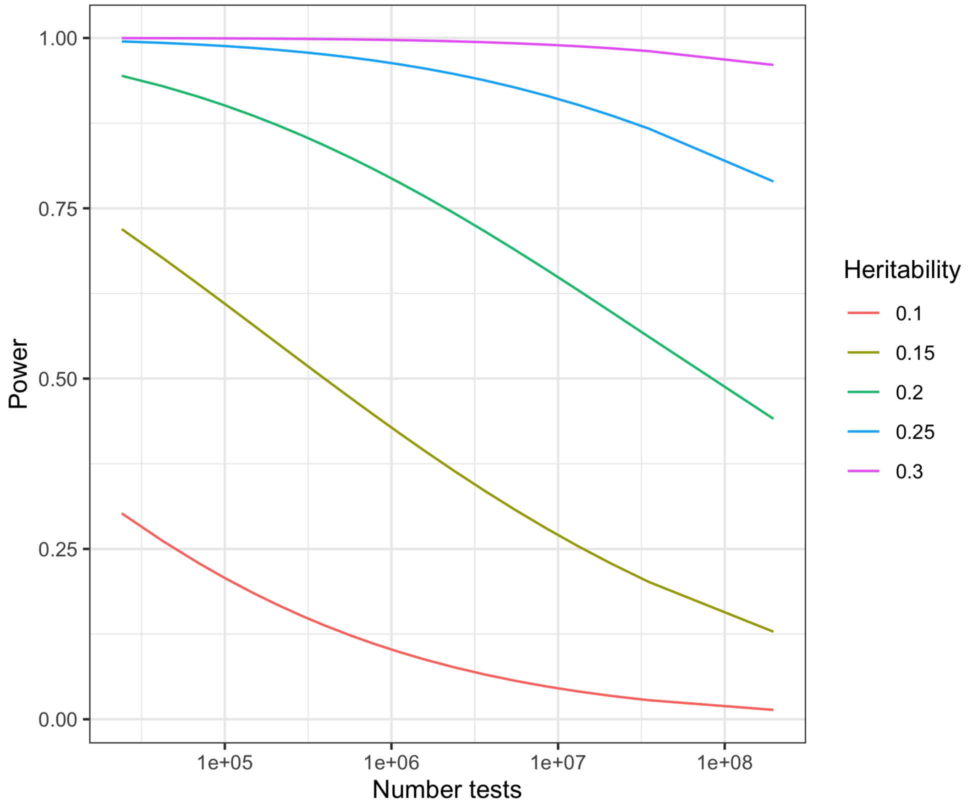

Fig. S15. *Power analysis coe-QTLs. The power to detect a co-eQTL with a certain heritability is calculated based on a Bonferroni-corrected significant threshold of 0.05 and a sample size of 173. The multiple testing correction is strongly affected by the number of tests on the x-axis. The maximum number of tests in the plot represents testing all genes against each other that are expressed in monocytes for the Oelen v3 dataset, but testing only one SNP per pair.*

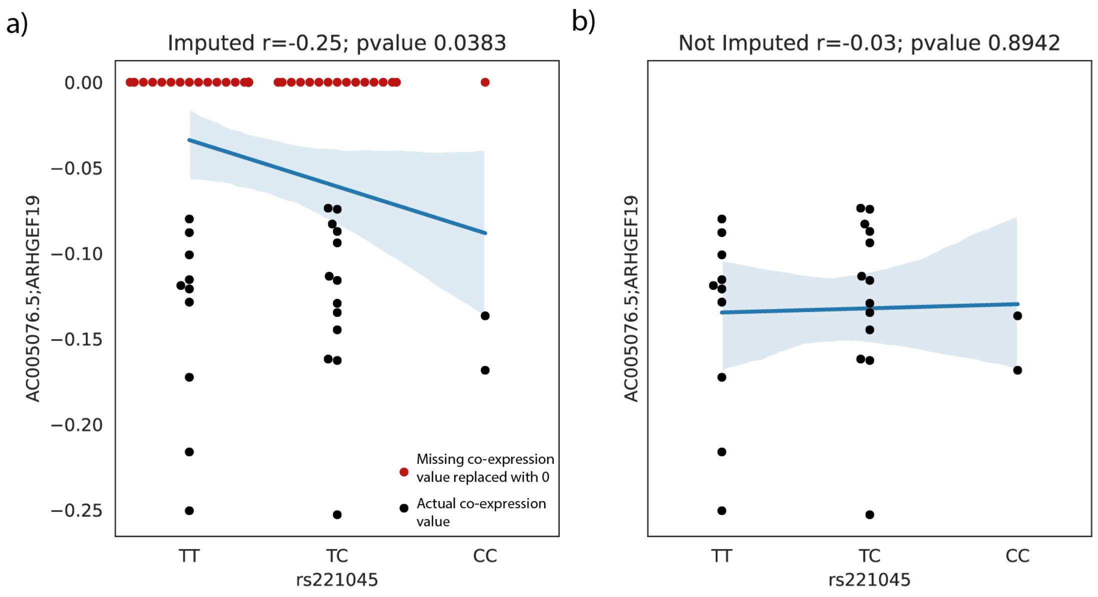

Fig. S16. *Effect of replacing NaN values for the coeQTL analysis.* ***a)*** *The false positive co-eQTL identified if we replaced the NaN values to zeros.* ***b)*** *The absence of co-eQTL effects if we did not replace the NaN values to zeros.*

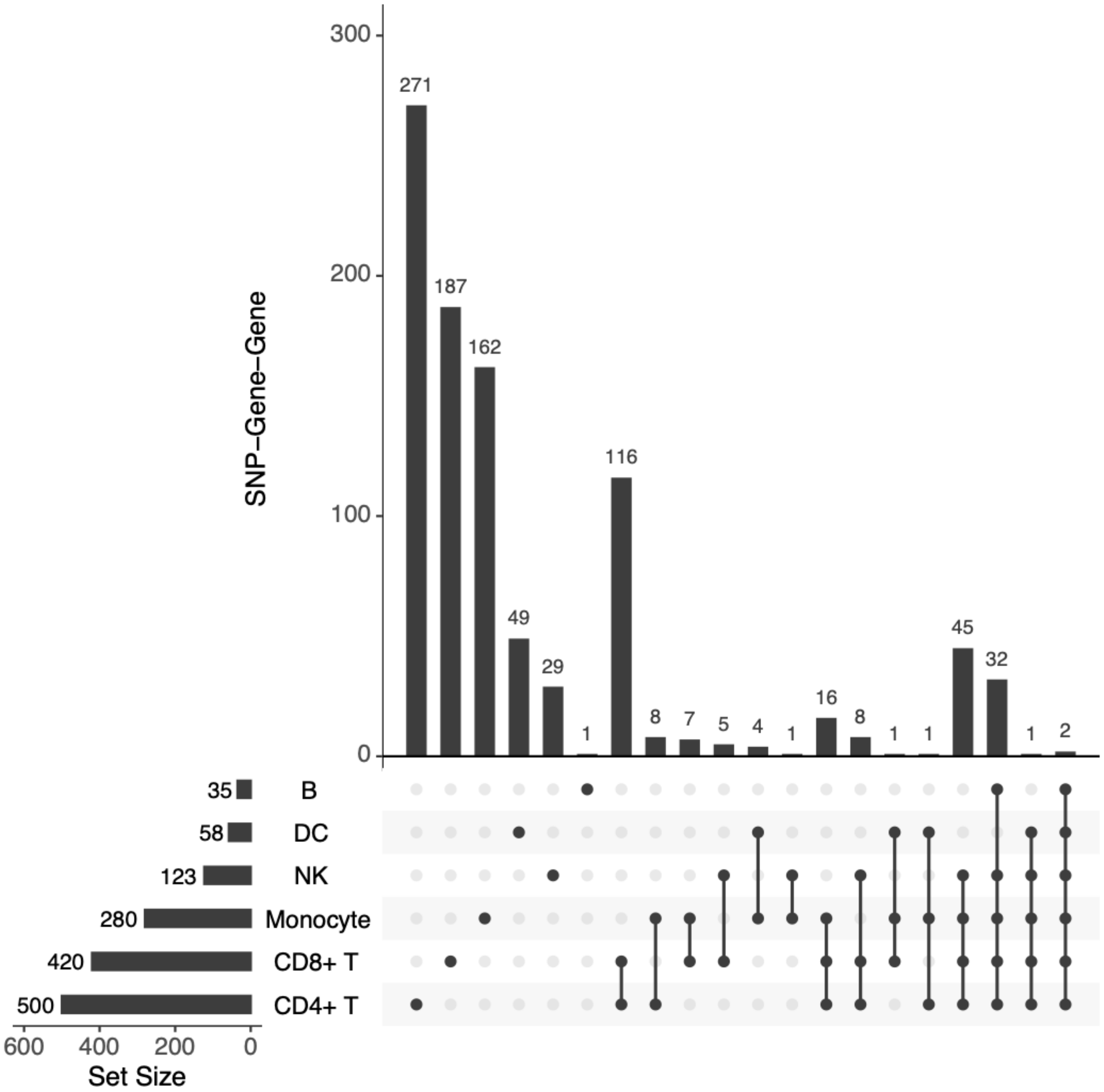

Fig. S17. *Upset plot with overlap of significant co-eQTLs between cell types.*

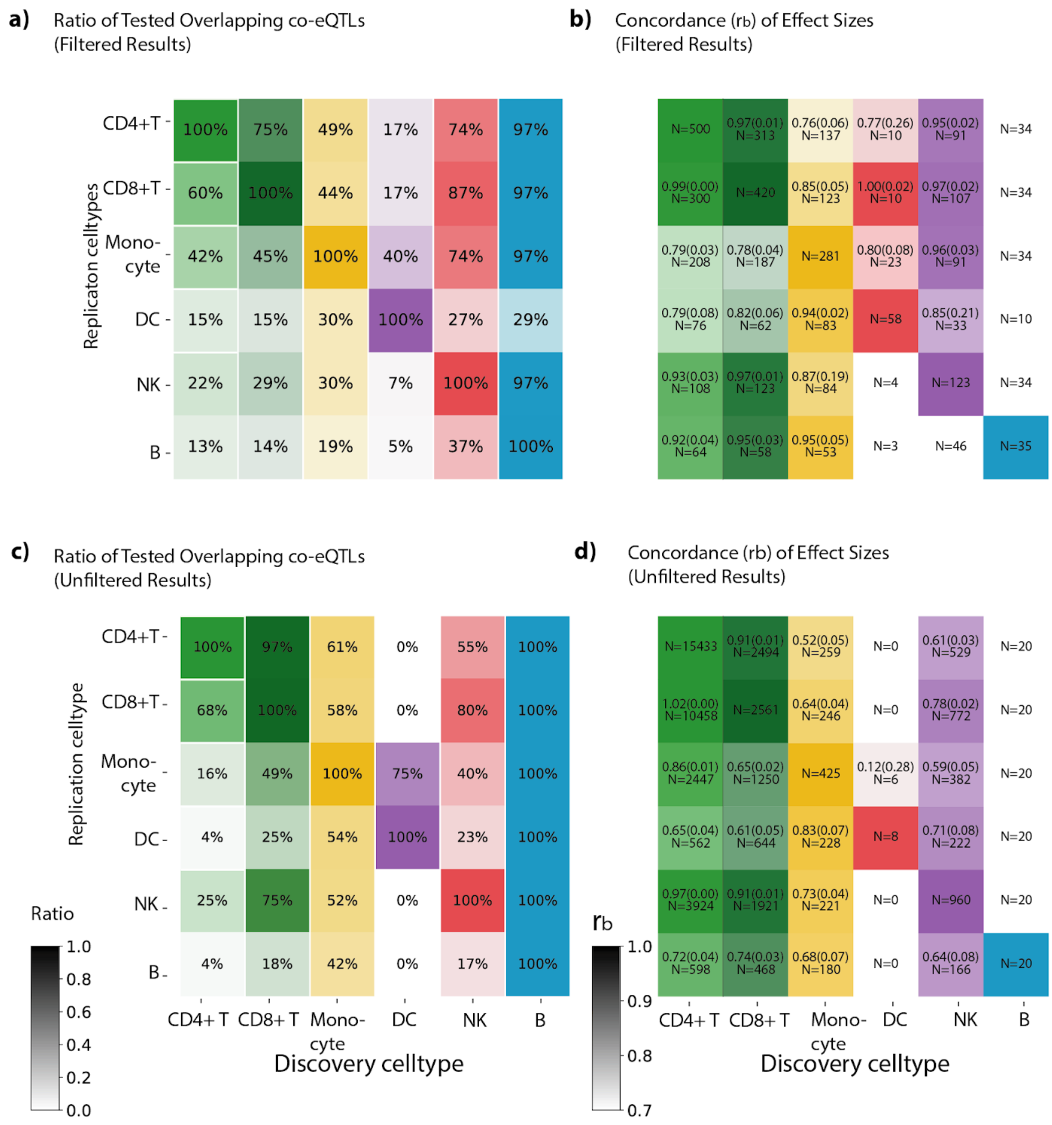

***Fig. S18**. The cell type specificity of co-eQTLs identified in each cell type. Here, we replicated the identified co-eQTLs from each cell type in other cell types and used two measures to show the replication performance: the ratio of tested co-eQTLs in replication, and the concordance of effect sizes shown with the r_b values. Panel **a)** shows the ratio of tested co-eQTLs in replications for the identified co-eQTLs identified with the filtering strategy. Panel **b)** shows the r_b values for co-eQTLs identified with the filtering strategy (standard error in parentheses, number of co-eQTLs below). Panel **c)** shows the ratio of tested co-eQTLs in replications for the identified co-eQTLs identified without the filtering strategy. Panel **d)** shows the r_b values for co-eQTLs identified*

without the filtering strategy (standard error in parentheses, number of co-eQTLs below).

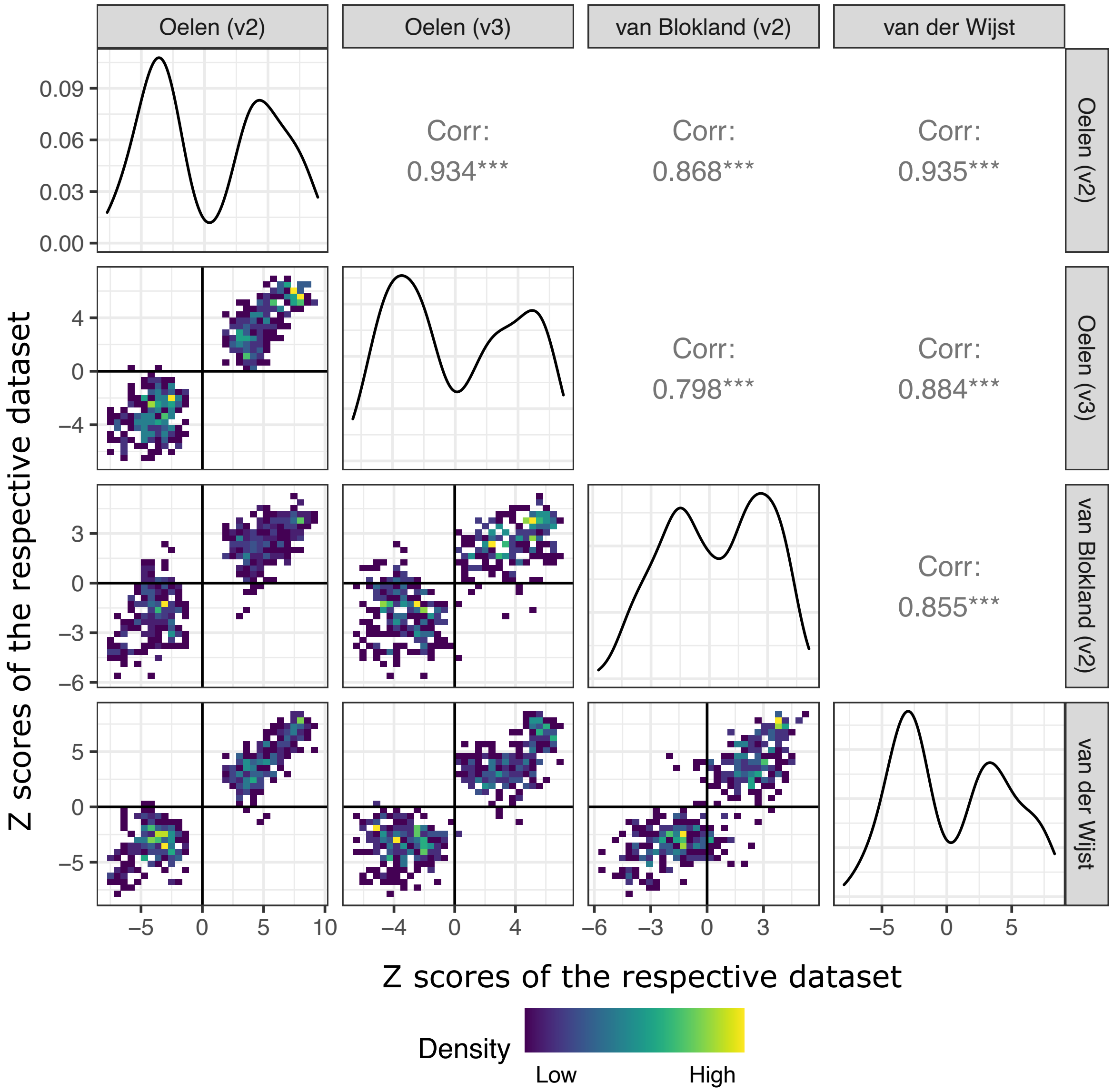

Fig. S19. *Comparison of Z-scores across datasets. Distribution of significant co-eQTL Z-scores per dataset, that was included in the meta-analysis, for the CD4+ T cells. The plot shows scatter density plots between cohorts (lower triangle), distributions within the cohort (diagonal) and correlations between cohorts (upper triangle).*

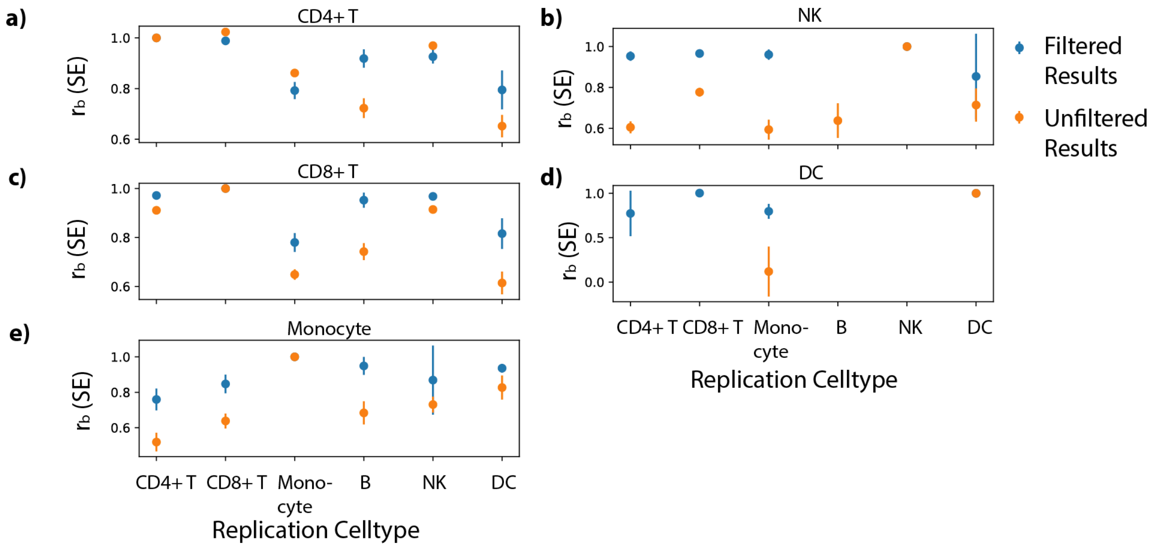

***Fig. S20**. Comparison of the rb values from the replication cell types between the co-eQTLs identified with the filtering strategy and the co-eQTLs identified without the filtering strategy.*

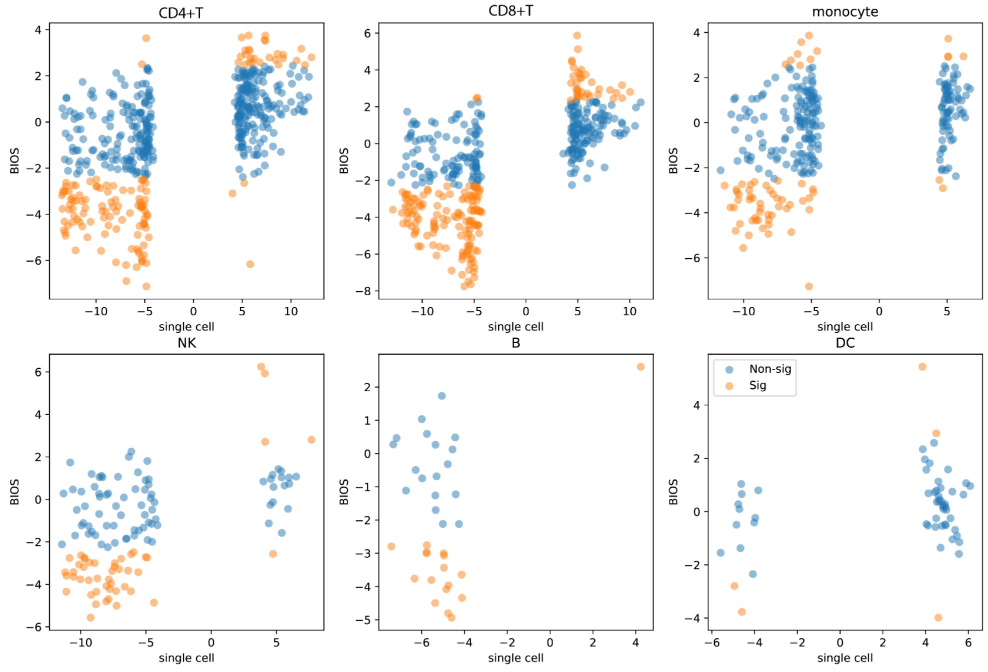

***Fig. S21.** BIOS replication for co-eQTLs identified with the filtering strategy for each cell type. The blue points labeled with "Non-sig" represent the co-eQTLs that could not*

be significantly replicated in BIOS. The orange points labeled with “Sig” represent the co-eQTLs that were replicated significantly in BIOS.

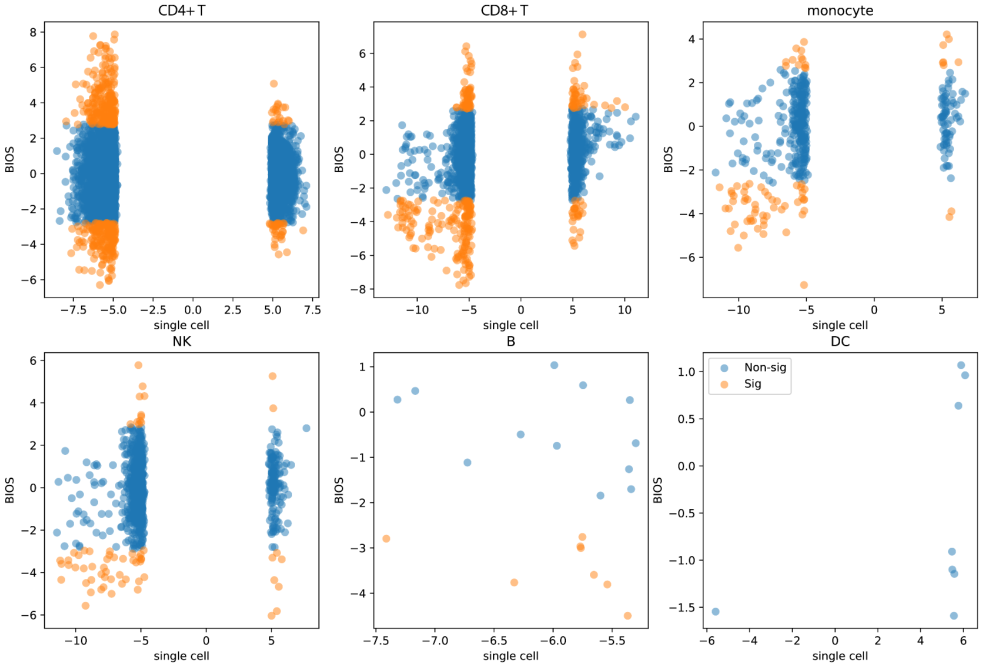

Fig. S22. *BIOS replication for co-eQTLs identified without the filtering strategy for each cell type. The blue points labeled with “Non-sig” represent the co-eQTLs that could not be significantly replicated in BIOS. The orange points labeled with “Sig” represent the co-eQTLs that were replicated significantly in BIOS.*

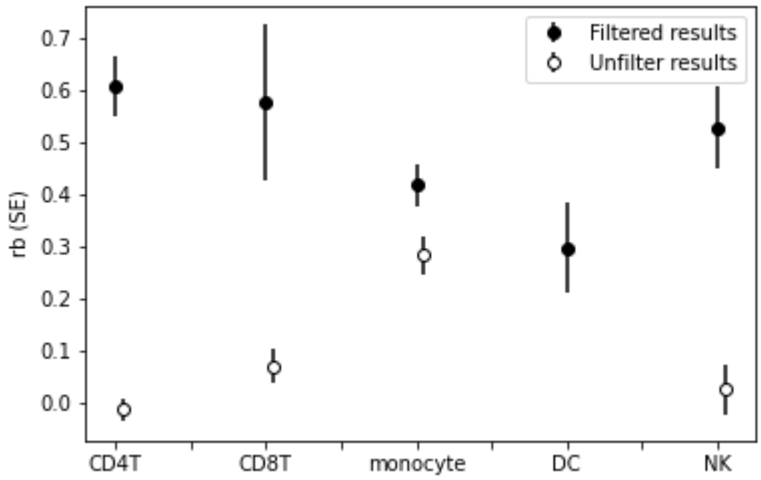

Fig. S23. *Comparison of rb values from BIOS replication analysis between co-eQTLs identified with the filtering strategy and that without the filtering strategy.*

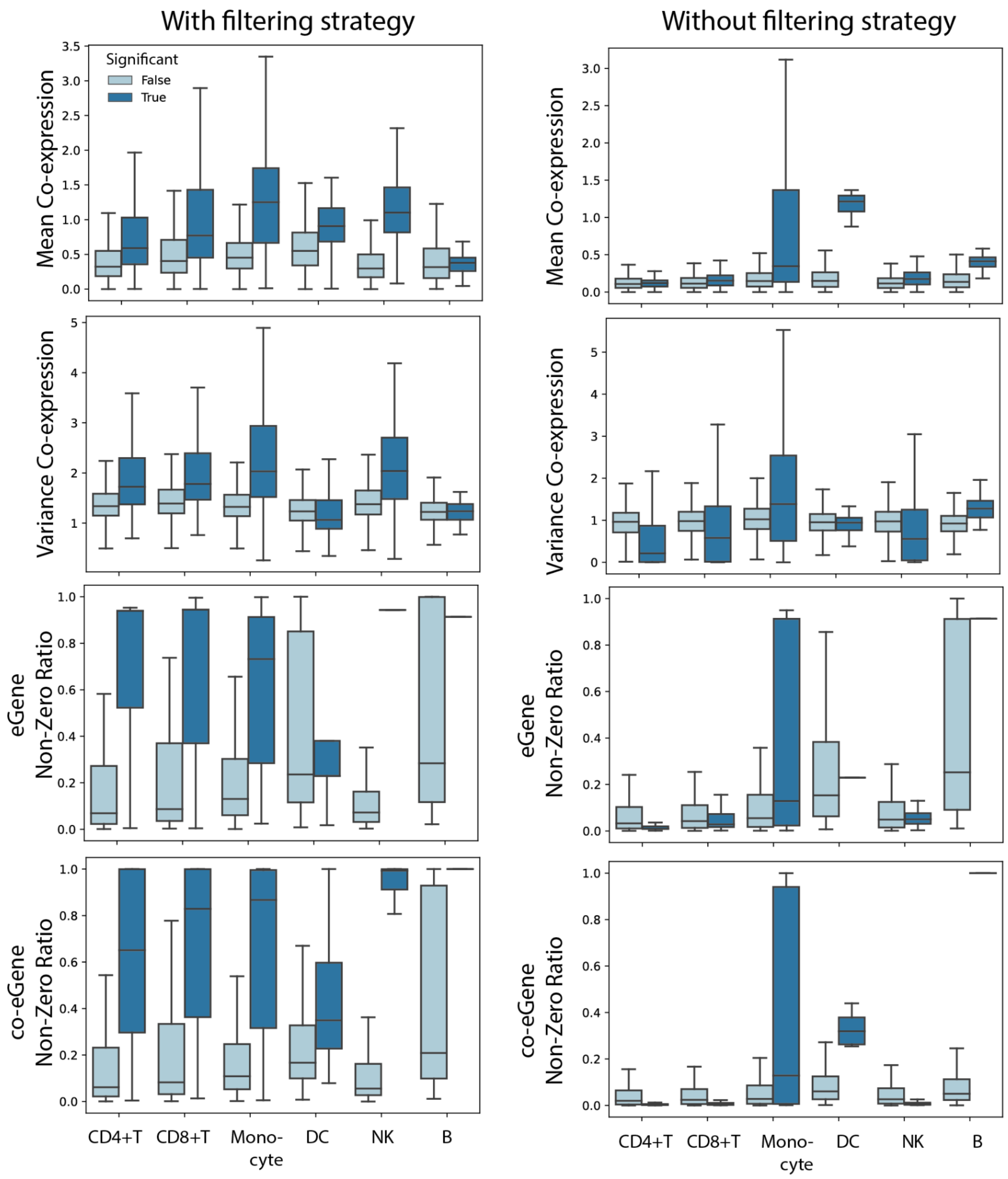

Fig. S24. *Comparison between the co-eQTLs obtained with and without the filtering strategy. Panel* ***a, c, e, g*** *show values from the co-eQTLs obtained with the filtering strategy. Panel* ***b, d, f, g*** *show values from the co-eQTLs obtained without the filtering strategy. Panel a and b shows the comparison of co-expression mean values among individuals from the Oelen v2 data for different cell types between the significant co-eQTLs and the insignificant SNP-eGene-co-eGene triplets. Panel c and d shows the comparison of co-expression variances among individuals from the Oelen v2 data for*

different cell types between the significant co-eQTLs and the insignificant SNP-eGene-co-eGene triplets. Panel e and g shows the comparison of eQTL gene non-zero ratio (the percentage of cells where this gene is expressed) from the Oelen v2 data for different cell types between the significant co-eQTLs and the insignificant SNP-eGene-co-eGene triplets.

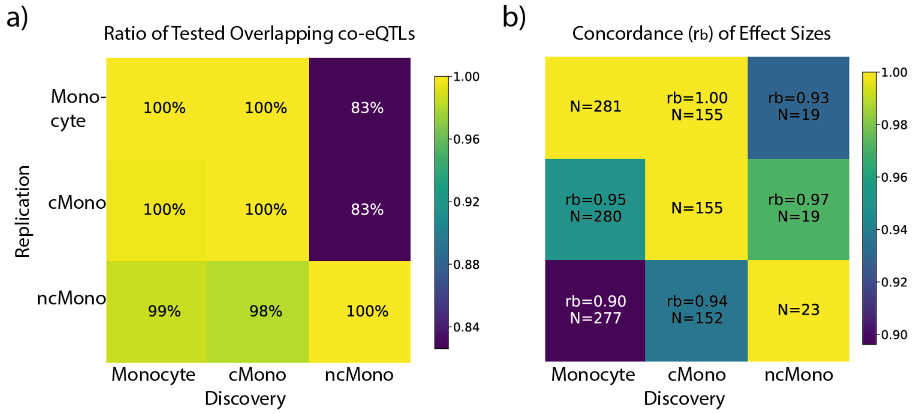

Fig. S25. *Impact of subcell types. Here we showed the replication performance of co-eQTLs identified with the filtering strategy in Monocytes, classical monocytes (cMono) and non-classical monocytes (ncMono) in each other. Panel* ***a)*** *shows the ratio of tested co-eQTLs in the replications. Panel* ***b)*** *shows the rb values for each replication.*

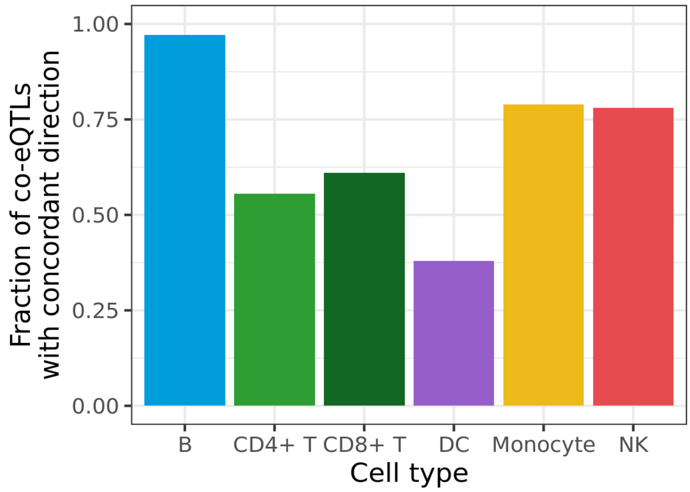

Fig. S26. *Fractions of co-eQTLs in each cell type that have a concordant direction of effect compared to the associated eQTL (i.e. the correlation increases when the expression of the eGene increases).*

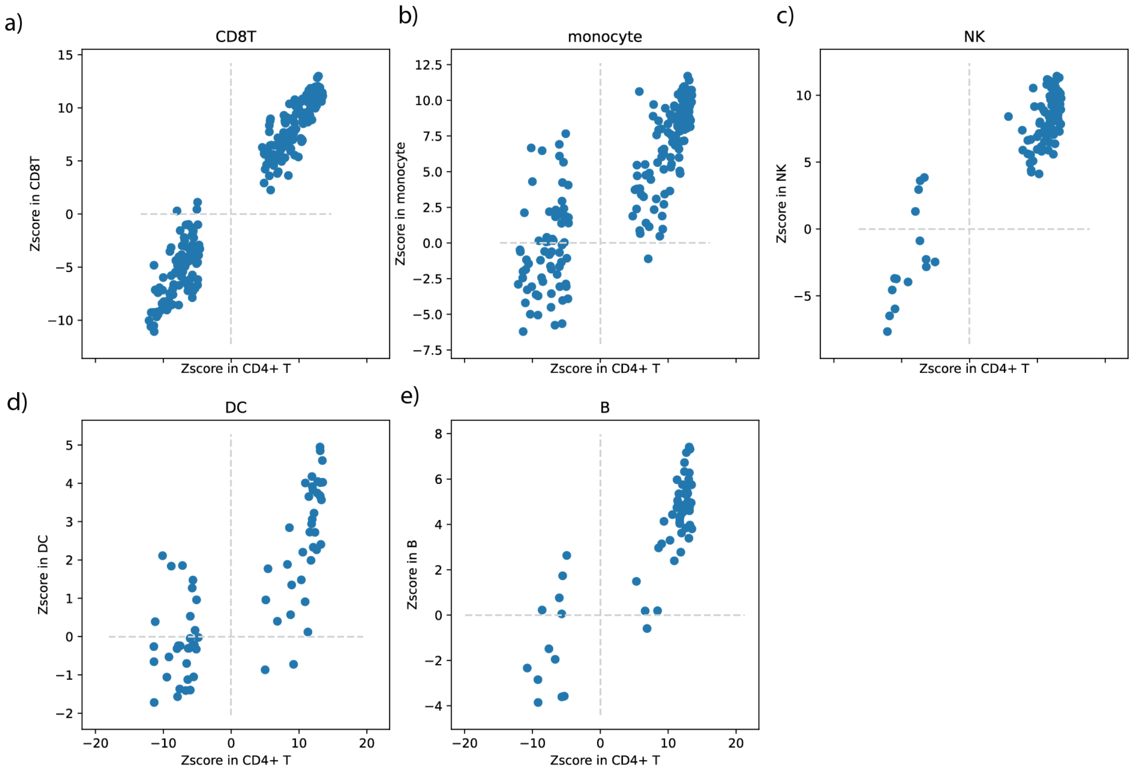

Fig. S27. *The replication results for co-eQTLs with RPS26 being the eGene identified in CD4+ T cells in other cell types. Each panel shows the replication performance in the corresponding cell type as indicated in the panel titles.*

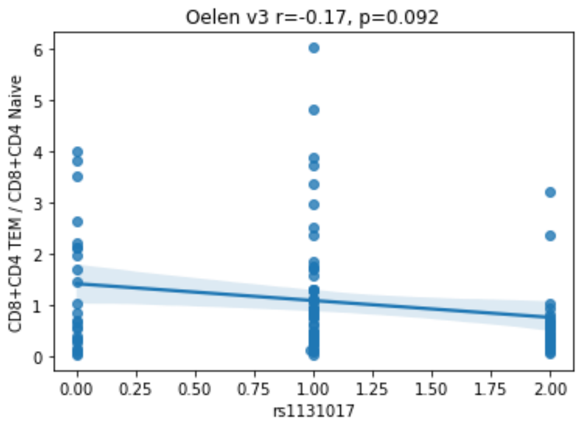

Fig. S28. *The association between SNP rs11311017 and the ratio between CD4+ & CD8+ TEM / CD4+ & CD8+ Naive T cells*

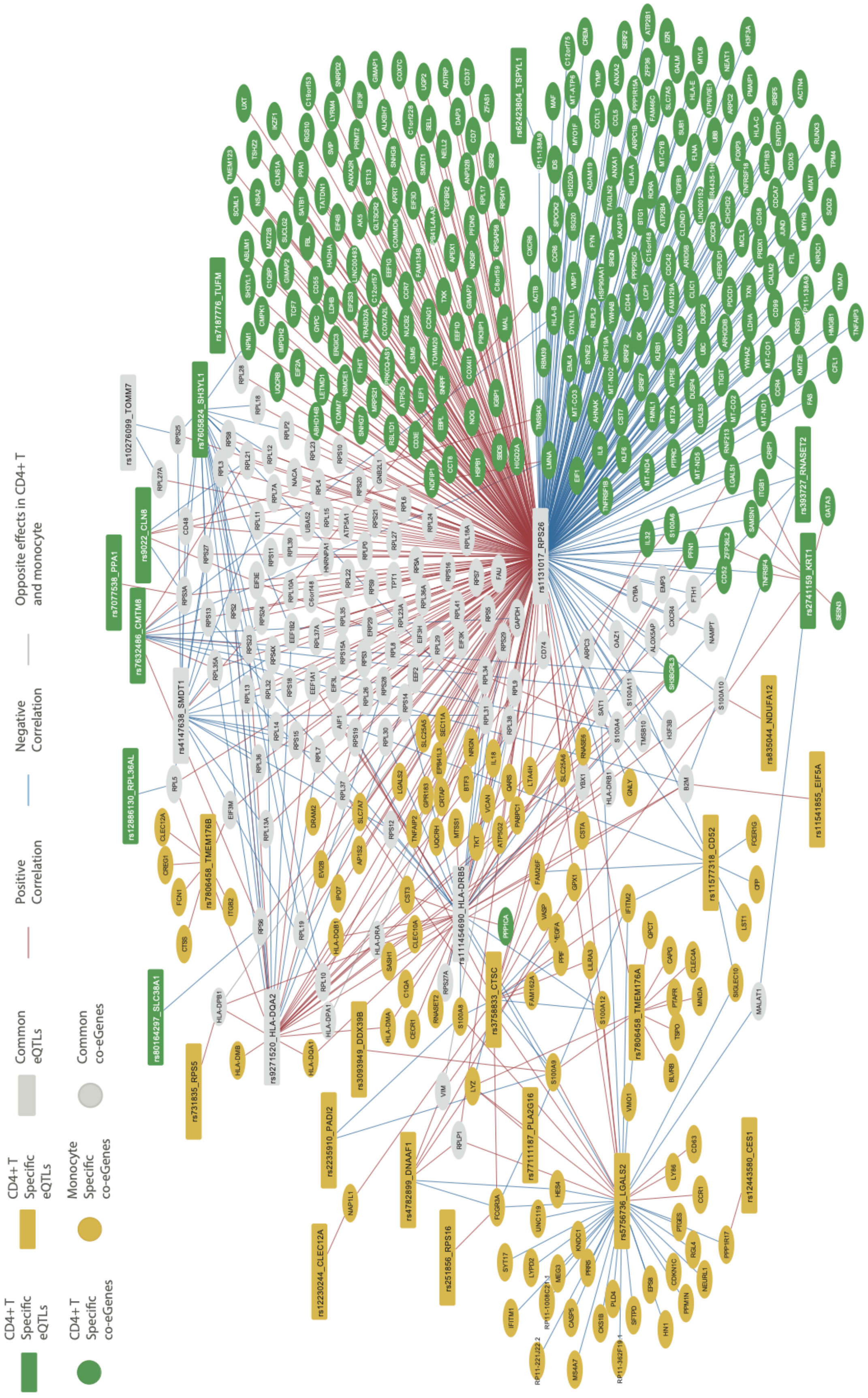

Fig. S29. *Complete CD4+ T and Monocyte co-eQTL network*

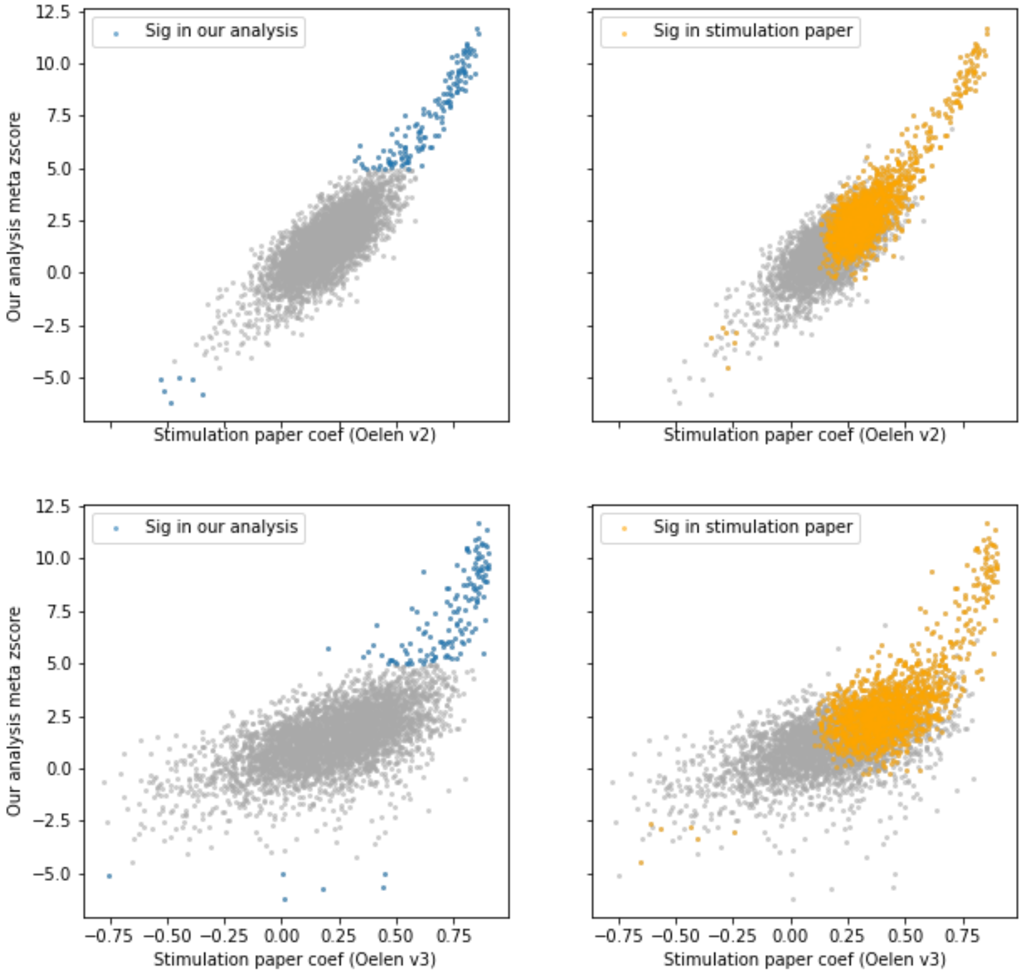

Fig. S30. *Correspondence between co-eQTLs from our study and co-eQTLs from Oelen et al. Here we compared the Z-scores for co-eQTLs identified for the SNP-eGene pair rs1131017 - RPS26. The blue dots indicate the significant co-eQTLs identified in our study, the orange dots indicate the significant co-eQTLs identified in the Oelen study. In total, Oelen study identified 1,564 co-eGenes, while we identified 148 in monocytes, and 91% of them were also identified with concordance effect direction as the top 10% significant outcomes in the Oelen study.*

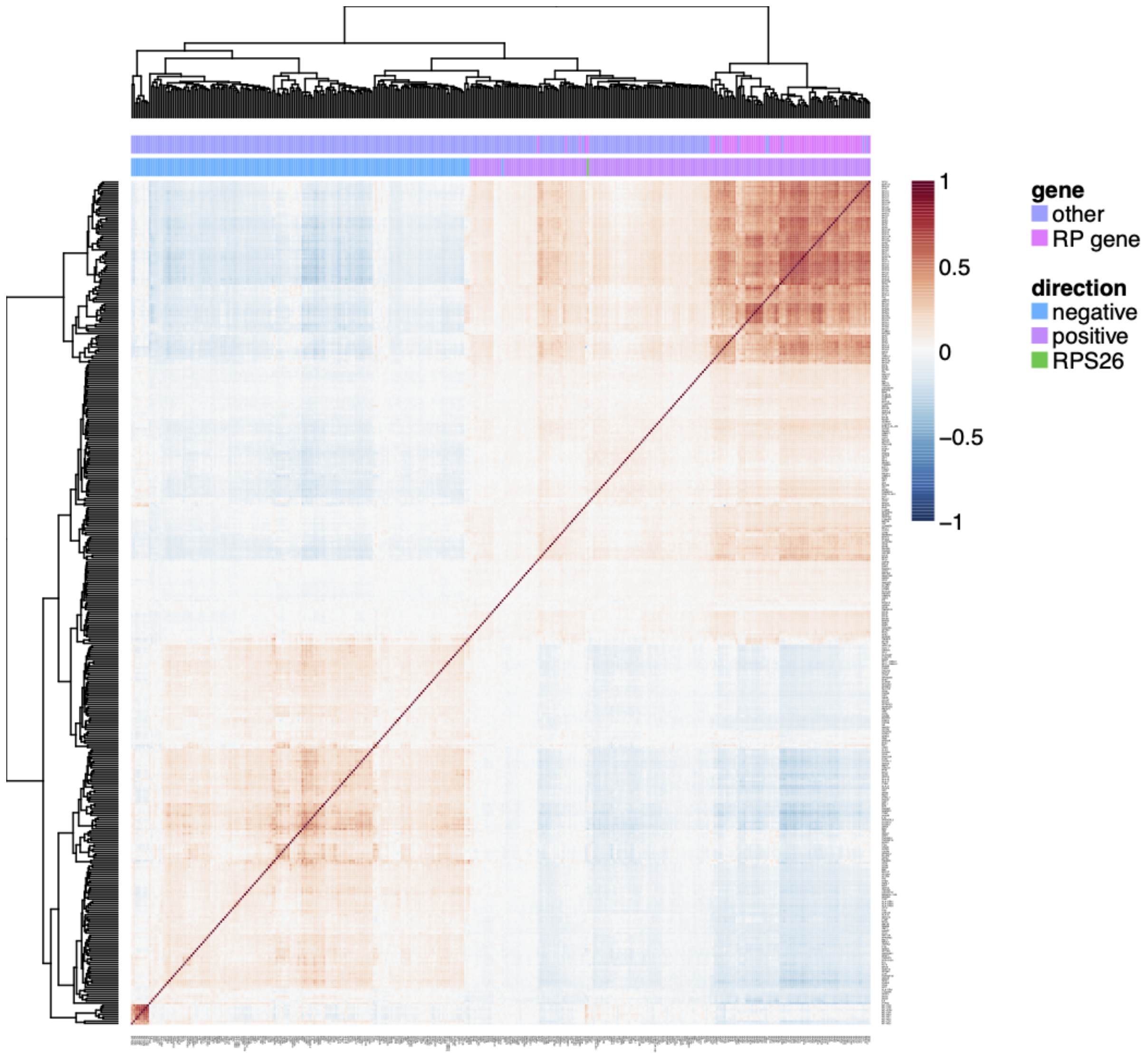

Fig. S31. *Correlation structure of rs11311017-RPS26 co-eGenes significant in CD4+T cells. The bars above the correlation heatmap show if the gene is a ribosomal gene (RP gene) and which direction of effect the corresponding co-eQTL with this co-eGene has.*

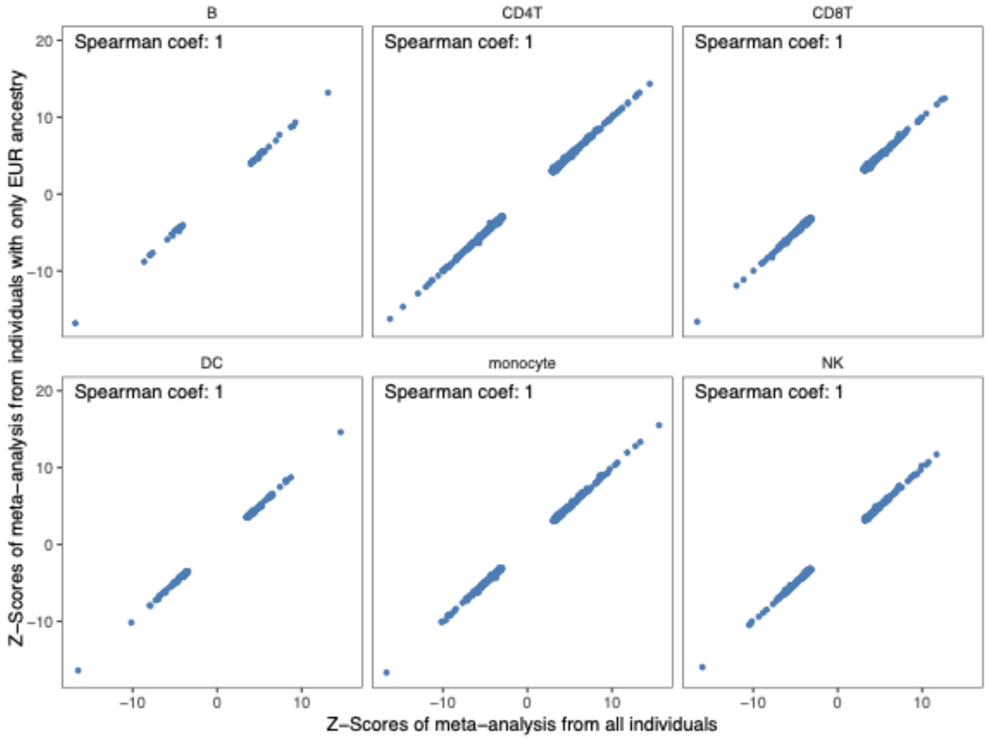

Fig. S32. *Comparison of eQTL meta-analysis Z-scores between the reported results and results generated without the two individuals of Asian ancestry background (from the van Blokland study), so that only individuals with European descent were analyzed in the second case.*

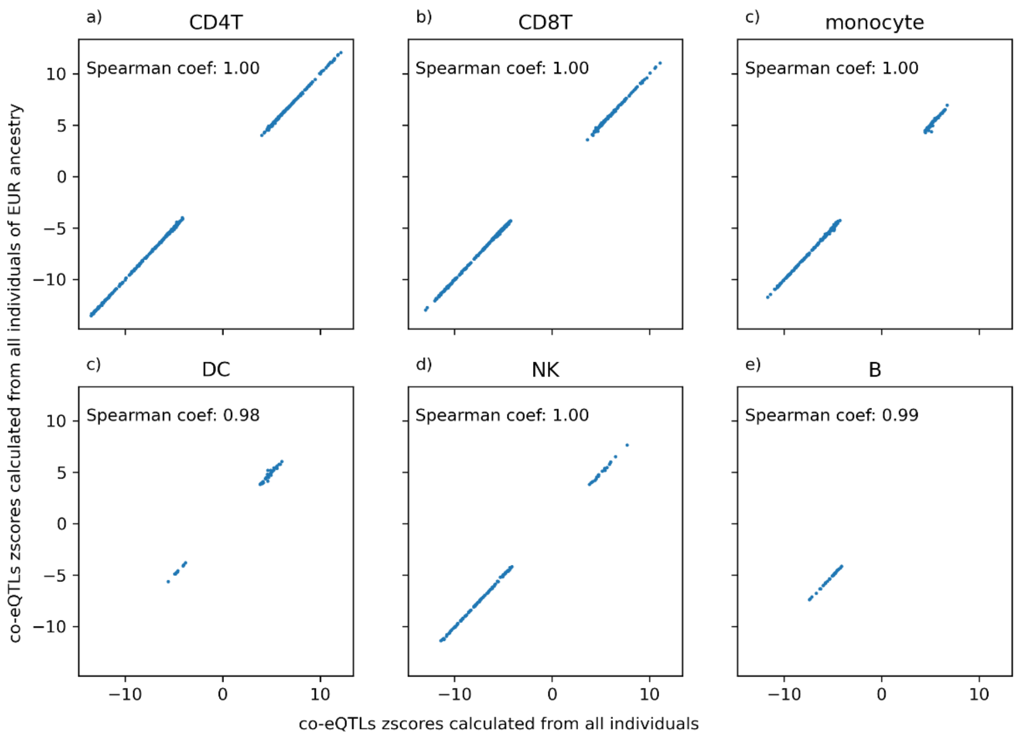

Fig. S33. *Comparison of co-eQTL meta-analysis Z-scores between the reported results and results generated without the two individuals of Asian ancestry background (from the van Blokland study), so that only individuals with European descent were analyzed in the second case.*

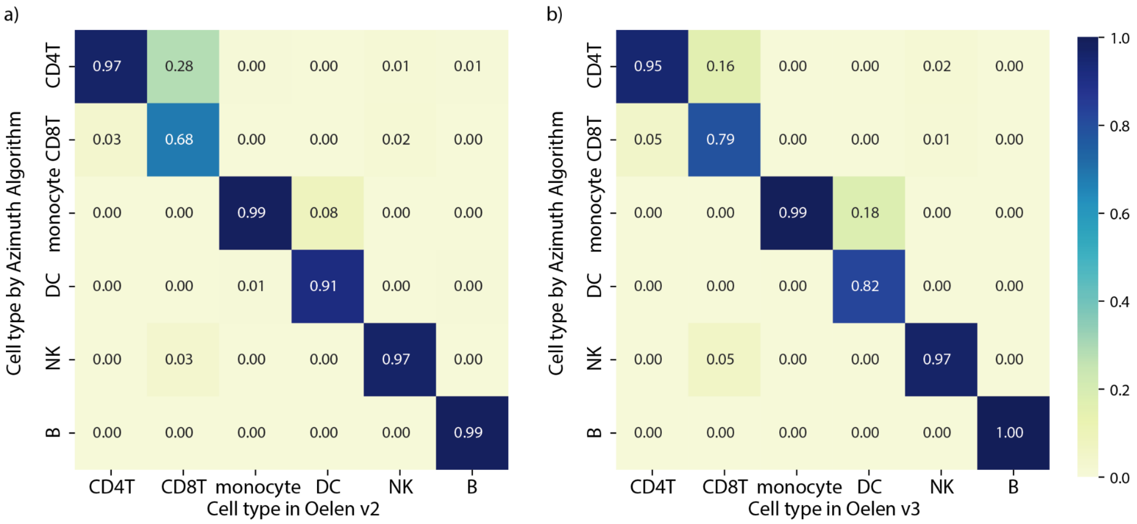

Fig. S34. *Comparison between the Azimuth cell type classification and the cell type classification provided in the original publications in* ***a)*** *Oelen v2 dataset* ***3)*** *Oelen v3 dataset.*
