# Supplementary material for: Identification of genetic variants that impact gene co-expression relationships using large-scale single-cell data
Source: Genome Biol. 2023 Apr 18;24:80. doi: 10.1186/s13059-023-02897-x (PMC10111756; doi:10.1186/s13059-023-02897-x)
Supplement: Supplementary file 3 — Additional file 3: Supplementary Note. More extensive description of meta-cell evaluation, alternative GRN construction methods and interpretation of additional co-eQTL examples. [file 13059_2023_2897_MOESM3_ESM.pdf]

Supplementary Note

MetaCell

As we observed a decline in concordance with bulk datasets for genes with more zero values (**Fig. 2c**), we looked into strategies to limit the sparseness of the data. One possibility would be to impute missing values. However, as this tends to lead to false positive associations, we decided against it (1,2). As an alternative, we merged similar cells into non-overlapping *MetaCells* (3) to reduce the zero-genes at the cell level.

Methods

We tested the *MetaCell* algorithm on the Oelen v3 dataset, Monocytes, running the algorithm separately per sample and calculating the average expression per gene over all cells assigned to the same metacell. We ran the algorithm on the full dataset, including also the stimulated conditions to increase the heterogeneity among the cells and so improving the clustering. As expected, meta-cells could be always clearly assigned to one condition, as cells from the same condition clustered together. For the evaluation shown in **Additional file 2: Fig. S3**, only the untreated meta-cells were used. Using the same approach as before, we evaluated the Spearman correlation from the meta-cells with the Spearman correlation from the BLUEPRINT dataset (4) and compared the outcome with the concordance when using the single-cell data directly. To consider especially the effect on genes with different expression levels, we grouped the gene sets thereby in different expression bins (expressed in 20%-40% of the cells, in 40%-60%, in 60%-80% and in 80%-100%) and evaluated each bin separately. We tested different parameters to reduce the granularity of the meta-cells by changing the

parameters the K and min_mc_size in the function mcell_mc_from_coclust_balanced (K between 5 and 20, min_mc_size between 3 and 10), which had however nearly no impact. As the parameter had only a small influence in the granularity of the metacells, we additionally tested meta-cells generated from Leiden clustering with a resolution of 20 (5) and performed the same evaluation.

Results

The original *MetaCell* algorithm provided too few meta-cells for a proper calculation of correlation between genes per cell type, so we implemented our own adaptation based on Leiden clustering (see Methods). We saw clearly that the fraction of zero values in the meta cells declined. However, this led to far fewer data points (metacells) per individual and could not in the end increase the concordance with bulk data from BLUEPRINT (**Additional file 2: Fig. S3**). For this reason, we did not proceed with the *MetaCell* idea, although a more extensive exploration of this approach in future work could still lead to a promising alternative strategy.

Alternative GRN construction methods

Apart from Spearman correlation, we also tested other gene regulatory construction methods, namely rho proportionality measure (6), and GRNBoost2 (7). Several other methods developed for single cell data, could not be tested, as we could not infer a reliable pseudotemporal ordering from our dataset, for which we tested RNA velocity (8) and SCORPIUS (9). We discussed the results from rho proportionality in the main manuscript, the other approaches are explained here.

Methods

GRNBoost2

We took the genes that were expressed in at least 50% of the unstimulated monocytes in the Oelen v2 data, and calculated the edge weight between the gene pairs with GRNBoost2. We repeated this analysis using monocyte data from BLUEPRINT. Then we correlated the edge weight for all gene pairs from Oelen v2 data and that from BLUEPRINT. We implemented the GRNBoost2 analysis using the tool and container provided in the benchmark study (10).

RNA velocity

For the RNA velocity estimate, we used *velocyto* (11)to get both spliced and unspliced gene count matrices followed by *scVelo* (8) for the velocity. scVelo was run on the combined set of untreated and stimulated cells, filtered for the subset of classical monocytes, using the dynamical mode and the 2000 highest variable genes.

SCORPIUS

For this analysis we selected the classical monocyte (12) to ensure that sub cell type composition will not confound the final results. We selected cells from 3 conditions: untreated condition, stimulated condition with Candida for 3 hours and stimulated condition with Candida for 24 hours. We inferred the pseudotime ordering of the cells with the scripts provided along with the study (9) with default settings.

Results

Additionally to rho proportionality and Spearman correlation (see main manuscript), we tested other GRN construction methods suggested in a recent benchmark paper (10). For algorithms that do not require time-stamps for cells, we tested the scalable top-performing method suggested in the benchmark study (10) named GRNBoost2 (7).

However, we observed poor correlation (spearman r = 0.17) between the GRN inferred from BLUEPRINT data and that from our single cell data (**Additional file 2: Fig. S5**) using GRNBoost2, which is much lower than that for Spearman correlation. Therefore, for the rest of this study we continued our analysis with Spearman correlation.

Several single-cell-specific GRN reconstruction methods had to be excluded, because they required pseudotemporal ordering of cells, which could not be reliably inferred from our dataset. RNA velocity generally does not work well in blood datasets (13). To better explore the inferred dynamics, we included the pathogen-stimulated timepoints from the Oelen dataset in our analysis (3h and 24h after pathogen stimulation), but the drastic expression changes after pathogen stimulation led to completely separate states instead of temporal trajectory (**Additional file 2: Fig. S4**). For our datasets, the inferred pseudotemporal ordering of cells is algorithm-dependent. We compared the ordering from RNA velocity (8) and a pseudotime ordering algorithm called SCORPIUS (9). Timestamps predicted by these two algorithms are poorly correlated (**Additional file 2: Fig. S4b,c**) with each other. However, the ordering from RNA velocity, which should excel in inferring cell trajectories compared to algorithms solely based on transcriptomic similarity, does not correlate well with our experimental sampling time points (**Additional file 2: Fig. S4a,b**), which makes the cell ordering results difficult to interpret.

Additional promising co-eQTL examples

Another set of co-eQTLs partly supported by our enrichment analysis is the co-eQTLs associated with rs4147638 - *SMDT1* identified in CD4+ T cells, which were found to be

enriched as for several GO terms including translation initiation and protein targeting to endoplasmic reticulum (**Additional file 9: Table S14**) and target for 5 TFs in CD4+ T cells and 37 TFs in CD8+ T cells (**Additional file 10: Table S15**). Similarly, in DCs, we identified 30 co-eGenes for the type 2 diabetes (T2D) SNP rs7935082 and eGene *MS4A7*, and these co-eGenes were enriched for T2D (**Additional file 12: Table 17**) and several endoplasmic reticulum associated GO terms (**Additional file 9: Table 14**).

References

1. Andrews TS, Hemberg M. False signals induced by single-cell imputation. F1000Research. 2019 Mar 5;7:1740.

2. Ly LH, Vingron M. Effect of imputation on gene network reconstruction from single-cell RNA-seq data. Patterns. 2021 Dec;100414.

3. Baran Y, Bercovich A, Sebe-Pedros A, Lubling Y, Giladi A, Chomsky E, et al. MetaCell: analysis of single-cell RNA-seq data using K-nn graph partitions. Genome Biol. 2019 Dec;20(1):206.

4. Chen L, Ge B, Casale FP, Vasquez L, Kwan T, Garrido-Martín D, et al. Genetic Drivers of Epigenetic and Transcriptional Variation in Human Immune Cells. Cell. 2016 Nov;167(5):1398-1414.e24.

5. Traag VA, Waltman L, van Eck NJ. From Louvain to Leiden: guaranteeing well-connected communities. Sci Rep. 2019 Dec;9(1):5233.

6. Quinn TP, Richardson MF, Lovell D, Crowley TM. propr: An R-package for Identifying Proportionally Abundant Features Using Compositional Data Analysis. Sci Rep. 2017 Nov 24;7(1):16252.

7. Moerman T, Aibar Santos S, Bravo González-Blas C, Simm J, Moreau Y, Aerts J, et al. GRNBoost2 and Arboreto: efficient and scalable inference of gene regulatory networks.

Bioinformatics. 2019 Jun 1;35(12):2159–61.

8. Bergen V, Lange M, Peidli S, Wolf FA, Theis FJ. Generalizing RNA velocity to transient cell states through dynamical modeling. Nat Biotechnol. 2020 Dec;38(12):1408–14.

9. Cannoodt R, Saelens W, Sichien D, Tavernier S, Janssens S, Guilliams M, et al. SCORPIUS improves trajectory inference and identifies novel modules in dendritic cell development [Internet]. bioRxiv; 2016 [cited 2022 Mar 29]. p. 079509. Available from: https://www.biorxiv.org/content/10.1101/079509v2

10. Pratapa A, Jalihal AP, Law JN, Bharadwaj A, Murali TM. Benchmarking algorithms for gene regulatory network inference from single-cell transcriptomic data. Nat Methods. 2020 Feb;17(2):147–54.

11. La Manno G, Soldatov R, Zeisel A, Braun E, Hochgerner H, Petukhov V, et al. RNA velocity of single cells. Nature. 2018 Aug;560(7719):494–8.

12. Villani AC, Satija R, Reynolds G, Sarkizova S, Shekhar K, Fletcher J, et al. Single-cell RNA-seq reveals new types of human blood dendritic cells, monocytes, and progenitors. Science. 2017 Apr 21;356(6335):eaah4573.

13. Bergen V, Soldatov RA, Kharchenko PV, Theis FJ. RNA velocity—current challenges and future perspectives. Mol Syst Biol. 2021 Aug;17(8).
