# Supplementary material for: Identification of genetic variants that impact gene co-expression relationships using large-scale single-cell data
Source: Genome Biol. 2023 Apr 18;24:80. doi: 10.1186/s13059-023-02897-x (PMC10111756; doi:10.1186/s13059-023-02897-x)
Supplement: Supplementary file 16 — Additional file 16. Review history. [file 13059_2023_2897_MOESM16_ESM.docx]

Review History

**First round of review**

**Reviewer 1**

**Were you able to assess all statistics in the manuscript, including the appropriateness of statistical tests used?** Yes, and I have assessed the statistics in my report.

**Comments to author:**

The article "Identification of genetic variants that impact gene co-expression relationships using large-scale single-cell data" by Li et al. presents a meta-analysis study of single cell co-expression and co-eQTL mapping, performing an interesting analysis of cell type and donor differences in co-expression patterns. Overall, the authors provide a thorough and comprehensive analysis, often providing multiple lines of evidence and benchmarking. However, many of the analysis, approaches and findings (e.g. RPS26 co-expression and co-eQTLs) are already described in the original publications of the datasets used. Moreover, although several studies were combined, the results were still limited by sample size in terms of individuals, leading the authors to use whole blood cis-eQTLs from others studies instead of performing genome-wide single cell eQTL mapping. The manuscript should expose better what is the novelty of this study either in terms of approaches or findings compared to their previous work (PMID: 35672358). Below are several comments that should be clarified by the authors.

Major remarks:

1. Given that there is a large emphasis on gene co-expression in the study, the authors should briefly describe (1) which/how many genes they studied (protein coding, lncRNA etc), (2) number/median of genes detected across studies. Moreover, the authors only indicate that they used data processed by the original publications, but a brief summary of this process would be welcome at least in the methods section.

2. In Figure 1c, the scenarios depicted are unclear and are not explained in the figure legend or main text. The figures represent a variant falling in a transcription factor motif in the promoter region of a gene. This indeed could affect the binding affinity of a TF and thus the expression of the target gene, affecting their co-expression relationship. However, it is unclear to me how this would affect the expression of other genes targeted by the same TF (scenario 2). The authors should clarify this in the main text or modify the figure.

3. As this is not usual in QTL studies, the co-eQTL approach should be better explained, including the steps undertaken (identifying cis-eQTLs based on eQTL variants, measuring gene co-expression for each individual, association tests for correlation vs genotype, filtering strategy), and the authors should explain what is the novelty compared to the co-eQTL approach in PMID: 35672358. A simple scheme in Fig 1 or 4 could help readers understand the approach undertaken. Moreover, the authors should mention in the main text how many permutations were performed for co-eQTL analysis and the significance cutoffs used to identify significant co-eQTLs (as well as the range of nominal p-values this corresponds to, at least in the methods section). In addition, even though the data comes from other publications, the authors should describe the genetic data available/used in the study.

4. Did the authors take confounding factors into account for the co-expression and eQTL analysis? Such as batch effects, genotype/expression principal components, number of cells per individual, etc. These are recommended in guidelines for single cell and bulk eQTL mapping (e.g. PMID: 34167583). Moreover, the authors assess the eQTL mapping concordance in direction and effect sizes between the eQTLs mapped in their study and BIOS, but why was this not compared also with the eQTLGen study itself? In truth, since the authors assess directly known lead eQTLs in whole blood, I expected a higher number of significant cis-eQTLs in the single cell data, could the authors comment on this?

5. Given the focus on co-eQTLs in this study, I would recommend displaying these main results (co-eQTLs mapped per cell type, # SNPs, # unique Genes, # genes pairs) in a main figure/table and not only in a supplementary table.

6. The authors should explain why colocalization analysis between nearby GWAS and eQTL signals, as performed by other studies (e.g. PMID: 35389779, PMID: 35389781), were not attempted, or explain why this is not possible in their dataset.

Minor remarks:

1. Page 7 Line 24: the authors mention 187 individuals but in the rest of the paper 173 individuals are analysed. Are some samples removed in QC? This should be described

2. Page 7: the description of the v2 and v3 datasets is confusing, from the text the authors seem to indicate that individuals are measured with both v2 and v3, whereas in fact some individuals in the studies were measured with v2 and others with v3

3. Figure 1d top right panel is unclear, what are the lines and the axis?

4. The authors found a low to moderate correlation of gene co-expression between single cell and bulk RNA datasets for the same cell types (when possible). A relatively low overlap has been found in previous studies (e.g. PMID: 27100869), even when measuring sc and bulk in the very same cell lines. However, to validate the results, the authors could measure whether consistent co-expression between bulk and single cell datasets is higher for matching cell types than for non-matching cell types. This could be shown for instance for the cell type and dataset with the most data.

5. Figure 3, it would be easier to read the comparisons if the lymphoid cell types (B, T and NT) would be next to each other. Perhaps a dendrogram on the x/y axis could be used to display the known relationship between cell lines

6. Page 15 Line 41: what do the authors mean by "bulk-like"? Averaging the gene expression values per individual across all its single cells? This should be explained. Other studies call this "pseudo-bulk"

7. Page 22 Line 24: the authors mention a few genes with high co-expression variability across individuals. Did the authors find co-eQTLs for those genes? If so, it would be good to mention

8. Page 55: the authors mention a 100kb window cutoff for lead-eQTL variants, but the variants are drawn from the eQTLGen study (Vosa et al.). While the majority of lead eQTLs are probably within this range, why did the authors decide to apply a further window cutoff and not test all of the lead-eQTLs? This window should be described in the main text. Also mention how many variants were actually tested after the window and MAF cutoff

9. It is sometimes difficult to understand which analyses are performed across individuals (e.g. single cell correlations in Fig 2 and 3) and which analyses are performed per individual (e.g. gene co-expression per individual for co-eQTL analysis)

10. Figure 4A (and related supplementary figures): why no rb values for B cells and a few other points? In the figure legend the authors also mention standard error but this is not represented in the figure

11. In addition to their co-eQTL analysis, did the authors check if the eQTL of gene1 has a significant effect directly on the co-expressed gene (gene2)?

12. The "Establishing a method to identify co-expression QTLs" section is particularly long and encopasses several topics such as the approach for (co)-eQTL mapping, identification results, comparison across cell types and bulk eQTLs under multiple conditions, sub-cell-type composition effects etc. Perhaps it would be better to split some of these findings into different sections?

13. Page 45 Line 27: The sentence "Here, the enrichment allowed us to still identify relevant TFs for further exploration" is unclear or out of place. Also note that ReMap only contains ChIP-seq data for a fraction of all human TFs (~150, whereas there are >1000 TFs in the genome). For instance, the gProfiler webserver/R-package allows to perform enrichments for Transfac

14. "Consistent with the co-eQTL overlap results, the ratio of tested co-eQTLs are generally small, ranging between 5% to 97%": how can 97% be small? Maybe provide the median instead

15. Page 33: Authors should mention in the main text which genomic regions they are looking at for the TF binding site enrichment

16. The authors performed extensive analysis of effect strength and direction concordance across cell types and provided an example of positive/negative correlation in Figure 5 depending on cell type, associated with different GO term enrichments. I wonder if the authors also analysed general patterns in their findings, such as the proportion of cases where a variant breaks the gene co-expression vs creating/increasing co-expression. For instance, is the minor allele more often breaking or making correlations? I understand that the paper already contains many analyses, so this is more of a curiosity question.

**Reviewer 2**

**Were you able to assess all statistics in the manuscript, including the appropriateness of statistical tests used?** Yes, and I have assessed the statistics in my report.

**Comments to author:**

In this paper, the authors performed a comprehensive analysis of gene co-expression patterns for different cell types inferred from single cell data across five data sets, and co-eQTL analysis to identify SNPs whose genotypes are associated with cell-type-specific correlations between certain gene pairs. The co-eQTL analysis is challenging due to the very large search space. The authors used results from previous eQTL analysis to limit the number of SNPs to be considered for each gene, i.e. one SNP per gene. They further limited the number of gene pairs to those that only include at least one eGene and having significant co-expressions in at least 10% of the subjects. This stringent filtering strategy reduced the multiple comparison burden and allowed the authors to identify statistically significant co-eQTLs for different cell types. The authors conducted GO enrichment, motif, GWAS enrichment analysis to demonstrate that the results are credible, with detailed discussion on rs1131017-RPS26 and co-eGenes.

Overall, the statistical and bioinformatics analyses presented in this paper are extensive and the results suggest that there may be novel biological insights learned from co-eQTL analysis. However, the biological interpretations are largely speculative without experimental validations and the RPS26 example is not as interesting. I have the following specific comments.

[1] A large portion of the paper is devoted to the justification of using Spearman correlations as a measure of co-expression for single cell data, and the validity of co-eQTL analysis hinges on the ability of Spearman correlation to appropriately characterize/quantify co-expression between genes. As acknowledged by the authors, single cell data are very sparse and different cells can have substantial differences in sequencing depths. Simple adoption of Spearman correlation may be confounded with sequencing depth, and highly expressed genes may be more likely found to be co-expressed with other genes. It is not clear how much this bias may contribute to the identifications of co-eGenes for the rs1131017-RPS26 example.

[2] For co-eQTL analysis, when co-expression is not significant between two genes for an individual, what's the input for the analysis? Did the authors use 0 or the calculated Spearman correlation?

[3] For replication studies using bulk samples, could the authors also use the data from the sorted cells from BLUEPRINT and ImmuNexUT, in addition to the BIOS data?

[4] For enrichment analyses on pages 34 and 35, the authors used all genes tested in the co-eQTL analysis in the respective cell type as background control. Is this an appropriate control? Can the authors use the genes that failed to be identified as co-eGenes for a given eQTL-eGene to perform a similar enrichment analysis and see whether this set may also be enriched for some GO categories?

**Authors Response**

**Point-by-point responses to the reviewers’ comments:**

**Comments raised by both reviewers**

First of all, we would like to thank both reviewers for their valuable comments and for having taken the time to review  our work. We found the comments of both reviewers very helpful and believe that the changes we have made based  on their feedback have strengthened our study. The related changes in our manuscript and our supplement are marked  with a yellow background.

A main concern for both reviewers was the novelty of our study. For this reason, we would like to first highlight the main  goals of our study and how our manuscript represents an improvement over previous publications, before we provide  the point by point response.

The previous co-eQTL study, by Oelen *et al.*, focused primarily on pathogen-dependent response-QTLs (re-QTL) (1).  For the 49 identified re-QTLs, they tested whether this response effect also manifests in co-expression, by focusing  on the response eGenes and their co-expressed genes in monocytes. In our manuscript, we improved upon the  previously proposed co-eQTL mapping strategy, expanded the search space of SNP-gene-gene triplets, and  systematically evaluated various technical aspects for co-eQTL identification, evaluation and interpretation.

In brief, we believe that our manuscript is an improvement over our previous work because: - We benchmarked co-expression metrics and improved the co-eQTL mapping strategy with better-handling  of missing values and a stricter multiple testing strategy. This new strategy allowed us to expand our  analysis from the previous targeted SNP-eGene selection to include all identified eQTLs and all genes. This  resulted in the identification of a large number of additional co-eQTL effects.

- We performed replication analyses using large bulk RNA-seq datasets, which showed high concordance of  effect sizes and directions. We employed an interaction term in the commonly used eQTL mapping  regression model as an indirect estimation of co-expression in bulk data. This shows that co-eQTLs we  identified are highly robust.

- We employed novel and extensive enrichment analyses to interpret the observed co-eQTLs. We used these  analyses to explore our hypothesis on the underlying regulatory mechanism of the observed co-eQTL  effects and their implications on cell-type-specific gene regulation in diseases. As suggested by reviewer 1,  we have now added a formal colocalization analysis to further support our findings. Specifically, in the  rs1131017-*RPS26* locus, this highlights the importance of genetic co-expression regulation for the  interpretation of the colocalizing rheumatoid arthritis GWAS association (discussed in greater detail below).

- Our manuscript empirically estimates the potential gain in the number of co-eQTLs by increasing the number  of cohorts, by including more cells per individual, and by evaluating the influence of sub-cell types. This  provides guidance and expectations for future co-eQTL studies.

- Furthermore, because we increased the sample size by meta-analyzing three studies, we were able to  contrast co-eQTLs in all 6 major immune cell types in this manuscript, whereas in our previous study we  focused solely on the most frequent cell type.

**Novelty of *RPS26* findings**

Altogether, by extending our previous studies on these aspects, we gained valuable new insights in cell-type-specific  regulation in disease mechanisms. For example, we identified a set of genes that co-express with the gene *RPS26* and whose co-expression patterns are associated with the SNP rs1131017. The SNP rs1131017 is in high LD with  rs773125 (R^2^ = 0.879), which has previously been associated with rheumatoid arthritis in several large scale GWAS  studies (2,3). A recent TWAS study (4) utilized the GWAS summary statistics (2) and identified two significant  genes (*SOUX* and *RPS26*) in the locus. Additionally, the SNP rs1131017 was found as the leading SNP for a *trans* eQTL locus specifically in T cells (5). Inspired by these observations, a recent paper (6) sought to elucidate the role  of *RPS26* in T lymphocytes. They examined a T-cell-specific *RPS26* knockout mouse model and reported that

ablation of *RPS26* in T cells impairs peripheral T-cell homeostasis and leads to T-cell developmental arrest in the  thymus. Despite the great interest in this locus and the role of *RPS26* in lymphocytes, the associated pathways and  biological processes that underlie the rheumatoid arthritis GWAS signal are still largely unknown.

Two previous studies have described co-eQTLs in the RPS26 locus (Oelen *et al.* (1) , van der Wijst *et al.* (7)), and  we have already highlighted the major differences between these studies and our current manuscript. In the case of  the *RPS26* locus, our current manuscript improves the interpretability of the locus, for instance by identifying co eQTLs in cell types that were not assessed in the previous studies. More specifically, we identified an additional  group of T-cell activation genes that can directly be used for the interpretation of the autoimmune disease  associations. These new findings are due to our sample size and increased power, our updated co-eQTL mapping  strategy and our comprehensive cell-type comparison. Additionally our colocalization analysis showed the high  posterior probabilities for a shared signal between *RPS26* co-eQTLs and RA for this cell type, especially for co eGenes associated with T cell activation, suggesting T cell specific co-expression regulation from this locus that was  not described before.

We added a section into the **Results** in the manuscript to clarify the interpretation of the rs1131017-*RPS26:*

*“GWAS enrichment analysis showed enrichment for several different blood cell counts in all cell types. However, in  CD4+ T cells and CD8+ T cells we additionally observed specific enrichment for the immune-mediated diseases  rheumatoid arthritis (RA), Crohn’s disease (CD), multiple sclerosis (MS) and hay fever. This shows the relevance of T  cell associated co-eGenes for these diseases (****Supplementary Table 17****). We confirmed with colocalization  analyses that there is very likely a shared signal between the eQTL and co-eQTLs signals and the GWAS signals  from RA and asthma (****Supplementary Table 18,19****). Among the co-Genes with the highest colocalization posterior  probability (PP4>0.9), 6 out of 41 were again associated with lymphocyte activation. Interestingly, several studies  have highlighted a connection of RPS26 with T cell activation and survival (35), and the associated co-eQTL SNP  rs1131017 is associated with the enriched immune-mediated diseases (RA, CD, MS, hay fever).”*

We also updated the **Discussion** to place the rs1131017-*RPS26* findings in a broader context:

“*Another interesting aspect of the rs1131017–RPS26 example is that we revealed a potential mechanism for a  previously described GWAS signal by showing cell-type-specific genetic regulation of a multi-functional gene. The  SNP rs1131017 is in high LD with rs773125 (R2 = 0.879), which has previously been associated with rheumatoid  arthritis in several large scale GWAS studies (36,37). A recent TWAS study (52) utilized the GWAS summary  statistics (36) and RPS26 was identified as one of the significant genes in the locus. Additionally, the SNP rs1131017  was found as the leading SNP for a trans-eQTL locus specifically in T cells (53). Inspired by these observations, a  recent paper (35) sought to elucidate the role of RPS26 in T lymphocytes. They examined a T-cell-specific RPS26  knockout mouse model and reported that ablation of RPS26 in T cells impairs peripheral T-cell homeostasis and  leads to T-cell developmental arrest in the thymus. Despite the great interest in this locus and the role of RPS26 in  lymphocytes, the associated pathways and biological processes that underlie the rheumatoid arthritis GWAS signal  are still largely unknown. By comparing T cells and monocytes, we identified that RPS26 may be involved in two  distinct biological functions. Interestingly, these two distinct functional co-eQTL clusters are characterized by opposite  effect directions. Moreover, while RPS26 showed enough variation to be picked up as an eQTL effect, it did not show  high correlation with either gene cluster (****Supplementary Figure 31****), which may be why understanding its role in  multiple functions has been challenging up to now (35). We envision that more multi-functional eGenes could operate  in such a cell-type-specific manner, with variation in expression that could be explained as the downstream  consequences of many other conserved or highly co-expressed gene clusters, and this understanding could assist in  interpreting GWAS signals. We also observed that different eGenes could have shared upstream genes/pathways as  we identified four common immune-related co-eGenes associated with rs393727–RNASET2 and rs1131017–RPS26,  and both SNPs were in LD with immune diseases (T1D and CD), suggesting a shared upstream process for these  two eQTL effects. By providing cell-type-specific gene regulation backgrounds through co-eQTLs, we expect more  eQTLs and GWAS signals to be explained in the relevant cell type via future large-scale co-eQTL studies.*”

Additionally, we have updated the **Introduction** section in order to make the main differences to previous  publications more clear.

*“In this work, we studied the genetic regulation of gene co-expression by conducting the largest-to-date co-eQTL  meta-analysis in 173 peripheral blood mononuclear cell (PBMC) scRNA-seq samples. Previous studies focused on a  small set of SNP-gene-gene triplets and specific cell types, limiting the number of identified co-eQTL. Compared to  previous studies, we have increased the sample size, improved the co-eQTL mapping strategy and have applied  comprehensive interpretation strategies. This enabled a larger search space of SNP-gene-gene triplets, consequently  highlighting the cell type specificity of gene-gene regulation underlying GWAS signals. To make this possible, we first  benchmarked various GRN construction methods and compared the obtained co-expression patterns in our scRNA seq data to two bulk RNA-seq datasets and a CRISPR-coupled scRNA-seq screen knockout dataset. We then  studied the effects of cell-type and inter-individual differences in gene co-expression networks by reconstructing  personalized and cell-type-specific networks. We subsequently developed a robust co-eQTL mapping strategy with a  novel filtering approach and a customized permutation-based multiple testing procedure to deal with the correlation  structure in the co-expression networks. With the improved strategy, we performed a co-eQTL meta-analysis using  data from three different scRNA-seq studies. We provided a comprehensive analysis of the different factors that  affect the quality and quantity of co-eQTLs, including the number of cells, gene expression levels and filtering  strategy. We then studied which biological processes and genes are regulated by the identified co-eQTLs by  performing different enrichment analyses and exploring common biological functions, transcription factor (TF) binding  and disease associations to pinpoint potential direct regulators of the co-eQTL genes. In summary, our results  suggest that the combination of a robust method and a large sample size is crucial for identification of genetic  variants that affect co-expression networks.”*

We acknowledge that this study is limited in terms of statistical power, which in turn limits the number of detected co eQTLs (as mentioned in the **Discussion**). Although this is a general issue for all current single-cell datasets, this is  likely to be overcome in the near future, especially with large consortia such as sc-eQTLgen (8). Our work can be  considered a jump-start towards these future studies, because we empirically examined the influence of technical  factors on co-expression and co-eQTL analysis, which will be particularly helpful when scaling up. For instance, we  observed that the number of co-eQTLs increases linearly with the number of cells per individual and the number of  individuals (**Figure 4**). As such, we expect that the number of detected co-eQTLs will quickly rise in the future due to  the quickly increasing sample size and number of available single-cell datasets.

**Reviewer #1:**

**The article "Identification of genetic variants that impact gene co-expression relationships using large-scale  single-cell data" by Li et al. presents a meta-analysis study of single cell co-expression and co-eQTL  mapping, performing an interesting analysis of cell type and donor differences in co-expression patterns.  Overall, the authors provide a thorough and comprehensive analysis, often providing multiple lines of  evidence and benchmarking. However, many of the analysis, approaches and findings (e.g. RPS26 co expression and co-eQTLs) are already described in the original publications of the datasets used. Moreover,  although several studies were combined, the results were still limited by sample size in terms of individuals,  leading the authors to use whole blood cis-eQTLs from others studies instead of performing genome-wide  single cell eQTL mapping. The manuscript should expose better what is the novelty of this study either in  terms of approaches or findings compared to their previous work (PMID: 35672358). Below are several  comments that should be clarified by the authors.**

We thank the reviewer for the helpful comments and suggestions. The two general concerns on the novelty of our  study and the power issue are discussed in the **Comments raised by both reviewers** above in detail. We also  updated the manuscript to highlight our improved co-eQTL mapping strategy, enabling the discovery of a potential  cell-type-specific genetic regulation mechanism underlying one previously identified GWAS hit.

Furthermore, we fully acknowledge the fact that this study is still of limited power for both eQTL and co-eQTL  discovery, but do want to highlight our effort to establish a robust strategy for future studies. We elaborated on this

topic together with the confounding factor discussion in the response for **major remark 4** and again in the  **Comments raised by both reviewers** section.

Major remarks:

**1. Given that there is a large emphasis on gene co-expression in the study, the authors should briefly  describe (1) which/how many genes they studied (protein coding, lncRNA etc), (2) number/median of genes  detected across studies. Moreover, the authors only indicate that they used data processed by the original  publications, but a brief summary of this process would be welcome at least in the methods section.**

We updated the description in the **Methods** section:

*“For each study, blood samples were collected from individuals into EDTA-vacutainers (BD), after which PBMCs were  isolated using Cell Preparation Tubes with sodium heparin (BD) and were cryopreserved in RPMI1640 containing  40% FCS and 10% DMSO until further use. For each individual, cryopreserved PBMCs were thawed and 6-8 donors  were multiplexed in individual sample batches that were then loaded on a 10X Chromium controller. Subsequent  libraries were then generated for each sample batch using 10x Genomics single cell 3’ reagents (v2 and v3). Libraries  were sequenced using 150PE sequencing on the Illumina NovaSeq 6000 at BGI (Hong Kong). For dataset processing we used CellRanger (v1.3 for van der Wijst and v3.0.2 for Oelen and van Blokland) for  scRNA-seq alignment, Demuxlet for demultiplexing, and Seurat (4.1.0) for quality control (QC) and Azimuth v0.4.6  (with the default reference from (57)) for cell type classification. Cells with a high percentage of mitochondrial genes  (5% in the van der Wijst study, 8% and 15% in v2 and v3 chemistries respectively in both Oelen and van Blokland  studies), number of genes expressed per cell (more than 3,500 genes/cell in van der Wijst study and less than 200  genes/cell in Oelen and van Blokland studies) and doublets (Demuxlet for van der Wijst and SoupOrCell for Oelen  and van Blokland) were excluded during these QC steps. The Oelen dataset also contains cells stimulated with  different pathogens, but we only included the unstimulated cells in this analysis to improve comparison with the other  datasets. For the van Blokland dataset, we included the data from the time point 6–8 weeks after the individual was  admitted to the hospital for myocardial infarction, again to improve comparison across datasets. More details on  dataset processing are provided in the original publications ((10,11,19)).”*

Additionally, we added extended information about the dataset details to **Supplementary Table 1a**: the median  number of expressed genes per cell, the percentage of protein coding genes and the percentage of lncRNA genes.  See below:

| **Data set (version of chemistry)** | **Number**  **of donors** | **Average**  **Number of cells per**  **donor** | **Number of counts per cell** | **Number of counts per person** | **Total**  **number of cells per**  **cohort** | **Total**  **number of reads per cohort** | **Number**  **of genes**  **detected in cohort*** | **Median**  **number of genes per cell per**  **cohort** | **% of**  **Protein coding**  **genes** | **% Of**  **lncRNA genes** |
| --- | --- | --- | --- | --- | --- | --- | --- | --- | --- | --- |
| van Blokland (v3) | 14 | 751 | 4,778 | 3588278 | 10514 | 50235892 | 19860 | 314.5 | 78.2% | 20.5% |
| van Blokland (v2) | 24 | 907 | 2,307 | 2092449 | 21768 | 50218776 | 17593 | 229 | 82.6% | 16.2% |
| Oelen (v3) | 32 | 1163 | 6,519 | 7581597 | 37216 | 242611104 | 21491 | 891 | 74.5% | 24.2% |
| van der Wijst (v2) | 45 | 560 | 3,735 | 2091600 | 25200 | 94122000 | 22229 | 258 | 79.7% | 18.9% |
| Oelen (v2) | 72 | 1082 | 2,820 | 3051240 | 77904 | 219689280 | 19259 | 828 | 77.8% | 20.9% |

* number/median of genes detected across studies. Gene is detected if there are at least 3 cells within the  cohort which have this gene present.

**2. In Figure 1c, the scenarios depicted are unclear and are not explained in the figure legend or main text.  The figures represent a variant falling in a transcription factor motif in the promoter region of a gene. This  indeed could affect the binding affinity of a TF and thus the expression of the target gene, affecting their co expression relationship. However, it is unclear to me how this would affect the expression of other genes  targeted by the same TF (scenario 2). The authors should clarify this in the main text or modify the figure.**

We thank the reviewer for the suggestions and updated **Figure 1** and the manuscript for a clearer description of our  Scenario 2. The expression of the other gene targeted by the same TF (the yellow gene in the scenario 2) is not  affected by the genotype of the co-eQTL SNP. However, if there is a co-eQTL between the TF and the eGene and  additionally there is a strong correlation between the other TF target gene (yellow) and the TF, then the indirect  relationship between the second TF target gene and the eGene will also be visible as a co-eQTL. This is because the  Spearman correlation can not distinguish between direct and indirect interactions. We updated the **Results** section  as follows:

*“For the co-eQTL interpretation, we considered different scenarios that can lead to detection of a co-eQTL. One is  that the genetic variant changes the binding affinity of a TF and thus the regulation of its target gene, which would  cause a co-eQTL between the variant, the TF and the target gene (****Figure 1c, Scenario 1****). However, a co-eQTL will  also be observed indirectly for all genes that have a strong correlation with this TF (****Figure 1c, Scenario 2****): a gene  correlated with the TF will also be correlated with the eGene and this correlation strength will also depend on the  genotype because of the indirect association via the TF.”*

**New main Figure 1.** Study overview.

a) Overview of the three PBMC scRNA-seq studies used in our study. The studies, the version of the used chemistry  for data generation (version 2, referred as v2, and version 3, referred as v3), number of individuals involved (indicated  as the number in the parenthesis), and relative composition of the major blood cell types used in this study.  b) Co-expression benchmarking scheme. We first benchmarked co-expression patterns among the three scRNA-seq  studies and compared them to co-expression patterns in different bulk datasets. As an additional external validation,  we benchmarked both the scRNA-seq and bulk co-expression patterns with a CRISPR knockout dataset. After  benchmarking, we evaluated differences in co-expression patterns among cell types and among individuals within a  cell type.

c) Co-expression QTL (co-eQTL) mapping. Building on the benchmarked co-expression pattern, we developed a  novel strategy to identify co-eQTLs (genetic variants changing co-expression). Part of the strategy is a strict filtering  of tested SNP–eGene–co-eGene triplets, where the SNP is required to be an eQTL for one of the genes and the  genes show significant correlation in at least a certain number of individuals.

d) Co-expression QTL (co-eQTL) insights. We first check if the SNP we tested has been identified in previous GWAS  studies, or it is in high LD with GWAS variants. Then we check the group of co-eGenes on what pathways and traits  and TF binding sites they are enriched for. We hypothesize two possible scenarios of the underlying biological  mechanism for the identified co-eQTLs. Scenario 1 portrays a direct regulatory association between the co-eGene  and the eGene through the genetic variants that changes the binding affinity. Scenario 2 portrays an indirect  association between the co-eGene and the eGene. That is, the co-eGene is co-expressed with the TF that regulates  the expression level of the eGene.

**3. As this is not usual in QTL studies, the co-eQTL approach should be better explained, including the steps  undertaken (identifying cis-eQTLs based on eQTL variants, measuring gene co-expression for each  individual, association tests for correlation vs genotype, filtering strategy), and the authors should explain  what is the novelty compared to the co-eQTL approach in PMID: 35672358. A simple scheme in Fig 1 or 4  could help readers understand the approach undertaken. Moreover, the authors should mention in the main  text how many permutations were performed for co-eQTL analysis and the significance cutoffs used to identify significant co-eQTLs (as well as the range of nominal p-values this corresponds to, at least in the  methods section). In addition, even though the data comes from other publications, the authors should  describe the genetic data available/used in the study.**

We thank the reviewer for the constructive suggestions and comments. We updated the text and added schematic  figures for better illustration of the co-eQTL mapping strategy (**New main Figure 1**, see major remark 2), we have  provided more statistical details in the **Methods** section, and have added **Supplementary Table 1b** for more detailed  information about the used genetic datasets.

1) For a clearer explanation of the novelty of the proposed co-eQTL mapping strategy compared to previous study,  we updated the text in the co-eQTL identification in the **Results** section as follows:

*“In this study, we aimed to define a generally applicable co-eQTL mapping strategy that yields a large number of  highly confident co-eQTLs, which, compared to our previous studies, represents: 1) a rigorous assessment and  comparison of different analysis strategies, 2) an updated gene and gene pair filtering strategy, 3) an improved  computational pipeline for better handling the missing values, and 4) a customized stricter multiple testing strategy.”* 2) For a clearer illustration of the co-eQTL identification strategy, we updated **Figure 1c)** with a schematic figure  (**New main Figure 1**, see major remark 2).

3) Also, we updated the **Results** section by now giving more details on the number of permutations applied and the  significance cutoff:

*“Therefore, we applied a customized permutation and multiple testing correction strategy per SNP–eGene pair based  on FastQTL. We used 100 permutations to adjust nominal p-values and determined significance using Benjamini Hochberg correction over all SNP-eGene pairs. We considered those with FDR<=0.05 as significant (see Methods for  details).”*

We have added a detailed description of this procedure to the **Methods** section:

*“To account for the correlation structure between co-eQTL SNP-eGene-co-eGene triplets, we modified and applied  the permutation-based multiple testing correction strategy from FastQTL (29), implementing the method as follows.  For each triplet, we performed 100 permutations. Then, for each SNP–eGene pair, we determined the lowest non-*

*permuted nominal p-value and the lowest permuted p-value per permutation over all the genes (co-eGene) tested for  the SNP–eGene pair, resulting in a list of the 100 lowest permuted p-values per SNP–eGene pair. For each SNP– eGene pair, we then fitted a beta-distribution over these 100 permuted lowest p-values, which enabled us to  subsequently establish the empirical p-value for the lowest non-permuted p-value. Through this procedure we  ensured that under the null test statistic each SNP-eGene pair has a uniform p-value distribution. Finally, over the  empirical p-values for all SNP–eGene pairs, we calculated Benjamini-Hochberg FDR. Those SNP-eGene pairs with a  FDR<=0.05 were deemed significant.*

*For each SNP–eGene pair, we also derived a p-value cutoff that indicates which of the co-eGenes are significant for  that SNP–eGene pair via the following steps. After determining the FDR for all SNP–eGene pairs, we determined the  empirical p-values that are closest to FDR = 0.05. Using the beta distributions for each SNP–eGene pair, we then  determined its nominal p-value threshold. All co-eGenes with a nominal p-value lower than the corresponding p-value  threshold for that SNP–eGene pair were considered significant. ”*

4) We added the range of nominal p-values in the **Methods** section has follows:

*“The nominal p-value thresholds range from 1.75e-41 to 1.34e-4.”*

5) We updated our description on the participants in the **Methods** section and added **Supplementary Table 1b** for  genetic data information as follows:

*“Further specifications can be found in Supplementary Table 1a) and 1b), and the respective manuscripts.  Briefly, the Oelen dataset and the van der Wijst dataset included general population participants with European  ancestry background, whose ages range from 20 to 79. The van Blokland dataset included patients 6-8 weeks  post ST-elevated myocardial infarction, whose ages range from 43 to 95, the majority of whom are of European  ancestry background.”*

We elaborated on the potential batch effects caused by different genetic ancestry backgrounds for two individuals in  the Van Blokland cohort in the answer for the next question (**major remark 4**).

***Supplementary Table 1b Description of genetic data used in the study***

| Dataset | Sample  Size | Genotyping  Method | Ancestry | Healthy /  Disease Status | Age  Range | Gender  Ratio | Imputation |
| --- | --- | --- | --- | --- | --- | --- | --- |
| Oelen  data | 120 | HumanCytoSN P-12 BeadChip | European  background  individuals of the  northern  Netherlands  population | General  population | 20-79 | 67 Female  and 53  Male | Imputed to  HRC  reference  panel |
| Van der  WIjst  data | 45 | HumanCytoSN P-12 BeadChip | European  background  individuals of the  northern  Netherlands  population | General  population | 20-79 | 21 Female  and 24  Male | Imputed to  HRC  reference  panel |
| Van  Blokland  data | 38 | Infinium Global  Screening  Array multi  disease  version 1 | Mostly European  background  except for two  individuals with  Asian ancestry  background | 6-8 weeks post  ST-elevated  myocardial  infarction | 43-95 | 6 Female  and 32  male | Imputed to  1000G  Phase 3 |

**4. Did the authors take confounding factors into account for the co-expression and eQTL analysis? Such as  batch effects, genotype/expression principal components, number of cells per individual, etc. These are**

**recommended in guidelines for single cell and bulk eQTL mapping (e.g. PMID: 34167583). Moreover, the  authors assess the eQTL mapping concordance in direction and effect sizes between the eQTLs mapped in  their study and BIOS, but why was this not compared also with the eQTLGen study itself? In truth, since the  authors assess directly known lead eQTLs in whole blood, I expected a higher number of significant cis eQTLs in the single cell data, could the authors comment on this?**

We thank the reviewer for this important question. Confounders and batch effects could affect our results on multiple  levels. We will explain for each point how we guarded against it in the following:

1) Batch effects might affect the calculation of person specific co-expression between pairs of genes. If cells of an  individual were distributed across multiple runs - each with a batch specific offset - this would lead to inflated  correlations. By design of our experiments, we ruled this out by measuring all cells from one sample always  together in the same batch.

2) Different numbers of cells per person affect the magnitude of the estimated correlation coefficients. We  accounted for this effect by transforming correlation coefficients to Z-scores (a transformation that depends on  the number of cells) as input to the co-eQTL analysis instead of the coefficients directly. We added description  for this additional step in the **Methods** section as follows:

“*We additionally transformed the Spearman correlation into a t-statistics with the sample size being the number  of cells in one individual, and subsequently normalized the t-statistics to a z-statistics that follows a normal  distribution with mean of zero and variance of one.*”

3) Batch effects and hidden confounders might introduce false positives in the eQTL or co-eQTL study. This  scenario could occur if a hidden confounder followed the exact same distribution over the samples as the  genotypes of a locus. Since loci across the genome are generally independent from each other, this scenario is  expected to at most affect a single individual locus (LD region) and not drive the overall results.

4) Additionally, since hidden confounders are expected to differ between different experiments, we expect that the  meta-analysis of 5 datasets for eQTL and co-eQTL mapping significantly reduces the dataset-specific  confounding effects, and therefore, the great majority of results not to be driven by confounders. Our replication  results support this view. We showed that not only the concordance of effect sizes and effect directions between  BIOS and our study is very high (**Figure 4b**), but also between the different single-cell datasets of our meta analysis (**Supplementary Figure 19**).

5) The main reason for correcting for confounders is to increase the discovery power (to avoid false negatives) as  shown in the initial PEER manuscript (9). Therefore, in our manuscript there is an increased risk of false  negatives when not accounting for hidden confounders, which is expected to lead to more conservative results.

For the concern of population structure, we apologize for our previous neglection of 2 individuals that were not of  European ancestry. To check the influence of the ancestry differences on the identified eQTLs and co-eQTLs, we  compared the Z-scores of the significant eQTLs and co-eQTLs to the Z-scores calculated excluding the 2 non European ancestry individuals. As shown in the figures below, we observed a Spearman correlation of nearly 1 for  eQTLs and co-eQTLs identified in all the cell types when correlating the meta-analysis Z-scores. Therefore, we  conclude that the used eQTLs and reported co-eQTLs were not due to ancestry differences in the data. We also  added the following figures as **Supplementary Figure 32,33** in the manuscript.

**Supplementary Figure 32:** Comparison of eQTL meta-analysis Z-scores between the reported results and results  generated without the two individuals of Asian ancestry background (from the van Blokland study), so that only  individuals with European descent were analyzed in the second case.

**Supplementary Figure 33:** Comparison of co-eQTL meta-analysis Z-scores between the reported results and results  generated without the two individuals of Asian ancestry background (from the van Blokland study), so that only  individuals with European descent were analyzed in the second case.

We have updated the **Methods** section:

“*In the van Blokland data, there are two individuals of Asian ancestry background. We examined how much the meta analysis Z-scores of the identified eQTLs and co-eQTLs would be changed by excluding the two individuals, but did  not observe a large discrepancy (****Supplementary Figure 32, 33****).*”

For the second part of the question: we replicated our single-cell co-eQTLs in the BIOS dataset, as the replication  requires that interaction effects are calculated in the bulk dataset (see **Methods** section of the manuscript). This is a  type of analysis that was not performed by the eQTLGen consortium. Since this consortium performed a meta analysis using summary statistics and interaction analysis would require access to raw data, this is an analysis that  would require re-analysis by each of the >30 participating cohorts, which we considered outside the scope of this  manuscript. As such, we don’t have interaction QTL results available from the eQTLGen consortium. Since  interaction analysis requires a large sample size, we opted to focus on the BIOS dataset, for which we do have raw  data available for a large number of samples. When we analyzed the interaction QTLs for the BIOS dataset, we found  high replication rates (r_b_ values between 0.30 to 0.61) between the Z scores from the interaction analysis in bulk and  the Z scores from the direct single-cell co-eQTLs. These high replication rates show that the current sample size is  sufficient to reliably detect co-eQTLs.

Nevertheless, the eQTL Z-scores can be compared between the bulk eQTLGen and our single-cell analysis. The  following scatter plots show a very high concordance for *cis*-eQTL effects in both studies. The concordant allelic  direction ratio is calculated as the number of significant eQTLs of concordant allelic direction with eQTLGen divided  by the number of total significant eQTLs in the single cell datasets. The blue points are the significant eQTLs in the  single-cell data and the gray points are the insignificant SNP-eGene pairs tested.

For the third part of the question: it is true that the number of eQTLs in our study is lower compared to the numbers  presented in bulk eQTL studies. The number is, however, well in line with what to expect from single-cell studies,  which, in contrast to bulk studies, currently have far smaller sample sizes and sparser expression data. Both are  technical issues whose consequences are expected to be overcome in the near future, for example through efforts of

the sc-eQTLGen consortium. We added this now into the **Discussion** to clarify this:

*“We showed that the number of significantly detected co-eQTLs can be greatly increased by either increasing the  number of individuals or by increasing the number of cells per individual (Figure 4c, d). The limited power is also  visible in the number of identified single cell eQTLs, which is well in line with other single cell studies of similar  sample size (11), but lower compared to large bulk eQTL studies (2). We believe that future larger single-cell  datasets such as two very recent studies (49,50) and the sc-eQTLGen consortium (51) will improve statistical power  to identify more robust eQTLs and co-eQTLs*.*”*

To show that the number of eQTLs we found is in the expected range for a single-cell study, we compared the  number of eQTLs in the Oelen publication and the current manuscript. In the table below, we provide this  comparison. Additionally, the Oelen paper showed nicely that a confinement to SNPs identified in eQTLGen  increases the power to identify eQTLs (column two vs column three).

| Cell type | Number of eQTLs found in  Oelen study confined by the top  SNP per gene from eQTLGen | Number of eQTLs found  in Oelen study  (unconfined) | Number of eQTLs found in Li  study confined by the top SNP  per gene from eQTLGen |
| --- | --- | --- | --- |
| Mono | 560 | 319 | 615 |
| CD4T | 610 | 406 | 903 |
| CD8T | 420 | 219 | 518 |

| NK | 289 | 196 | 369 |
| --- | --- | --- | --- |
| DC | 145 | 91 | 143 |
| B | 50 | 30 | 57 |

This table shows that the number of eQTLs is comparable or higher for each cell type after performing our meta analysis. With the meta-analysis of 4 datasets, we increased the number of tested variants from 10,477 (Oelen et al.  (1)) to 14,009. Additionally, compared to the Oelen study, we used a more recently published cell-type annotation  method, called Azimuth (10). We have shown that the predicted cell types are highly similar (**Supplementary Figure  34**).

We have updated the **Methods** section as follows:

“*Due to the limited sample size, we chose to perform a constrained eQTL analysis rather than a genome-wide  analysis. We have shown previously that this confined eQTL mapping strategy can greatly reduce the multiple testing  burden and identify additional significant eQTLs. In our eQTL meta-analysis, we have increased the number of  significant eQTLs from 560 (11) to 615.*”

**5. Given the focus on co-eQTLs in this study, I would recommend displaying these main results (co-eQTLs  mapped per cell type, # SNPs, # unique Genes, # genes pairs) in a main figure/table and not only in a  supplementary table.**

Following the reviewer’s suggestions, we added a **Main Table 1** to the manuscript, which shows these numbers  (originally shown in Supplementary Table 4).

| Cell type | # co-eQTLs | # unique co-eQTL  SNPs | # unique co-eQTL  gene pairs | # unique co-eQTL genes | # tests |
| --- | --- | --- | --- | --- | --- |
| CD4+ T | 500 | 30 | 500 | 420 | 179,841 |
| CD8+ T | 420 | 22 | 420 | 322 | 73,017 |
| monocyte | 281 | 24 | 280 | 235 | 304,707 |
| DC | 58 | 9 | 58 | 62 | 41,655 |
| NK | 123 | 10 | 123 | 121 | 25,998 |
| B | 35 | 1 | 35 | 36 | 2,936 |

*Main Table 1. Number of significant co-eQTLs identified with our mapping strategy per cell type.*

**6. The authors should explain why colocalization analysis between nearby GWAS and eQTL signals, as  performed by other studies (e.g. PMID: 35389779, PMID: 35389781), were not attempted, or explain why this is  not possible in their dataset.**

We thank the reviewer for this suggestion and now added a colocalization analysis for selected loci of interest. For  the colocalization analysis, all available SNPs in a genomic locus need to be tested. As we did a preselection for  eQTL SNPs to reduce the multiple-testing burden in our initial study, we limited the colocalization analysis to the  genomic loci around 5 interesting co-eQTLs (each with a SNP-eGene pair and multiple co-eGenes), which were  connected with GWAS traits in the enrichment analyses presented in the manuscript. The selected five co-eQTL loci  (each with multiple significant co-eGenes) contained the following eGenes: *RPS26, HLA-DQA, RNASET2, SMDT1* and *TMEM176A*. For those eGenes, we reran the eQTL and co-eQTL analysis for all SNPs in a 1 megabase (MB)  window around the transcription start site (TSS) of the eGene. We then performed the colocalization analysis with  GWAS traits for which we found associations in the enrichment analysis. Besides doing the colocalization analysis for  the eQTL signal in all cell types we also ran the colocalization analysis on the co-eQTL signal of all significant co eGenes of those eGenes. We compared the signal for seven GWAS traits: Type 1 diabetes, Rheumatoid Arthritis

(RA), Crohn’s Disease (CD), Multiple Sclerosis (MS), asthma, Inflammatory Bowel Disease (IBD) and white blood cell  count (complete procedure described in **Methods** section).

We have added this analysis to the **Results** section as follows:

*“In the following section, we highlight some examples of how these co-eQTL can help to better understand the  molecular functional consequences of genetic variants associated with disease. To gain additional support for the  biological interpretation of these co-eQTLs, we performed a colocalization analysis overlaying the eQTL and co-eQTL  signals with the GWAS signals of the most important traits from the enrichment analysis (****Supplementary Tables  18,19****).”*

For three of the five tested loci, we observed high posterior probabilities for colocalization (PP4>0.5) of several  GWAS traits with the eQTL or co-eQTL signals (genomic loci associated with the eGenes *RPS26, HLA-DQA2* and  *RNASET2*).

For the eGene *RPS26*, we observed a high posterior probability (PP4 > 0.5) for colocalization in all cell types of the  eQTL signal with RA and asthma, while there was no indication of colocalization of the eQTL signal with Type 1  diabetes (PP4 < 0.1). We further tested if the same results can be found when testing the co-eQTL signal instead of  the eQTL signal. The co-eQTL colocalization was done separately for each co-eGene, resulting in one colocalization  probability per co-eGene. A high colocalization posterior probability (PP4 > 0.5) with almost all co-eGenes of  rs1131017-*RPS26* was found for the GWAS traits RA (n=368, 99% of significant co-eGenes) and asthma (n=323,  87% of significant co-eGenes) in CD4+ T cells. These colocalization results can be connected to lymphocyte  activation genes for RA: 6 of the 41 co-eGenes with the highest colocalization probabilities (PP4>0.9) were annotated  with lymphocyte activity and none with transcription initiation (despite many co-eGenes being associated with that  process). Colocalization signals for RA and asthma were also visible in other cell types (e.g. monocytes, see new  **Supplementary Tables 18,19**). In contrast, the colocalization signal for type 1 diabetes with the co-eQTLs was  weaker, with only 28 co-eGenes in CD4+ T having a PP4 > 0.5. We have now updated the **Results** section of the  manuscript to refine our interpretation of the *RPS26* locus following these results:

*“GWAS enrichment analysis showed enrichment for several different blood cell counts, but only in CD4+ T cells and  CD8+ T cells we also observed enrichment for immune-mediated disease (rheumatoid arthritis, Crohn's disease  (CD), multiple sclerosis (MS) and hay fever), further connecting the co-eGenes especially with T cells  (****Supplementary Table 17****). We confirmed with colocalization analyses that there is very likely a shared signal  between the eQTL and co-eQTLs signals and the GWAS signals from rheumatoid arthritis and asthma  (****Supplementary Table 18,19****). Among the co-Genes with the highest colocalization posterior probability (PP4>0.9), 6  out of 41 were again associated with lymphocyte activation. Interestingly, several studies have highlighted a  connection of RPS26 with T cell activation and survival (35,36), and the associated co-eQTL SNP rs1131017 is  associated with the enriched immune-mediated diseases (RA, CD, MS, hay fever).”*

Among the co-eQTL genes for rs9271520–*HLA-DQA2*, we found enrichment for immune-related diseases with  MAGMA, including RA, MS and asthma. For the same three diseases, we found evidence for colocalization with the  co-eQTL signal (PP4 > 0.5), at least for a substantial number of the co-eGenes (29 co-eGenes for Asthma, 21 for RA  and 2 for MS across the different cell types). We also found evidence for colocalization for the eQTL signal (PP4 >  0.5) with those GWAS traits in most cell-types (Asthma: all cell-types; MS: all cell-types except NK; RA: monocytes,  DC, B-cells). However, due to the complex LD structure in the HLA locus, a more exact mapping of causal regulatory  connections is likely not possible with our dataset. We updated the **Results** section:

*“Interestingly, rs9271520 is in LD with several SNPs associated with immune diseases, and within this locus we also  found co-eQTL enrichment for these diseases. The most significant (sorted by GWAS SNP p-values) enriched traits  include rheumatoid arthritis, MS and asthma (see* ***Supplementary Table 17*** *for full GWAS enrichment results). The*

*connection with these three diseases was also supported in our colocalization analyses, indicating colocalization in  various cell types (29 colocalizing co-eGenes for Asthma, 21 for RA, and 2 for MS; PP4>0.5;* ***Supplementary Tables  18, 19****).”*

For the locus around rs393727-*RNASET2*, high posterior probabilities were observed for colocalization of the eQTL  signal in CD4+ T cells with the GWAS signals for Crohn’s disease (PP4 = 0.90) and inflammatory bowel disease  (PP4 = 0.93). This is in line with our previous interpretation of the co-eQTL, as the co-eQTL SNP is in high LD with  GWAS SNPs associated with both traits. Additionally, the eGene and two of the co-eGenes were associated with  IBD, CD and gut immunity in previous studies (see manuscript text below). However, the colocalization signal for the  co-eQTLs was less clear with PP4 values around 0.5 for the four associated co-eGenes in CD4+ T cells. The lower  PP4 values could be caused by the limited power of our current study. We updated the **Results** section:

*“One of the potentially interesting SNP-eGenes, with too few co-eGenes to conduct proper enrichment analysis, is  rs393727 - RNASET2, which is associated with four co-eGenes (B2M, ITGB1, ALOX5AP, CRIP1). The SNP  rs393727 is in very high LD with SNPs associated with Crohn’s disease (CD) and inflammatory bowel disease (IBD)  (****Supplementary Table 16****). Additionally, the results of our colocalization analysis support a shared signal between  the eQTL rs393727 - RNASET2 in CD4+ T cells and GWAS signals for IBD (including Crohn's disease and ulcerative  colitis cases together) and more specifically only CD (****Supplementary Table 18****). For the co-eQTL colocalization, the  posterior probabilities for a shared signal were lower (around 0.5), which could be caused by a lack of power in our  current analysis (****Supplementary Table 19****). The eGene RNASET2 has previously been associated with IBD (45),  and among the four co-eGenes, ITGB1 was previously associated with CD (46) and CRIP1 with gut immunity (47),  further supporting the link of these co-eQTL genes with IBD and CD.”*

Minor remarks:

**1. Page 7 Line 24: the authors mention 187 individuals but in the rest of the paper 173 individuals are  analysed. Are some samples removed in QC? This should be described**

Yes, the observations of the reviewer are correct. For the co-expression analyses section of the paper we used 5  datasets (Oelen v2, Oelen v3, van Blokland v2, van Blokland v3 and van der Wijst dataset), comprising in total 187  individuals. Later, when we continue with the eQTL and co-expression QTL analyses, we removed one of our  datasets (the van Blokland v3 dataset), as the small sample size resulted in very few variants above the chosen MAF  cutoff of 10% and therefore the added value of this dataset was too limited. We now describe this more clearly in the  **Results** section when we introduce the co-expression analysis:

*“To identify the genetic contribution to such common and cell-type-specific effects, we performed a constrained co eQTL meta-analysis. For this, we first removed the van Blokland v3 dataset from our analyses as the small number of  donors (n=14) was too limited to provide added value for this purpose.”*

And in the **Results** section when we introduce the eQTL analysis and in the **Methods** section (both times the same  text):

*“We excluded the van Blokland v3 dataset from this eQTL analysis and all subsequent analyses because the small  sample size (N = 14) provided very few variants above the minor allele frequency (MAF) cutoff (>10%), which made it  unsuitable for this meta-analysis.”*

**2. Page 7: the description of the v2 and v3 datasets is confusing, from the text the authors seem to indicate  that individuals are measured with both v2 and v3, whereas in fact some individuals in the studies were  measured with v2 and others with v3**

Thanks for pointing this out, we formulated it clearer in the **Results** section now.

*“Two of the studies contained data that was in part generated using different versions of 10x chemistry (version 2 or  version 3, donors were measured with either version chemistry).”*

**3. Figure 1d top right panel is unclear, what are the lines and the axis?**

We replaced **Figure 1d** with a new panel describing the potential mechanism for co-eQTLs and deleted the previous  panel.

**4. The authors found a low to moderate correlation of gene co-expression between single cell and bulk RNA  datasets for the same cell types (when possible). A relatively low overlap has been found in previous studies  (e.g. PMID: 27100869), even when measuring sc and bulk in the very same cell lines. However, to validate the  results, the authors could measure whether consistent co-expression between bulk and single cell datasets  is higher for matching cell types than for non-matching cell types. This could be shown for instance for the  cell type and dataset with the most data.**

We checked how well the single-cell and bulk data correspond for non-matching cell types. For this comparison, the  bulk dataset of ImmuNexUT was taken, as it contains all major blood cell types, and the single-cell dataset from  Oelen v3. We used an expression threshold of 0.3, i.e. all genes expressed in at least 30% of the cells of a given cell  type, to include the more general highly expressed genes and the slightly lower but more cell-type-specifically  expressed genes in the comparison. As shown in the figure below, the highest correlation for each cell type (marked  with a red frame) is the matching bulk cell type, except for the CD4+ T cells, where the best matching bulk cell type  was CD8+ T cells, as expected because of high similarity between those cell types. Nevertheless, the difference  between the different bulk cell types is relatively small. A reason for this could be that cell type specific genes are  often lowly expressed and a part of them is not measured due to the sparsity of all single-cell datasets. Furthermore,  effects like the Simpson’s paradox (**Supplementary Figure 9**) are affecting any comparison, and lead to a reduction  of correlations for all comparisons.

**5. Figure 3, it would be easier to read the comparisons if the lymphoid cell types (B, T and NT) would be next  to each other. Perhaps a dendrogram on the x/y axis could be used to display the known relationship  between cell lines**

We reordered the heatmap in **Figure 3a** and added a legend to distinguish the lymphoid and myeloid lineage. The  same was done for **Supplementary Figure 12**.

**6. Page 15 Line 41: what do the authors mean by "bulk-like"? Averaging the gene expression values per  individual across all its single cells? This should be explained. Other studies call this "pseudo-bulk"**

Thanks for the suggestion. We updated the main text and call the approach now “pseudo-bulk”, as we indeed refer to  this approach, as described by the reviewer. The **Results** text with a more detailed description is now:

*“To estimate the effects of this phenomenon, we recalculated co-expression from the single-cell data using the so called pseudo-bulk approach, where we average the counts over all cells per individual, and calculated the correlation  across the individual-level counts. We compared this to the single-cell co-expression values, calculated across cells,  and observed several examples of highly expressed genes in which Simpson’s paradox occurs (Supplementary  Figure 9b,c).”*

**7. Page 22 Line 24: the authors mention a few genes with high co-expression variability across individuals.  Did the authors find co-eQTLs for those genes? If so, it would be good to mention**

We agree that looking at co-expression variability of co-eQTLs is an interesting question. In the **Results** section*,* we  assessed if co-eQTLs have higher co-expression variance than non-significant gene pairs (**Supplementary Figure  24**). Indeed, the co-eQTLs showed higher co-expression variance (except for the less frequent cell types, B cells and  dendritic cells). This is a slightly different analysis than the reviewer proposes here, but we believe that  **Supplementary Figure 24** shows the connection between co-expression variability and co-eQTLs already nicely.

**8. Page 55: the authors mention a 100kb window cutoff for lead-eQTL variants, but the variants are drawn  from the eQTLGen study (Vosa et al.). While the majority of lead eQTLs are probably within this range, why  did the authors decide to apply a further window cutoff and not test all of the lead-eQTLs? This window  should be described in the main text. Also mention how many variants were actually tested after the window  and MAF cutoff.**

We are sorry for this error and thank the reviewer for pointing this out. We edited the **Methods** section and provided a  corrected description of SNP selection there. We also included the number of tested variants in the text:

*“For these selected SNP–gene pairs, we performed eQTL mapping using eQTLPipeline v1.4.9 (62) within a  confinement of top-SNP-Gene from eQTLGen Consortium (2), using 1000 permutation rounds for determining FDR  as described in (2) and a MAF of 0.1. The eQTLGen Consortium conducted the cis-eQTL analysis with 1Mb distance.  After filtering for the MAF cutoff in our datasets, 14,009 SNPs remained in total for testing, from initially 16,290  selected SNPs from the eQTLGen Consortium. For each eGene, we first calculated the average of the normalized  gene expression level per individual for the eQTL analysis. For each dataset separately, we then calculated a  Spearman correlation coefficient for each SNP-eGene. Finally, we performed a sample size weighted meta-analysis  over all datasets to determine the meta-analysis p-value.”*

**9. It is sometimes difficult to understand which analyses are performed across individuals (e.g. single cell  correlations in Fig 2 and 3) and which analyses are performed per individual (e.g. gene co-expression per  individual for co-eQTL analysis)**

We thank the reviewer for the comment. We concatenate data from all individuals for benchmarking analysis depicted  in **Figure 2**, and the co-expression network comparison between cell types as depicted in **Figure 3a,b**. For the  analysis in **Figure 3c,d**, the comparison for individualized co-expression patterns, we calculated co-expression per  individual. For co-eQTL analysis in **Figure 4** and **Figure 5**, we calculated individualized co-expression patterns. We  updated the **Results** text for clearer explanation as follows:

*“For the comparison of co-expression networks among cell types, we merged data from all individuals before  calculating co-expression. For the comparison between individuals we calculated co-expression per individual.”*

**10. Figure 4A (and related supplementary figures): why no rb values for B cells and a few other points? In the  figure legend the authors also mention standard error but this is not represented in the figure.**

We thank the reviewer for the question. We updated **Supplementary Figure 18b,d** with standard error estimates and  we corrected the figure legend for **Figure 4**, which contains no standard error information. The reason that there is no  r_b_ value calculated for B cells is that there were too few B cells per individual detected, and thus a very limited  number of co-eQTLs was detected. Specifically, there were 35 co-eQTLs detected for B cells and hence r_b_ values  cannot be estimated reliably in this case.

We updated the legend of **Figure 4a** to make this more clear:

“a) *Replication of discovered co-eQTLs across the major cell types. Correlation of the effect sizes in replications  among different cell types, measured by r_b_ value. Text inside each block indicates the r_b_ value and number of  replicated co-eQTLs. Color intensity indicates r_b_ value. For certain cell type combinations, the number of co-eQTLs  were too few to reliably estimate r_b_ values. For those cell types only the number of co-eQTLs is shown.* ”

We also updated the **Methods** section to make it clarify why we excluded B cells for r_b_ calculation as follows: “*Additionally, in cases where fewer than 10 co-eQTLs were tested in the replication analysis, for example, B cells, we  could not get a robust estimation of the r_b_ value and hence represent them as NAs in the results section.”*

**Supplementary Figure 18**. The cell type specificity of co-eQTLs identified in each cell type. Here, we replicated the  identified co-eQTLs from each cell type in other cell types and used two measures to show the replication  performance: the ratio of tested co-eQTLs in replication, and the concordance of effect sizes shown with the rb values. Panel **a)** shows the ratio of tested co-eQTLs in replications for the identified co-eQTLs identified with the  filtering strategy. Panel **b)** shows the r_b_ values for co-eQTLs identified with the filtering strategy (standard error in  parentheses, number of co-eQTLs below). Panel **c)** shows the ratio of tested co-eQTLs in replications for the  identified co-eQTLs identified without the filtering strategy. Panel **d)** shows the r_b_ values for co-eQTLs identified  without the filtering strategy (standard error in parentheses, number of co-eQTLs below).

**11. In addition to their co-eQTL analysis, did the authors check if the eQTL of gene1 has a significant effect  directly on the co-expressed gene (gene2)?**

We thank the reviewer for the suggestion. We have now also assessed whether the eQTL SNP affects the  expression of the co-eGene for all significant co-eQTL triplets (SNP-eGene-co-eGene). Within these triplets, we  identified two unique co-eGenes also acting as an eQTL (see table below). In these two specific cases, the co-eGene  and eGene were located in close proximity of each other and their regulating SNP, As we observed this for only two

out of 946 unique co-eQTL triplets, we can conclude that in general the co-eQTL relationship is not directly regulated  by the cis-eQTL SNP.

We added a paragraph into the **Discussion** to include this additional analysis:

*“Additionally, we investigated if there is an eQTL effect between SNP and co-eGene from SNP-eGene-co-eGene  triplets. As a result, we found this to be the case for just two out of 946 distinct co-eQTL triplets, thus we may infer  that generally the co-eQTL effect is not directly controlled by the cis-eQTL SNP. In these two specific situations, the  co-eGene and eGene were located close to each other and their regulatory SNP.”*

| Cell type | Number of significant SNP-coeGene  associations | Significant SNP-coeGene  associations | Comment |
| --- | --- | --- | --- |
| CD8T | 1 | rs9271520_HLA-DQA2;HLA-DQB1 | HLA-DQA2 and HLA-DQB1 are located nearby,  therefore rs9271520 affects them both |
| DC | 1 | rs9271520_HLA-DQA2;HLA-DQB1 | similar to previous comment |
| Monocyte | 2 | rs9271520_HLA-DQA2;HLA-DQB1 rs4761234_LYZ;RP11-1143G9.4 | similar to previous comment  RP11-1143G9.4 is an antisense gene to LYZ |

**12. The "Establishing a method to identify co-expression QTLs" section is particularly long and encopasses  several topics such as the approach for (co)-eQTL mapping, identification results, comparison across cell  types and bulk eQTLs under multiple conditions, sub-cell-type composition effects etc. Perhaps it would be  better to split some of these findings into different sections?**

We updated the **Results** section by splitting the section named “*Establishing a method to identify co-expression  QTLs*” into two separate sections. The first section focuses on the co-eQTL mapping strategy and the second section  focuses on the summary statistics, cell-type comparison and replication results of the identified co-eQTLs. We  decided to keep the name for the first section as “*Establishing a method to identify co-expression QTLs*” and named  the second section as “*Meta-analysis identifies 948 co-eQTLs*”.

**13. Page 45 Line 27: The sentence "Here, the enrichment allowed us to still identify relevant TFs for further  exploration" is unclear or out of place. Also note that ReMap only contains ChIP-seq data for a fraction of all  human TFs (~150, whereas there are >1000 TFs in the genome). For instance, the gProfiler webserver/R package allows to perform enrichments for Transfac**

We had a look at the Transfac enrichments with gProfiler and compared it to our ReMap enrichments.  Conceptionally, the difference between both resources is that Transfac uses sequence motifs to identify transcription  factor binding sites, while ReMap uses ChIP-seq data. This means that Transfac does not include the information that  indicates which genomic regions are accessible in a certain cell type, so that the transcription factor can actually bind.  This is however important to better filter the large set of potential sequence motifs to a set of most likely binding sites  for our cell types of interest (for example described in the ENCODE project (11)). In contrast, ReMap uses cell  type/line specific ChIP-seq data, so information about the accessibility is provided and there is strong evidence that  the transcription factor actually binds in the respective cell type/line.

Additionally, we want to point out that we use the newest version of ReMap from 2022 (12), which contains 1,210  human TFs and is much larger than the initial release that contained 150 TFs. Even after filtering for blood-related  datasets to end up with the immune-cell-related TFs, we were left with 511 TFs that we could use for our enrichment  analysis. We updated the corresponding **Results** and **Methods** section to clarify this.

In the **Results** section, we added the version number for the ReMap database:

“*Moreover, we assessed potential common TFs regulating the shared function of these co-eGenes using ChIP-seq  data processed by ReMap 2022 (33) and found enrichment of TF binding sites in the promoter regions of co-eGenes  for 7 of the 19 SNP–eGene pairs (****Supplementary Table 15****).*”

In the **Methods** section, we added the number of analyzed TFs:

*“TF annotations were taken from ChIP-seq peaks processed in the ReMap 2022 database (33), which we filtered for  cell lines associated with blood cell lines. This resulted in a final set of 511 blood-cell related TFs that were tested.”*

As per the reviewer’s suggestion, we have now also performed the Transfac enrichment analysis, but observed far  fewer significant hits compared to the ReMap analysis (see table below). Because of this and the reasons mentioned  above, we decided to keep the original ReMap enrichment analysis in our manuscript.

Furthermore we adjusted the **Discussion** text in the manuscript to clarify this analysis:

*“By also doing an enrichment analysis for TFs regulating the set of co-eGenes, we did identify a set of candidate TFs  which are likely candidates for further investigation as potential regulators.”*

***Comparison of the number of found enrichments (FDR threshold: 0.05)***

| Number of enriched TFs  Number of enriched TFs  Cell Type SNP-eGene  ReMap  TRANSFAC | | | |
| --- | --- | --- | --- |
| B | rs1131017_RPS26 | 82 | 3 |
| CD4T | rs111454690_HLA-DRB5 | 14 | 0 |
| CD4T | rs1131017_RPS26 | 134 | 1 |
| CD4T | rs1131017_RPS26_negative | 93 | 1 |
| CD4T | rs1131017_RPS26_positive | 125 | 51 |
| CD4T | rs4147638_SMDT1 | 14 | 0 |
| CD4T | rs7605824_SH3YL1 | 58 | 1 |
| CD4T | rs7632486_CMTM8 | 4 | 0 |
| CD4T | rs9271520_HLA-DQA2 | 5 | 0 |
| CD8T | rs1131017_RPS26 | 62 | 0 |
| CD8T | rs4147638_SMDT1 | 78 | 0 |
| CD8T | rs6708265_PASK | 3 | 0 |
| CD8T | rs7605824_SH3YL1 | 9 | 0 |
| DC | rs7935082_MS4A7 | 0 | 2 |
| monocyte | rs111454690_HLA-DRB5 | 1 | 0 |
| monocyte | rs1131017_RPS26 | 145 | 40 |
| monocyte | rs9271520_HLA-DQA2 | 4 | 0 |

| NK rs1131017_RPS26 132 20 | | | |
| --- | --- | --- | --- |
| NK | rs12151742_GNLY | 0 | 1 |

**14. "Consistent with the co-eQTL overlap results, the ratio of tested co-eQTLs are generally small, ranging  between 5% to 97%": how can 97% be small? Maybe provide the median instead**

We thank the reviewer for the comment and updated the **Results** text as follows:

*“Consistent with the co-eQTL overlap results, the ratio of tested co-eQTLs are generally small, with the median value  being 37%”*

**15. Page 33: Authors should mention in the main text which genomic regions they are looking at for the TF  binding site enrichment**

We thank the reviewer for this suggestion. We updated now the **Results** section text to clarify this:  *“Moreover, we assessed potential common TFs regulating the shared function of these co-eGenes using ChIP-seq  data processed by ReMap 2022 (33) and found enrichment of TF binding sites in the promoter regions of the co eGenes for 7 of the 19 SNP–eGene pairs (****Supplementary Table 15****).” (p.33).*

The exact definition of the promoter region (2kB upstream and downstream of the first transcription start site of the  gene) is given in the **Methods** section.

**16. The authors performed extensive analysis of effect strength and direction concordance across cell types  and provided an example of positive/negative correlation in Figure 5 depending on cell type, associated with different GO term enrichments. I wonder if the authors also analysed general patterns in their findings, such**

**as the proportion of cases where a variant breaks the gene co-expression vs creating/increasing co expression. For instance, is the minor allele more often breaking or making correlations? I understand that  the paper already contains many analyses, so this is more of a curiosity question.**

We thank the reviewer for the question. **Supplementary Figure 19** shows Z-score distribution for the CD4+ T cell co eQTLs for each dataset on the diagonal. When calculating co-eQTLs, we used the minor allele to assign direction of  the genetic effect. As a consequence, a positive direction means that the co-expression increases for additional  copies of the minor allele, and a negative direction means that the co-expression decreases. For the CD4+ T cells co eQTLs, the positive and negative direction of effect is relatively balanced. We also checked the same for all other cell  types: the positive co-eQTLs were more frequent in all other cell types except the CD8+ T cells. However, all co eQTL sets were strongly dominated by the co-eQTLs associated with rs1131017–*RPS26* (many co-eQTLs  associated with this eQTL). This makes an overall interpretation of co-eQTL direction difficult. For this reason, we  only focused on the direction of effect for the co-eQTLs in rs1131017–*RPS26*.

Reviewer #2:

**In this paper, the authors performed a comprehensive analysis of gene co-expression patterns for different  cell types inferred from single cell data across five data sets, and co-eQTL analysis to identify SNPs whose  genotypes are associated with cell-type-specific correlations between certain gene pairs. The co-eQTL  analysis is challenging due to the very large search space. The authors used results from previous eQTL  analysis to limit the number of SNPs to be considered for each gene, i.e. one SNP per gene. They further  limited the number of gene pairs to those that only include at least one eGene and having significant co expressions in at least 10% of the subjects. This stringent filtering strategy reduced the multiple comparison  burden and allowed the authors to identify statistically significant co-eQTLs for different cell types. The  authors conducted GO enrichment, motif, GWAS enrichment analysis to demonstrate that the results are  credible, with detailed discussion on rs1131017-RPS26 and co-eGenes.**

**Overall, the statistical and bioinformatics analyses presented in this paper are extensive and the results  suggest that there may be novel biological insights learned from co-eQTL analysis. However, the biological  interpretations are largely speculative without experimental validations and the RPS26 example is not as  interesting. I have the following specific comments.**

We thank the reviewer for the helpful comments and suggestions. As we have already extensively discussed in the  “**Comments raised by both reviewers”** section of this rebuttal, we want to emphasize that one main focus of our  manuscript is the methodological aspect. We evaluated and improved the previous co-eQTL mapping and  interpretation strategy, including a systematic examination of different technical aspects, such as the number of cells  and individuals, which can provide concrete guidance for future co-eQTL study design. We applied the improved  strategy on four datasets from three published single cell studies and uncovered a potential cell-type-specific genetic  regulation mechanism for a GWAS locus that has been previously associated with various autoimmune diseases.  We plan to apply our optimized strategy in larger studies in the near future, in particular within the sc-eQTLgen  consortium (8) and expect that the larger number of co-eQTLs will reveal additional exciting biology.

We strongly disagree that our interpretation is only speculative. Although we do not perform experimental validation  of the findings, we did re-analyze a multitude of functional genomics (e.g. TF ChIP-seq) and GWAS datasets, which  provide combined evidence and generate data-driven hypotheses. To further extend the strength of our interpretation,  we have now added a colocalization analysis for a number of loci of interest (five sets of co-eQTLs with the following  eGenes: *RPS26, HLA-DQA, RNASET2, SMDT1* and *TMEM176A*; see Reviewer 1, major remark 6). An experimental  validation is unfortunately beyond the scope of our project at the moment, but the data-driven hypotheses generated  with this analysis prioritize candidates with strong support from multiple data sources for experimental follow up  studies.

**[1] A large portion of the paper is devoted to the justification of using Spearman correlations as a measure of  co-expression for single cell data, and the validity of co-eQTL analysis hinges on the ability of Spearman  correlation to appropriately characterize/quantify co-expression between genes. As acknowledged by the  authors, single cell data are very sparse and different cells can have substantial differences in sequencing  depths. Simple adoption of Spearman correlation may be confounded with sequencing depth, and highly  expressed genes may be more likely found to be co-expressed with other genes. It is not clear how much  this bias may contribute to the identifications of co-eGenes for the rs1131017-RPS26 example.**

We thank the reviewer for the comments. Despite the sparsity of scRNA-seq data, we chose the Spearman  correlation over other more sophisticated strategies because of its robustness and well-understood statistical  properties. A recent benchmarking paper (13) also included the surprising finding that Pearson’s correlation is  among the top-performing methods across datasets. As described in the **Supplementary Text**, we tested the  following strategies:

1) Proportionality: We chose to test proportionality because a recent benchmarking paper (14) identified the  proportionality measurement as the best performing correlation metric. However, we observed that this

metric gives comparable results with Spearman correlation and it creates spurious high correlations for lowly  expressed genes (**Supplementary Figure 2**). Additionally, it was much more computationally intensive than  Spearman correlation calculation.

2) GRNBoost2: We chose this method because a recent benchmarking paper (15) about GRN construction  identified it as the top-performing method that is also scalable. However, this method resulted in very  different co-expression patterns when we compared between single cell and bulk data (**Supplementary  Figure 5**), which makes any further interpretation dataset-specific and not generalizable.

3) Metacell: We tested MetaCell to specifically address the sparsity problem introduced by single cell data. The  original algorithm resulted in too few meta cells per individual and we couldn’t get robust co-expression  estimates. We implemented this strategy with customized scripts using Leiden clustering to overcome the  limitations of the original MetaCell algorithm. We estimated co-expression by Spearman correlation between  meta cell expression levels and compared it to the co-expression using bulk co-expression estimations from  BLUEPRINT data. However, we observed that the performance was comparable and sometimes worse than  Spearman correlation on individual cells (**Supplementary Figure 3**).

We also reiterate these reasons in the **Discussion** as follows:

*“We tested other association methods, including the proportionality measure and GRNBoost2, that were recognized  as top-performing in independent benchmarking studies. However, they did not perform better in our validation.  Additionally, we tried several pseudotime ordering methods because many GRN reconstruction methods required  time-stamped information. However, the obtained pseudotime orderings differed among methods (Supplementary  Text, Supplementary Figure 4). We therefore applied Spearman correlation as a solid basis for the co-eQTL analysis.  However, we do acknowledge that Spearman correlation may not be the ideal method to handle scRNA-seq data due  to the sparseness. Future work may find that other association measures are equally suitable or more suitable, and  this may potentially depend on the specific single-cell dataset under investigation.”*

For the other questions regarding the sequencing depth and expression level biasing the co-eQTL analysis, we argue  that this is unlikely the case because:

1) The scRNA-seq data were normalized using SCTransform from the Seurat package. This normalization  method removes the technical effects with a generalized linear model utilizing sequencing depth as  covariate. Therefore we do not expect that sequencing depth heavily influences the co-eQTL mapping  analysis. However, we have noticed that in the previous version of the manuscript, we did not describe this  step. We apologize for this omission and have now updated the **Methods** section describing the correct  normalization steps we utilized as follows:

“*For co-expression quantification, we used SCTransform from the Seurat package and used the log  transformed counts as input. We then calculated the Spearman correlation of gene pairs in the three  different single-cell studies (Oelen dataset (11), van Blokland dataset (19) and van der Wijst dataset (10))  and then compared between datasets and 10XGenomics chemistry.*”

2) We fully acknowledge that the highly expressed genes are more likely to be picked up as co-eGenes, as  shown in **Supplementary Figure 24.** However, we argue that sufficient expression levels are essential for  co-expression patterns to be accurately measured.

3) We performed a co-eQTL meta-analysis using 4 different datasets from 3 different studies. The datasets  have different sequencing depths and gene expression level distributions. Nevertheless, we have observed  high concordance in effect sizes among these datasets (**Supplementary Figure 19**) and we have seen high  concordance with the replication in a bulk dataset, providing additional confidence that the identified co eQTLs are not due to technical variation.

We fully acknowledge that these technical factors could bias the co-eQTL identification, however, we argue that our  normalization steps, replication and interpretation pipelines ensured that the signals we picked up are unlikely due to  these technical factors. We have updated the **Discussion** section about the potential influence of confounding  factors:

“*Several confounding factors such as sequencing depth, number of cells and sub-cell-type composition could  influence the results. In this study, we used SCT normalization for the scRNA-seq data to reduce the impact of  sequencing depth on downstream analysis. We normalized the individualized co-expression values by the number of*

*cells per individual and calculated z-statistics (see* ***Methods****) for co-eQTL mapping. In addition, fine grained  compositional differences within a cell-type can introduce false positive co-eQTLs within a cell type if a genetic variant  influences this composition and one of the tested genes shows differences in expression that also depend on this  composition. However, in our evaluation of classical and non-classical monocytes, we observed no strong  confounding of monocyte co-eQTLs by the subtypes of cells (****Supplementary Figure 24****). Although we cannot fully  rule out all other possible confounding factors that could potentially create false positive findings, we have shown that  the identified co-eQTLs show highly consistent effect sizes and directions across all datasets used in our meta analysis and also in the independent bulk replication cohort. This indicates that the majority of our reported co-eQTLs  are not due to batch effects. However, we do encourage future studies to further examine potential confounding  factors to further reduce the possibilities of false positives and false negatives.*”

**[2] For co-eQTL analysis, when co-expression is not significant between two genes for an individual, what's  the input for the analysis? Did the authors use 0 or the calculated Spearman correlation?**

We always use the observed co-expression (Spearman correlation). We do not impute insignificant nor missing co expression values to 0. As shown in **Supplementary Figure 16**, this could create false positives and inflate our  results. We updated the **Results** section in the manuscript to give a clearer explanation as follows:

*“As we observed that replacing missing values with 0 can lead to spurious co-eQTL results (****Supplementary Figure  16****), we remove the missing correlations and do not impute the missing correlation to 0 when mapping co-eQTLs.”*

**[3] For replication studies using bulk samples, could the authors also use the data from the sorted cells from  BLUEPRINT and ImmuNexUT, in addition to the BIOS data?**

We thank the reviewer for the suggestion. However, we consider the following:

1) To replicate the co-eQTLs in bulk data, gene-genotype interaction models have to be used: inter-individual  correlation cannot be calculated without multiple measurements per individual. In scRNA-seq data this is  resolved because multiple single cells are measured per individual. Interaction effects are generally much  weaker than generic eQTL effects. As such, in bulk data, such an analysis requires a large number of  samples. The BIOS dataset has 2,491 individuals while the BLUEPRINT and ImmuNexUT have a few  hundred individuals, and thus we argue that the BIOS dataset is best powered for this replication analysis.

2) The replication analysis requires individual genotype data, which is not publicly available for BLUEPRINT  and ImmuNexUT

Therefore, we reason that our replication analysis using the BIOS dataset gives sufficient evidence for the robustness  of the identified co-eQTLs in single cell data. We have now updated the **Results** section as follows: “*We only considered BIOS, rather than the BLUEPRINT and ImmuNexUT, as a replication dataset because BIOS  has 2,491 individuals while the other two only have a few hundred individuals*.”

**[4] For enrichment analyses on pages 34 and 35, the authors used all genes tested in the co-eQTL analysis in  the respective cell type as background control. Is this an appropriate control? Can the authors use the genes  that failed to be identified as co-eGenes for a given eQTL-eGene to perform a similar enrichment analysis  and see whether this set may also be enriched for some GO categories?**

In general, different definitions of the background set lead to different null hypotheses. When using all genes that  were tested in the co-eQTL analysis for the respective cell type as the background set of the GO analysis, this would  ask: ‘Are there more genes annotated to a specific GO term linked to a specific co-eQTL locus than we would expect  among all genes that could have been detected to be linked to **any** co-eQTL locus?’ Another option would be to make  the question more specific by using a background set specific for each co-eQTL locus by including all genes tested  for the respective co-eQTL in the cell type (i.e. genes that are correlated with the eGene at least 10% of the  individuals). In this case, this would ask the question ‘Are there more genes annotated to a specific GO term linked to  a specific co-eQTL locus than we would expect among all genes that could have been detected to be linked to **this  specific** co-eQTL locus?’ We believe that both questions can yield valuable insights.

Motivated by the reviewer’s question, we tested the enrichment analysis with different background sets (the basic  background set and the more specific background set, as defined in the previous paragraph) and also checked the  enrichment using genes that failed to be identified as co-eGenes. This led to four different scenarios: **Scenario 1:** using the basic background set and enrichment among significant co-eGenes (the one reported in the  manuscript)

**Scenario 2:** using the strict background set and enrichment among significant co-eGenes **Scenario 3:** using the basic background set and enrichment among the non-significant co-eGenes (as suggested in  the second question of this remark)

**Scenario 4:** using the strict background set and enrichment among the non-significant co-eGenes

First, we compared the enrichment analysis among significant co-eGenes with different background sets (**Scenario 1  and 2**). The more specific background (**Scenario 2**) reduced the number of significant GO terms: the number of SNP eGene pairs per cell type, where the co-eGenes were enriched for at least one GO term, was reduced from 30 to 20.

However, for the interesting examples we mentioned in our result section, the results stayed very similar. For  example, the top five enriched GO terms of the GO ontology “Biological Process” for positive and negative coeGenes  for rs1131017-*RPS26* (shown in **Figure 5e,f**) are not in all cases in the top five enriched GO terms anymore for the  restricted background, but all ten terms are still significantly enriched with the reduced background set. Only for  another example, the enrichment of rs7806458–*TMEM176B,* the GO term “complement component C3b binding” is  not significant anymore with the restricted background.

We added a paragraph about the background sets into the **Discussion** of our manuscript: *“The choice of the background set for the enrichment analyses affects the interpretation. We decided to include all  genes tested for co-eGenes in the respective cell type into the background gene set. We also explored the effect of a  more specific background, which focused on the genes tested for the respective SNP-eGene in the cell type* ***(Supplementary Table 20)****. The total number of enriched pairs was reduced slightly with the more specific  background, but the enrichment results for rs1131017-RPS26 and our other examples in the results section stayed  very similar and the changes did not affect our interpretation.*”

We also explored the second suggestion of the reviewer and additionally tested whether we find significant  enrichment among the genes that were tested as co-eGenes but for which no significant association was found  (**Scenario 3 and 4**). Using the more broad background set (**Scenario 3**), we find enrichment for 29 SNP-eGenes  pairs per cell type. Using the more specific background set (**Scenario 4**), we find enrichment for 6 SNP-eGenes per  cell type. Interestingly, for the specific background, most enriched GO terms were associated with the *RPS26* locus in  monocytes and NK cells, where terms like “immune system” and “lymphocyte activation” came up. For CD4+ T and  CD8T cells, there was one depleted term for the *RPS26* locus in each cell type: one involving the catalytic complex  and one involving cytoskeleton. This supports our enrichment results that showed an association with RPS26 and  lymphocyte activation in T cells, but not in Monocytes and NK cells. We however decided to not include these results  into the manuscript because the results were hard to interpret due to the nature of scenario 3 and 3 being effectively  depletion analyses.

References

1. Oelen R, de Vries DH, Brugge H, Gordon MG, Vochteloo M, Ye CJ, et al. Single-cell RNA sequencing of peripheral blood mononuclear cells reveals widespread, context-specific  gene expression regulation upon pathogenic exposure. Nat Commun. 2022 Jun  7;13(1):3267.

2. Okada Y, Wu D, Trynka G, Raj T, Terao C, Ikari K, et al. Genetics of rheumatoid arthritis  contributes to biology and drug discovery. Nature. 2014 Feb;506(7488):376–81.

3. Laufer VA, Tiwari HK, Reynolds RJ, Danila MI, Wang J, Edberg JC, et al. Genetic influences  on susceptibility to rheumatoid arthritis in African-Americans. Hum Mol Genet. 2019 Mar  1;28(5):858–74.

4. Ni J, Wang P, Yin KJ, Yang XK, Cen H, Sui C, et al. Novel insight into the aetiology of  rheumatoid arthritis gained by a cross-tissue transcriptome-wide association study. RMD  Open. 2022 Sep 8;8(2):e002529.

5. Kasela S, Kisand K, Tserel L, Kaleviste E, Remm A, Fischer K, et al. Pathogenic  implications for autoimmune mechanisms derived by comparative eQTL analysis of CD4+  versus CD8+ T cells. PLOS Genet. 2017 Mar 1;13(3):e1006643.

6. Chen C, Peng J, Ma S, Ding Y, Huang T, Zhao S, et al. Ribosomal protein S26 serves as a  checkpoint of T-cell survival and homeostasis in a p53-dependent manner. Cell Mol  Immunol. 2021 Jul;18(7):1844–6.

7. van der Wijst MGP, Brugge H, de Vries DH, Deelen P, Swertz MA, LifeLines Cohort Study,  et al. Single-cell RNA sequencing identifies celltype-specific cis-eQTLs and co-expression  QTLs. Nat Genet. 2018 Apr;50(4):493–7.

8. van der Wijst M, de Vries D, Groot H, Trynka G, Hon C, Bonder M, et al. The single-cell  eQTLGen consortium. eLife. 2020 Mar 9;9:e52155.

9. Stegle O, Parts L, Durbin R, Winn J. A Bayesian Framework to Account for Complex Non Genetic Factors in Gene Expression Levels Greatly Increases Power in eQTL Studies.  PLOS Comput Biol. 2010 Jun 5;6(5):e1000770.

10. Hao Y, Hao S, Andersen-Nissen E, Mauck WM, Zheng S, Butler A, et al. Integrated analysis  of multimodal single-cell data. Cell. 2021 Jun;184(13):3573-3587.e29.

11. Moore JE, Purcaro MJ, Pratt HE, Epstein CB, Shoresh N, Adrian J, et al. Expanded  encyclopaedias of DNA elements in the human and mouse genomes. Nature. 2020  Jul;583(7818):699–710.

12. Hammal F, de Langen P, Bergon A, Lopez F, Ballester B. ReMap 2022: a database of  Human, Mouse, Drosophila and Arabidopsis regulatory regions from an integrative analysis  of DNA-binding sequencing experiments. Nucleic Acids Res. 2022 Jan 7;50(D1):D316–25.

13. Stone M, Li J, McCalla SG, Siahpirani AF, Periyasamy V, Shin J, et al. Identifying strengths  and weaknesses of methods for computational network inference from single cell RNA-seq

data [Internet]. bioRxiv; 2022 [cited 2022 Dec 2]. p. 2021.06.01.446671. Available from:  https://www.biorxiv.org/content/10.1101/2021.06.01.446671v4

14. Skinnider MA, Squair JW, Foster LJ. Evaluating measures of association for single-cell  transcriptomics. Nat Methods. 2019 May;16(5):381–6.

15. Pratapa A, Jalihal AP, Law JN, Bharadwaj A, Murali TM. Benchmarking algorithms for gene  regulatory network inference from single-cell transcriptomic data. Nat Methods. 2020  Feb;17(2):147–54.

**Second round of review**

**Reviewer 1:** The authors have revised the manuscript and replied successfully to all concerns raised.

**Reviewer 2:** The authors have addressed my comments.
